# Deep landscape update of dispersed and tandem repeats in the genome model of the red jungle fowl, *Gallus gallus*, using a series of de novo investigating tools

**DOI:** 10.1186/s12864-016-3015-5

**Published:** 2016-08-19

**Authors:** Sébastien Guizard, Benoît Piégu, Peter Arensburger, Florian Guillou, Yves Bigot

**Affiliations:** 1 Physiologie de la Reproduction et des Comportements, UMR INRA-CNRS 7247, PRC, 37380 Nouzilly, France; 2 Biological Sciences Department, California State Polytechnic University, Pomona, CA 91768 USA

**Keywords:** Satellite DNA, Transposable elements, Bioinformatics, Benchmarking, Repeat

## Abstract

**Background:**

The program RepeatMasker and the database Repbase-ISB are part of the most widely used strategy for annotating repeats in animal genomes. They have been used to show that avian genomes have a lower repeat content (8–12 %) than the sequenced genomes of many vertebrate species (30–55 %). However, the efficiency of such a library-based strategies is dependent on the quality and completeness of the sequences in the database that is used. An alternative to these library based methods are methods that identify repeats de novo. These alternative methods have existed for a least a decade and may be more powerful than the library based methods. We have used an annotation strategy involving several complementary de novo tools to determine the repeat content of the model genome galGal4 (1.04 Gbp), including identifying simple sequence repeats (SSRs), tandem repeats and transposable elements (TEs).

**Results:**

We annotated over one Gbp. of the galGal4 genome and showed that it is composed of approximately 19 % SSRs and TEs repeats. Furthermore, we estimate that the actual genome of the red jungle fowl contains about 31–35 % repeats. We find that library-based methods tend to overestimate TE diversity. These results have a major impact on the current understanding of repeats distributions throughout chromosomes in the red jungle fowl.

**Conclusions:**

Our results are a proof of concept of the reliability of using de novo tools to annotate repeats in large animal genomes. They have also revealed issues that will need to be resolved in order to develop gold-standard methodologies for annotating repeats in eukaryote genomes.

**Electronic supplementary material:**

The online version of this article (doi:10.1186/s12864-016-3015-5) contains supplementary material, which is available to authorized users.

## Background

Repeated sequences are the most abundant components of many eukaryote genomes. They account for approximately 25 % of the fruit fly (*Drosophila melanogaster*) genome [1, 2], 50–69 % of the human genome [3] and nearly 90 % of the maize (*Zea mays*) genome [4]. Repeated sequences in eukaryotic genomes vary in their structure, organization and location in chromosomes. The primary criterion is often their distribution profile in chromosomes, that is, their organization in stretches of tandem repeats or as interspersed copies.

The most highly repeated sequences generally lie near or within centromeres and telomeres. Tandem repeats within a chromosome segment may contain tens to several thousands of units. These are composed of two main types: 1) stretches of (TTAGGG)n repeats at telomere ends [5], and 2) satellite DNAs composed of tandem repeated units of 60 to a few thousand bp. Eukaryote genomes may contain one or more families of satellite DNA. The sequence of the repeated units and the abundance of each family are generally specific to each species [6, 7].

Another type of tandem repeat, found in the inner regions of the chromosome arms, are the simple sequence repeats (SSRs); these may be divided into several groups. The first group includes short stretches of tandem repeats with low complexity sequences that are dispersed along chromosomes. This group has further been subdivided into three types depending on the complexity of the repeated unit. The first type are simple repeats, stretches of A and T or C and G nucleotides. The second type gathers micro and minisatellites (also called variable number tandem repeats (VNTRs)) that are 2 to 10 bp. (micro) or 11 to 60 bp. (mini) long sequence repeats [8, 9]. The final type are segmental duplications, these result from the duplication of chromosome segments and are often associated with tandemly repeated genes such as those encoding ribosomal RNA (rRNA) and immunoglobulins. In this last repeat type, when the number of tandem repeats varies between individual alleles within a species they are know as copy number variations (CNVs) [10, 11].

The dispersed nature of a large subsection of repeats is generally the result of their ability to move from one locus to another using a variety of transposition mechanisms including “cut-and-paste”. Furthermore, these repeats may also be amplified within chromosomes by transposing using a “copy-and-paste” mechanism. The diversity, origin and classifications of these repeats is the subject of ongoing research (see [12] for a review). However, the vast majority of dispersed repeated sequences in eukaryotes are likely to be transposable elements (TEs). TEs so far described in avian genomes can be grouped into four groups based on their sequence organisation (reviewed in [12]). Three of these groups include TEs that use RNAs as a transposition intermediate and have previously been classified as Class 1 elements. In this case the RNA molecule is transcribed from a genomic copy that will later be reverse-transcribed into a DNA molecule during, or prior to, insertion at a new chromosomal site. The first of these are the LTR retrotransposons TEs and endogenous retroviruses. These contain long terminal repeats (LTR) and three open reading frames that encode a group-specific antigen (Gag), a reverse transcriptase (RT), and a retroviral envelope protein (Env). The second group of TEs that use RNAs as a transposition intermediate are the non-LTR retrotransposons, also known as retroposons or long interspersed elements (LINEs). These TEs have no terminal repeats and two open reading frames that encode proteins similar to the Gag and RT proteins mentioned above. The third TE group that uses an RNA intermediate are the short interspersed elements (SINEs) that are derived from the transcripts of host genes that encode structural RNA molecules (tRNA, 7SL RNA, 5S RNA, 28S, snRNA). SINEs are not able to move autonomously but rely on the transposition machinery of certain non-LTR retrotransposons. The fourth, and final group of TEs do not use an RNA intermediate for movement. Instead, they use a single or a double-stranded DNA molecule as a transposition intermediate [12]. This intermediate is either excised or produced by DNA replication from a genomic copy and then inserted at a new chromosomal site. These TEs, commonly known as DNA transposons, were previously gathered in what was called Class 2 elements. We will refer to them here as "terminal inverted repeats (TIR)" elements because they display terminal inverted repeats at their ends.

Because repeats are often abundant in eukaryotic genomes, annotating them requires considerable effort. TEs are a particular challenge because eukaryotic genomes generally contain between tens to hundreds of different TE “species” and the abundance of each one may vary considerably. Despite this diversity, only a few individual copies within some of these “TE species” are actively transposed. The vast majority are inactive remnant copies with sequences that have accumulated a number of nucleotide mutations and rearrangements over time, depending on the age of each “TE species” in its host genome. There is currently no reliable and validated strategy for locating and annotating repeats in eukaryotic genomes. This problem has recently been the subject of a call for benchmarking of methods for annotating transposable element in order to optimize reporting of the efficiency of each method and to clarify the nature of the problems encountered [13]. The three most commonly used approaches are: library-based methods, signature-based methods, and de novo consensus methods (see [14, 15] for a review). RepeatMasker (RM) is the most widely used library-based method in genome sequencing projects [16, 17] and is typically used in association with Repbase, a repeat library that is freely available to academics [18]. The TEs of numerous genomes have been annotated with RM and a private, inaccessible, library at the Institute for System Biology (ISB) [19]. The main limitation of such library-based approaches is that the annotations depend very heavily on the quality of the reference database, including completeness and accuracy of the consensus sequences. By contrast, signature based methods focus on traits that are unique to certain TEs or repeats. For example, the program LTR Finder detects specific DNA organization patterns as well as a chain of signatures (motifs) specific to retroviruses to detect LTR-retrotransposons [20]. Tandem repeat finder (TRF), another signature method tool, is dedicated to detecting all types of uncomplicated tandem repeats such as simple repeats, microsatellites, minisatellites and satellite DNAs [21]. Finally, DNA de novo consensus methods combine a range of detection tools. The REPET package is a pipeline that uses both de novo and signature-based methods [22–24] and may be used to include a library-based step [25]. de novo consensus methods such as REPET have been limited until now by their need for powerful resources for calculation and storage which has restricted their application to small eukaryotic genomes (~10 Mbp to 500 Mbp). However, advances with computing clusters and a recent REPET update have opened the way for the use of this software package with larger genomes such as those of vertebrates.

**Fig. 1:**
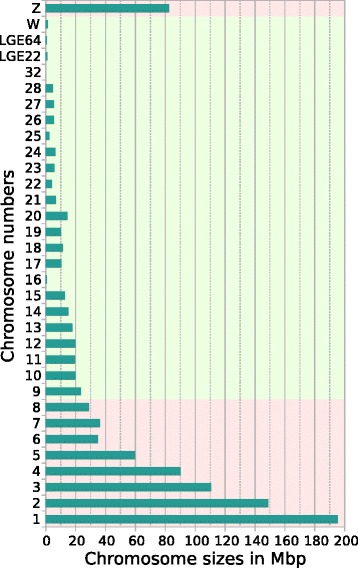
Sizes of chromosomes in galGal4. Backgound areas in pink indicate macrochromosomes and those in green indicate microchromosomes. Note, the chromosome numbering set up in caryology does not form a decreasing series in size with galGal4 for chromosomes 6, 11, 15, 16, 17, 18, 19, 22, 23, and 25. LGE22 (LGE22C19W28_E50C23) and LGE64 are two linkage groups whose scaffolds are assembled in two chromosomes but currently have no assigned microchromosomes. The 29 assembled chromosomes plus the two sex chromosomes W and Z and the two LGE contain a total of 1,004,801,586 Mbp. There is also a U chromosome not shown in the graph which contains all the unplaced scaffolds (14,093 sequences containing 42,130,513 bp)

Our work has focused on the analysis of repeats in the smallest vertebrate genome (just over 1 Gbp): the red jungle fowl (RJF) *Gallus gallus*. Avian genomes (with the exception of some Falconiforme species [26]) are composed of a several macrochromosomes (RJF has 9: 1 to 8, depending on their physical size, plus the Z sex chromosome), and many microchromosomes (RJF has 30 : 9 to 38, plus the W sex chromosome) [27]. The RJF genome was the third vertebrate genome to be sequenced and is one of the few vertebrate genomes for which a physical map was used to construct the first version of the genome model called galGal1 [28]. This genome model was then improved in several steps [23–31] until the release of galGal4 in November 2011 [32]. None of these models may be considered to be definitive, new updates are regularly published, and galGal4 must be considered only as an imperfect model of the actual RJF genome. The size of the RJF genome, its C-value which reflects the amount of nuclear DNA in the haploid genome, has been estimated to be 1.25 ± 0.06 pg by reassociation kinetics [33, 34] and flow cytometry [35–38]. Comparison of the RJF genome size to galGal4 can be accomplished by converting the C-value to an absolute number of bp [39]. This yields a size of 1.223 ± 0.058 Gbp for the RJF genome while that of the galGal4 model is only 1.047 Gbp (including 14 Mbp of gaps that are filled by 'N-stretches'), a size difference of 175 Mbp (14 % of the RJF genome size). The origins of this size difference may come from various sources. First, there are likely to be missing sequences in the galGal4 model because nearly all the regions overlapping the megacentromeres [40] and megatelomeres [41–43], and their neighbouring satellite DNAs [44] are absent from the model. This has been estimated to account for approximately 8 % of the RJF genome [40–44]. Furthermore, galGal4 does not appear to contain tandem repeats encoding the 18S-5.2S-28S (~400 copies) and 5S (~100 copies) rRNAs [45]. This represents approximately 1 % of the RJF genome. A third possible source of the size difference is that features that are located in AT-rich, GC-rich, or regions containing short motifs are not always properly represented in libraries based on Illumina technology [46–52]. Such sequences are likely responsible in part for the 8 unassembled chromosomes in galGal4 (numbers 30, 31, 33, 34, 35, 36, 37 and 38, two of which correspond to LGE22 and LGE64; Fig. 1) [28, 32]. They could also be the source of chromosome 32 (1028 bp) and explain the small size of chromosome 16 (535,270 bp in the model; with an actual size estimated to be close to 11 Mbp [53]) because of its high repeat content. This may also be responsible for the fact that most of the other chromosomes in galGal4 (Fig. 1) are smaller than those of the RJF genome [54], and probably for the fact that avian genomes lack at least some of the ~6000 protein-coding genes that are present in all mammals [32, 55–57].

A clear understanding of the reasons behind the size differences between the galGal4 model and the RJF genome is important in order to understand where the model has failed and how it might be improved. Reassociation kinetics indicate that the RJF genome contains approximately 32 % repeats [58, 59]. As the galGal4 model lacks centromere sequences, telomere, the clusters encoding the rRNA and a part of the satellite DNA, the total rate of repeats in the genome model is estimated to be between 22–24 %. Successive investigations, mainly using the RM library based method, have reported repeat percentages that have gradually increased over time: 9.5 % in 2004 [28], 8 % in 2005 [60], and 11.47 % in 2011 [61] (Table 1), but are still significantly lower than those calculated from DNA reassociation kinetics. This suggests that the analysis of repeats in the galGal4 assembly needs further investigation.

**Table 1:** Proportion of repeated sequences reported in the chicken

Methods	% of moderately repeated and interspersed sequences	% of highly repeated sequences	Year of publication	References
Reassociation kinetic	20	10	1978	58
Reassociation kinetic	20	10	1978	59
ICGGC^a^	9.4	0.1	2004	28
Reassociation kinetic and sequencing	4.3	3 to 4	2005	60
ISB	9.74	1.73	2011	61

^a^International Chicken Genome Sequencing Consortium

We have re-investigated the status of repeats in the galGal4 assembly using mainly a de novo annotation strategy that involves several complementary methods of detection and annotation. We detected repeats in the galGal4 model in numbers that are closer to those predicted by physicochemical data. Analysis of these new annotations sheds new light on the genome in terms of how its components are organised, including TE diversity, distribution, and dynamics. Finally, we discuss the benchmarking of various methods used in our investigations in the hope of stimulating debate that may lead to the definition of a gold standard for annotating repeats in assembled genome models.

## Results and discussion

### Evaluating the proportion of repeats in the galGal4 model in silico

It is important to be able to accurately assess the amount of repetitions in order to properly annotate a genome. DNA reassociation kinetics can be used to estimate a conservative proportion of repeats. Indeed, the 22–24 % repeat proportion estimated for the RJF is only a minimal value because its calculation is limited by two parameters in the experimental procedure [62]. First, the ability of this technique to detect repeats in a genome depends on the length of the fragments used (generally 200–250 bp). Second many of the repeated sequences in a genome such as that of the RJF are old [61]. Because these old repeats are likely to have drifted significantly over time it may be assumed that a certain proportion of them will be recovered in the unique component of the DNA reassociation kinetics results. In some cases, studies that used more stringent reassociation conditions found an average repeat rate of 13 % in the RJF genome [34]. An advantage of some *in silico* approaches is that they can detect very short sequences. Indeed, these methods can be calibrated to be insensitive to the minimum size of repeated sequences as well to their sequence divergence. We selected two such methods, P-clouds [63] and Red [15] (Additional file 1).

The overall proportion of repeats in the galGal4 model detected with P-clouds (33 %) and Red (29.9 %) were similar, but were also approximately 50 % higher than the values obtained with DNA reassociation kinetics. As positive controls we tested the reliability of both methods using two published genomes with well-established repeat content: *Anopheles gambiae* (mosquito) and *Drosophila melanogaster* (fruit fly). Analysis of these genomes was facilitated by the fact that their “TE species” sequences are well-conserved. We found that in these control genomes Red was the most appropriate program for calculating a reliable rate of repeats because it recovered a substantially larger proportion of previously annotated repeats (84 %) than P-clouds (61 %) (Additional file 2).

### Detection and annotation of repeats in galGal4

#### Strategy for detecting and annotating repeats in galGal4

**Fig. 2:**
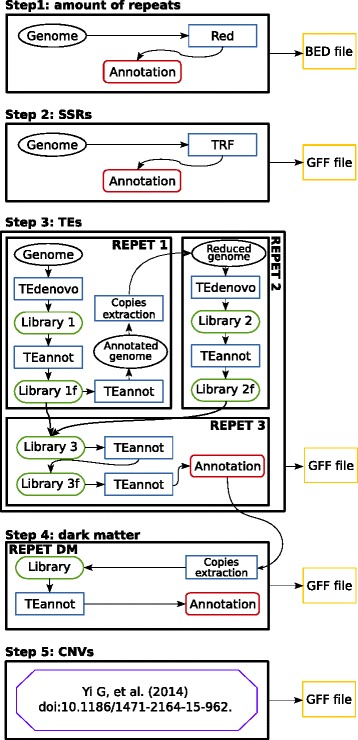
Strategy for detecting and annotating repeats in galGal4. Our strategy comprised five successive steps: 1, definition of the number of repeats; 2, number of SSRs; 3, number of TEs; 4, definition of dark matter; 5, definition of CNVs. The final products of each of these 5 steps were stored in the bed or gff annotation files (*yellow* boxes). Arrows show the chronology of events in the processes in each step. Black ellipses show the various states of the genome analysed; *blue* boxes indicate the programs used; *green* boxes indicate the intermediate library produced by a given process; *red* boxes indicate the end of a process before editing the bed or gff annotation files. The *purple* box in step 5 indicates the source of the annotation file used for CNVs

Our approach for accurately estimating the repeat content of the galGal4 model was based on published data and analysis of individual repeat types (such as those described above, as well as others methods that are detailed below). The resulting strategy (Fig. 2) was organized into five steps. First used the program Red to estimate the total number of repeats [15]. Second, TRF was used to analyse SSRs [21]. Third was the TE annotation, which demanded the most investment of resources. We used the software package REPET [22–24] because it has been extensively tested and had been shown to be more efficient than the RepeatScout [64] and RepeatModeler [65] packages. We were aware that REPET annotations do not always recover 100 % of annotations calculated by the other two packages [13], but decided that these were ultimately only small differences. Furthermore, we found that even these small differences were minimized by our use of TRF prior to REPET, which we found to be more efficient at locating SSRs than either the REPET. We performed the REPET analysis in three successive detection steps (Fig. 2) in order to dig deeper for fragmented repeats than RM. Our fourth step in the annotation strategy was to annotate the dark matter (DM) as proposed by Maumus et al (2014) [25], using a library containing all repeated copies longer than 500 bp detected in step 3 and the TEannot program [66] rather than RepeatMasker (RM) [67]. For our final step we used the available annotation of CNVs in galGal4 [11, 68].

#### Profiles of SSRs in galGal4 (STEP2)

**Fig. 3:**
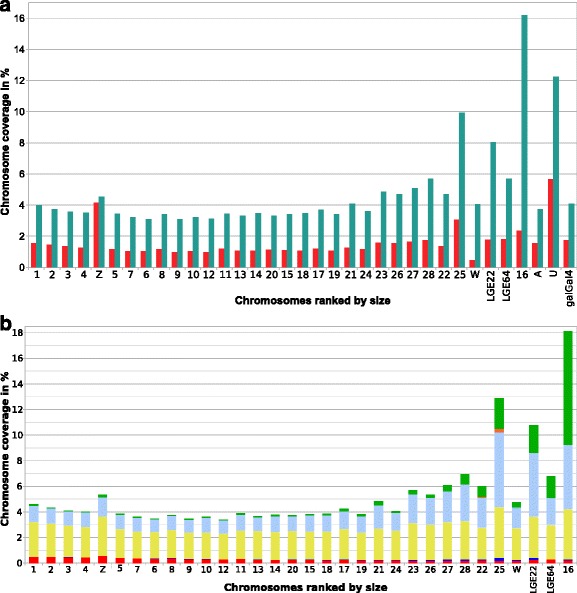
Features of SSRs in galGal4. **a** Coverage of former and new SRR annotations among chromosomes and galGal4. *Red* bars describe the RM annotations and *green* bars TRF annotations. The three samples on the right describe the average coverages in chromosomes and linkage groups (Assembled), in the sum of the unassembled scaffolds (Unassembled) and in the complete galGal4 model. **b** Coverage of each type of SSR in the galGal4 chromosomes. The coverages of simple repeats corresponding to polyA (in *red*) and polyC (*dark blue*) stretches are at the bottom of each bar, microsatellites (*yellow*) are just above them, minisatellites (light blue) are above them, and uppermost are the two types of tandem arrays, large tandem arrays (*orange*) and satellite DNAs (*green*)

The proportion of SSRs in the galGal4 model has been estimated, using RM, to be 1.73 % [61]. We reinvestigated this number by examining the diversity and number of microsatellites using the FASTA program of the GCG computer package [8] and *sputnik* [9] while those of satellite DNAs were investigated using a variety of molecular approaches (for a review see [44]). Using TRF, which can detect SSRs with repeated units from 1 bp to 2 kbp, we found that the assembled genome contained 3.73 % SSRs and the unassembled genome contained 12.74 % SSRs, for a total coverage of 4.08 % in the galGal4 model. These proportions are at least twice as large as those found with RM (2.36-folds with rates varying from 1.11 to 9.13-folds, depending on the chromosome; Table 2 and Fig. 3a). We then went on to look at the features of each type of SSRs. We identified 4 SSR types based on the complexity of their repeated unit sequence: simple repeats, microsatellites, minisatellites, and tandem arrays with repeated units of 60 bp. to 2 kbp. long that were selected when they were composed of at least 2 repeats. We divided these large tandem arrays into two categories: large tandem repeats (<50 repeated units) and satellite DNAs (>50 repeated units). The coverage of the various types of SSRs in chromosomes indicated that the overall densities of simple repeats and microsatellites were similar. In contrast, minisatellites and tandem arrays were more abundant in some of the galGal4 chromosomes (16, 21, 22, 23, 25, 26, 27, 28, LGE22, and LGE64) and more similar in others (Fig. 3b). The proportions of the various categories of SSRs are summarized in Table 3 and their features are shown in Additional file 3.

**Table 2:** Percentages of SSRs found using ISB annotation or TRF in the Galgal4 model

Sequence type	RM	TRF	Increase factor
Assembled in chromosomes	1.54	3.73	2.42
Unassembled	5.67	12.24	2.16
Total in Galgal4	1.73	4.08	2.36

**Table 3:** Number and diversity of simple sequences repeats (SSRs) in Galgal4

SSRs type		Number of arrays	Number of different repeated units^a^	% coverage in galGal4
Simple Repeat (stretches of A or T, and C or G)^b^		204434	2	PolyA : 0.355 PolyC : 0.022
Microsatellite [2–10] bp^b, c^		770202	2101	2.189
Minisatellite [11–60]bp^b, c^		12310	123	1.273
Tandem arrays [>60] bp^d^	Large tandem repeats	6	6	0.003
	Satellite DNAs	10136	9987	0.238

^a^the threshold used to gather two repeated unit is a sequence similarity of 100 %; ^b^the minimal size for an array is 50 repeated units; ^c^between brackets are indicated the size of the repeated unit of each SSR type; ^d^the minimal size for an array is 2 to 50 repeated units of large tandem repeats et 51 to ∞ for a satellite DNA

#### de novo detection and annotation of dispersed repeats (STEP3)

**Fig. 4:**
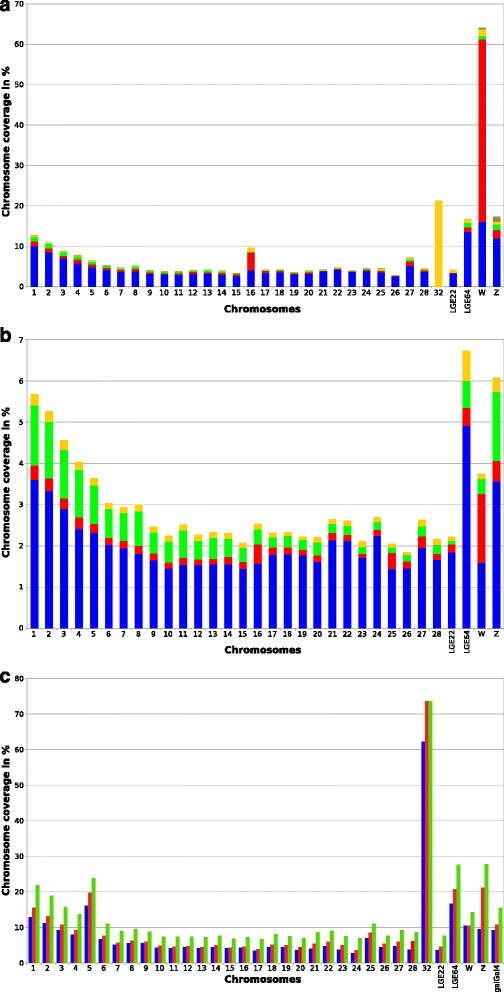
Coverage of TEs and DM annotations in galGal4 chromosomes. **a** Percentage coverage of each chromosome by repeats resulting from the REPET annotation (STEP3, Fig. 2). **b** Percentage coverage of each chromosome by TE segments resulting from the DM annotation (STEP4, Fig. 2). In **a** and **b** *blue* bars indicate the coverage of non-LTR retrotransposons (CR1), *red* bars LTR-retrotransposons and solo LTRs, yellow bars DNA transposons, *green* bars repeats of undetermined origin, and kakhi bars indicate z-reps (a repeat unique for chromosome Z). In **b** chromosome 32 was removed because there was no annotation. **c** Percentage coverage of each chromosome or the complete galGal4 model by the RM annotation (*blue* bars), the REPET annotation (STEP4, Fig. 2; *red* bars) and the sum of TE and DM annotations (STEP3 and 4, Fig. 2; *green* bars)

The REPET pipeline was used to detect repeats and produce annotations. It is composed of two sub-pipelines, TEdenovo that detects repeats using a de novo method based on the repetition of sequences, and TEannot that produces annotations using a combination of programs and post-processes (see Additional file 4, [22–24]). We used an iterative strategy involving three runs of the REPET pipeline to completely annotate galGal4 (Fig. 2) and a version of the galGal4 model from which the SSRs in chromosomes and a 9 Mbp satellite DNA composed of ~22 kbp repeated units in the Z chromosome had been removed [69]. The first run (REPET1; Fig. 2) reported 3926 consensuses (Library 1) corresponding to repeated sequences. These were filtered with TEannot to eliminate residual redundancy between consensuses (i.e. contigs that were identical enough were fused) and those which had no full-length copy in galGal4. The resulting 790 consensuses (Library 1f) were then used to annotate galGal4 to extract the annotated repeats and calculate a reduced version of the galGal4 model. The second REPET run (REPET2; Fig. 2) was run using the reduced galGal4 model and produced 186 consensuses (Library 2). These were filtered and 133 new consensuses were selected (Library 2f). Libraries 1f and 2f were merged in step 3 of REPET (Fig. 2), and filtered manually to remove redundant sequences as well as sequences corresponding to tandem repeats and segmental duplications (Library 3; 613 consensuses). Finally, these libraries were filtered using TEannot and resulted in 581 consensuses that were reduced to 499 (Library 3f) by manual curation to eliminate consensuses corresponding to pseudogenes. The final annotation of galGal4 was calculated with Library 3f using TEannot and revealed a TE coverage of 11.524 % (Fig. 4a).

#### Detection and annotation of highly divergent repeats, mining the dark matter (DM; STEP4)

Genomic dark matter may be defined as “all intergenic sequences, irrespective of functionality or expression” [70–72]. Scientific interest in dark matter was triggered by the discovery of non-coding RNAs (ncRNAs) that could regulate gene expression. Several reports have shown that dark matter is a source of ncRNA and that it can cause disease when it malfunctions [73–75]. Today’s studies on dark matter are designed to annotate non-coding RNAs using RNA-Seq, cDNA sequencing, tilling arrays or to annotate cis-regulatory DNA elements using Dnase-seq [72, 76]. Because genomes have undergone bursts of TE production during their evolution and because these TEs are actively repressed [77], dark matter could also be considered as a graveyard containing very different, recombined TE copies. Repeats with sequences that are well conserved can be annotated using default values in the REPET pipeline. We used a library containing all repeated copies of the REPET annotation and the TEannot program to access the DM, the older and/or fragmented TE segments (Fig. 2, STEP 4). Our aim was to use a population of genomic copies as a probe to detect more divergent repeats (see Methods). Computational constraints obliged us to select only TE copies >500 bp (33,757 copies). The 33,757 copies used at this step each originated from one of 222 consensuses calculated by REPET. Annotation of the DM increased the TE coverage in the galGal4 model to 4.7 % (Fig. 4b).

#### Finishing the repeat annotation

A characteristic of TEannot output files is that each TE copy (i.e. all TEs corresponding to complete elements, internally deleted elements, 5' or 3' truncated elements and elements truncated at both ends) can be split into several annotations linked to different consensuses belonging to a single TE model. We prepared an inventory of TE copies in galGal4 by processing the final annotation with GFFtools to resolve and merge stacked (i.e. TEs copies with several consensuses used for their annotation) and juxtaposed annotations. The minimal TE copy size was set at 20 bp, 4-bp larger than that of the oligo used as a motif to study repeats in Red and P-clouds and 10-bp larger than that used in the ISB annotation.

**Fig. 5:**
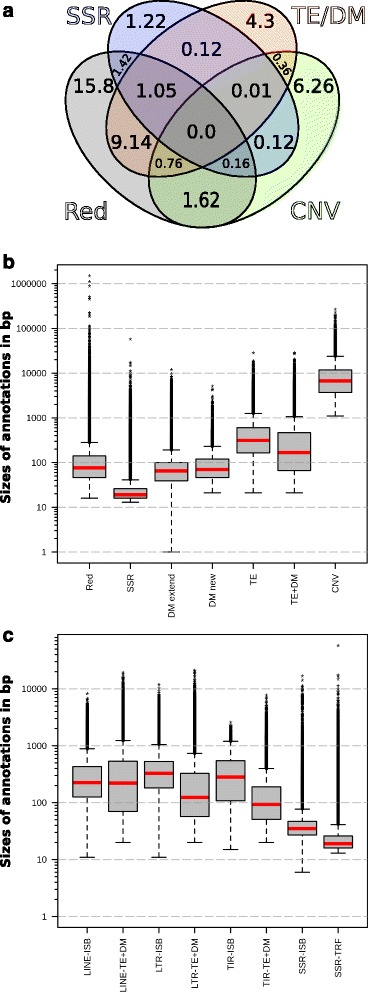
Features of annotations calculated by Red, REPET, and RM. **a** Venn diagram showing the overlaps between the annotation files calculated with Red (RED), TRF (SSR), and REPET (TE + DM), and CNVs [11]. Values correspond to coverage percentages in galGal4. **b** Distributions of annotations sizes calculated with Red, TRF (SSR) and REPET (DM, TE and TE + DM), and those of the CNVs [11]. DM annotations were split into two batches corresponding to DM annotations that extend pre-existing annotations produced with the same TE model (DM extended) and those that are new (DM new). **c** Size distributions of LINE, LTR, TIR and SSR annotations calculated with RM together with those obtained with REPET or TRF for the same categories. Vertical axes in A and B indicate log_10_(sizes) in bp. The *red* lines in the box plot indicate the median value, the ends of *grey* boxes the quartile 1 and 3 values, the ends of whisker the 10^th^ and 91^st^ percentiles of the size distribution, and the black stars the highest and the values above or below the 1.5 interquartile range respectively within 1.5 interquartile range of the highest or the lowest quartile

Post processing the DM increased TE coverage by ~45 %, with the [TE + DM] annotation covering 15.7 % of galGal4 (Fig. 4c, Additional file 5). This proportion of TE coverage may be compared to the 9.74 % coverage in the ISB TE annotation [61]. Almost all (99.7 %) DM annotations (4.41 % of coverage in galGal4) were new and only 0.3 % of them extended existing REPET annotation (0.035 % coverage in galGal4). The sum of SSR and [TE + DM] coverages suggests that there are at least 19.78 % repeats in galGal4. But this estimate was corrected by intersecting SSRs and [TE + DM] annotations using bedtools software (Fig. 5a). Because [TE + DM] includes 1 % coverage by SSRs, the amount of annotated repeats was 18.78 %, which was 1.64 times more dense than the ISB annotation [61]. Intersections were also calculated with the CNV annotation [68], as were those obtained with Red and P-clouds. These revealed that ~7.9 % of low-repeat sequences (Fig. 5a, 6.26 % + 1.62 %), corresponding to CNVs, could be added to the 18.78 % of repeats, for a total of 26.7 % repeated sequences in galGal4. Looking for the intersection between the Red or P-clouds annotations with other annotations ([TE + DM], SSRs and CNV) led to embarrassing results regarding the ability of these two methods to reliably calculate the total amount of repeats in a eukaryotic genome. We found that 30 % of the [TE + DM] annotations (4.43/15.7 % coverage in galGal4) were not identified by Red and 53 % of the Red annotations (15.8/29.9 % coverage in galGal4) had no counterparts among the [TE + DM], SSRs and CNV annotations (Fig. 5a). These results are even more damning for P-clouds since 72 % of the annotations had no counterparts among the [TE + DM], SSRs and CNV annotations (Additional file 6).

As the fragmentation of annotated copies could lead to artefacts during the TEannot step we investigated the quality of the de novo [TE + DM] annotations. First, we examined the size distribution of annotations resulting from Red for the overall amount of repeated sequences, TRF for the SSRs, REPET for the TEs, TEannot for the DM and [TE + DM] and the CNV (Fig. 5b). This revealed that the range of annotation sizes calculated by Red covered the sum of those of the other 5 categories and that 90 % of the TE copies were 20 bp. to ~1100 bp. The size distributions of annotated copies for each kind of repeat were then compared to those of the ISB annotations (Fig. 5c). The size distributions of LINE annotations were similar to those of the ISB, while those of the LTR, TIR and SSR repeats were smaller. This was expected since DM annotations were derived from more fragmented TE copies. Next, we analysed the diversity of repeats described in the [TE + DM] annotations, their commonalities the ISB annotations and the quality of the annotation. This revealed that the coverage patterns by each TE type of 3 chromosomes (16, 32 and W) were very different from those of other chromosomes (Fig. 4a and b). These different profiles are perhaps due to the small size of the galGal4 model chromosome 32 (1028 bp), to the greater amount of LTR-retrotransposons in chromosomes 16 and W, or perhaps to the non-random distribution profile of some TEs. These and similar issues are discussed below.

### Diversity and Features of TE models in the [TE + DM] annotation

#### Ranking dispersed repeats within a TE “species” or repeat

Each TE or repeat “species” in libraries such as Repbase or that of the ISB is defined by a consensus sequence. This consensus sequence may be thought of as the sequence closest to an averaged sequence from a population of copies originating from a single genome. Potential protein coding capacity may also play a role in defining these consensus sequences. The methods used to calculate these nucleic acid and protein consensus sequences have not been published by the ISB. Because these consensus sequences cannot represent all sequence variation they are of limited value for detecting TEs. Platforms such as Dfam [78, 79] were developed to circumvent this issue by using a library of hidden Markov models that is set up from existing populations of sequenced elements to annotate genomes. Although Dfam improves significantly the sensitivity and takes better account of TE sequence variations, it is still of limited use for detecting the diversity of rearrangements of TEs such as the non-LTR retrotransposons and, to a lesser extent, some LTR-retrotransposons and DNA transposons.

We have borrowed the concept of the TE model developed by the creators of the program RepeatExplorer [80, 81] to describe a “TE species”. This concept is also included in the philosophy of REPET [22, 24]. It assumes that a TE model is composed of a main consensus sequence (the most complete version of the TE) plus all the consensuses detected as variants. Using this concept, our final 3f library contains 499 consensuses distributed among 34 TE models (TEs or repeat “species” listed in Table 4, correspondences between Repbase and ISB consensuses and the 34 TE models are shown in Additional file 7). The final clustering steps were performed manually using information from sequence databases because BLASTclust in TEdenovo does not calculate models that are consistent with the galGal4 sequences. Our 34 TE models were in striking contrast to the ISB annotation [61] that describe 317 different TE consensuses (TEs or repeated “species”, from which 65 consensus sequences corresponding to repeated genes encoding structural RNA - tRNA, U RNA, 5S RNA, rRNA, etc - must be removed). The many Repbase and ISB consensuses corresponding to non-gene repeats (252) was partly due to fragmentation of a significant number of repeats intp several consensus sequences associated with a single TE species. Thus, 21 of our TE models were split into 81 different Repbase and ISB consensuses (Additional file 7). Furthermore, there were 171 Repbase and ISB TE consensuses involved in the ISB annotation [61] that were found in 81,805 annotations covering 2 % of the genome without any equivalent among our 34 TE models. Conversely, 13 of our models had no corresponding sequence in Repbase/ISB TEs.

**Table 4:** Features and diversity of TE models found in the galGal4 model based on the REPET and DM annotations (STEP3 + STEP4, Fig. 2) after stack resolving and merging stacked and juxtaposed annotations

Names of TE models	a	b	c^a^	d	e
CR1	308	LINE	413857	66.4707	11.8457
Ancestral_LTR_group_1	3	LTR	86	0.0138	0.0034
Ancestral_LTR_group_2	1	LTR	22	0.0035	0.0013
Ancestral_LTR_group_3	1	LTR	40	0.0064	0.0012
Ancestral_LTR_group_4	1	LTR	308	0.0495	0.0119
BIRDDAWG	10	LTR	6238	1.0019	0.2525
EAV	1	LTR	191	0.0307	0.0212
EAV-HP	7	LTR	765	0.1229	0.0496
ERV2	2	LTR	426	0.0684	0.0209
ERV7	10	LTR	2885	0.4634	0.1061
ERV11	1	LTR	512	0.0822	0.0168
Kronos	46	LTR	30732	4.9359	0.7377
putative_LTR_group4	2	LTR	835	0.1341	0.0137
putative_LTR_group9	1	LTR	170	0.0273	0.0017
putative_LTR_group12	17	LTR	1797	0.2886	0.05
putative_LTR_group22	3	LTR	1219	0.1958	0.0257
putative_LTR_group28	2	LTR	367	0.0589	0.0116
putative_LTR_group30	13	LTR	3847	0.6179	0.0996
retroCalimero	1	LTR	826	0.1327	0.0540
retroSaturnin	1	LTR	161	0.0259	0.0118
retroTux	2	LTR	2490	0.3999	0.1243
Soprano	19	LTR	3014	0.4841	0.1171
Charlie	3	TIR	37319	5.9939	0.5868
Charlie-Galluhop	5	TIR	67691	10.872	1.0296
Galluhop	2	TIR	4588	0.7369	0.1198
Mariner1_GG	10	TIR	5686	0.9132	0.1491
Hitchcock	4	undefined	27033	4.3418	0.4182
undetermined_group_1	3	undefined	2219	0.3564	0.0773
undetermined_group_2	2	undefined	1030	0.1654	0.0165
undetermined_group_3	2	undefined	174	0.0279	0.0045
undetermined_group_4	4	undefined	2550	0.4096	0.0423
undetermined_group_5	2	undefined	134	0.0215	0.0036
undetermined_group_6	1	undefined	372	0.0597	0.0100
Z_rep	9	undefined	3032	0.487	0.1476
Total	499		622616	100	16.1832^b^

a, Number of consensus; b, TE types; c, Total number of TE copies; d, Percentage of the total number of TE copies; e, Percentage of chromosome coverage; ^a^Post stack resolving and annotation merging are called copies all complete elements, internally deleted elements; 5' or 3' truncated elements and elements truncated at both ends (i.e. internal regions of a TE devoid of ends). ^b^this coverage value was more elevated than the 15.7 % indicated in the main text because the coverage corresponding to the small TE copies nested in larger TEs were not removed for these calculations

#### TE models in the [TE + DM] annotation

Our results confirmed those of previous studies [60, 61] that showed that there were three main types of TEs in the galGal4 model genome with very different coverage values (Fig. 4a and b): non-LTR retrotransposons (LINEs; 1 TE model), LTR retrotransposons (LTR; 21 TE models), and DNA transposons (TIR; 4 TE models).

The galGal4 model contained a single "species" of non-LTR retrotransposon, CR1. These were the most abundant TEs with 413,857 copies representing 66.47 % of the [TE + DM] annotation (Table 4, Fig. 4a and b). In the light of the above analysis, we re-investigated their diversity and found 8 sub-families (Additional file 8).

**Fig. 6:**
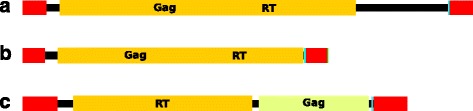
Sequence organization of retroCalimero (**a**), retroSaturnin (**b**) and retroTux (**c**). *Red* boxes indicate 361-bp LTRs in retroCalimero (6837-bp), 334-bp LTRs in retroSaturnin (4624-bp) and 498-bp LTRs in retroTux (5800-bp). *Cyan* boxes indicated polypurine tracts just upstream of the 3' LTR. *Yellow* boxes indicate regions of interrupted coding frames for Gag or RT detected on the sense strand. The *green* boxe in C locates a coding frame for Gag on the anti-sense strand. We found interrupted frames encoding an RT on the sense strand and a Gag-like protein (so-called natural cytotoxicity triggering receptor 3 ligand 1 precursor among blastx hits obtained with the nucleic acid database at the NCBI website) on the anti-sense strand in the inner regions of retroTux. Nucleic acid sequences are shown in Additional file 9

Copy numbers of the 33 other models of TEs and repeats varied from 22 to 67,691 and together represented 33.53 % of the REPET annotation. Twenty one “species” of LTR-retrotransposons were found in the REPET annotation of galGal4 (Table 4). These were present as copies with two LTRs or solo LTRs resulting from the loss of the inner part of the LTR retrotransposon by recombination between the LTRs of each inserted element (Table 4) [82], or both forms. We found no copies corresponding to complete, internally deleted, or partly truncated element of six models of solo LTRs (putative_LTR_group 4, 9, 12, 22, 28 and 30). But the REPET annotation identified new LTR-retrotransposon “species”. This included the retroCalimero, retroSaturnin and retroTux (Fig. 6 and Additional file 9), and 4 species of old LTR-retrotransposons (Ancestral_LTR_group1 to 4; Table 4) of which only large internal fragments with damaged frames coding for the Gag, RT and/or Env proteins remain in the galGal4 chromosomes. We retained the division into four TE models as previously proposed for DNA transposons [60] (Table 4), keeping in mind that they originated from only two species of DNA transposons. Galluhop was an internally deleted form of Mariner1_GG, and Charlie-Galluhop resulted from the insertion of one Galluhop element into a Charlie element before amplification of this chimeric element by a Charlie-mediated transposition within chromosomes. We also found 27 consensuses within 8 TE models (Table 4; Additional file 10) whose sequence features did not match those of one of the three types described above or with any other known eukaryotic TE [12].

These 34 TE models were completed manually using published data [83, 84]. This identified four more TE “species” whose low copy number in galGal4 made them undetectable using other annotation strategies (Fig. 2). Two LTR elements, the Rous sarcoma virus and the Avian myelocytomatosis virus, were integrated as single complete copies into chromosome 1. We also found several repeated segments corresponding to an inner region of the Rous sarcoma virus genome in chromosome 20. Three ancient LTR-retrotransposons appear to have become domesticated in neogenes; these were found near the origin of the *ENS1*, *2* and *3* genes [85], the *OVEX1* gene [86] and the *map1-like* gene (Accession Number: XP_003641886.2) on chromosomes 2, 15 and 10, 14 and 10, respectively. We also found remnant copies of DNA transposons, such as a *Polinton* TE [12, 87] on chromosomes 2 and Z. These remnant sequences still contained interrupted frames coding for an RVE integrase and a *Megaviridae*-like major capsid protein on chromosome 2, and DNA polymerases B on chromosome 2 and Z (the best conserved was on the Z chromosomes). These regions are conserved in chromosomes 2 and Z of the *Meleagris gallopavo* (turkey) genome; the coding frames for the DNA polymerase B on the Z chromosome are the easiest to elucidate (Additional file 11). There were also traces of a wide variety DNA transposons within 27 neogenes coding for transposase derived proteins, all of which must have emerged before the evolutionary separation of the mammalia and sauropsida lineages (Additional file 12).

#### Differences between ISB and [TE + DM] annotations

As indicated above, the results of our [TE + DM] and ISB annotations were not in complete agreement (Additional file 13). 171 Repbase and ISB TE consensuses involved in the ISB annotation had no equivalent in our TE models. We investigated these differences to compare how the two methods annotated loci, followed by determining the quality of the ISB annotations that had no annotation by our procedure (see Additional file 14). The main conclusion was that the annotations calculated with library-based methods depend heavily on the quality of the library used. A library that is not composed of well-curated consensuses tends to force and fragment annotations.

### Re-discovering the distribution profiles of TEs in galGal4 chromosomes

#### TE distributions among functional elements in galGal4

**Fig. 7:**
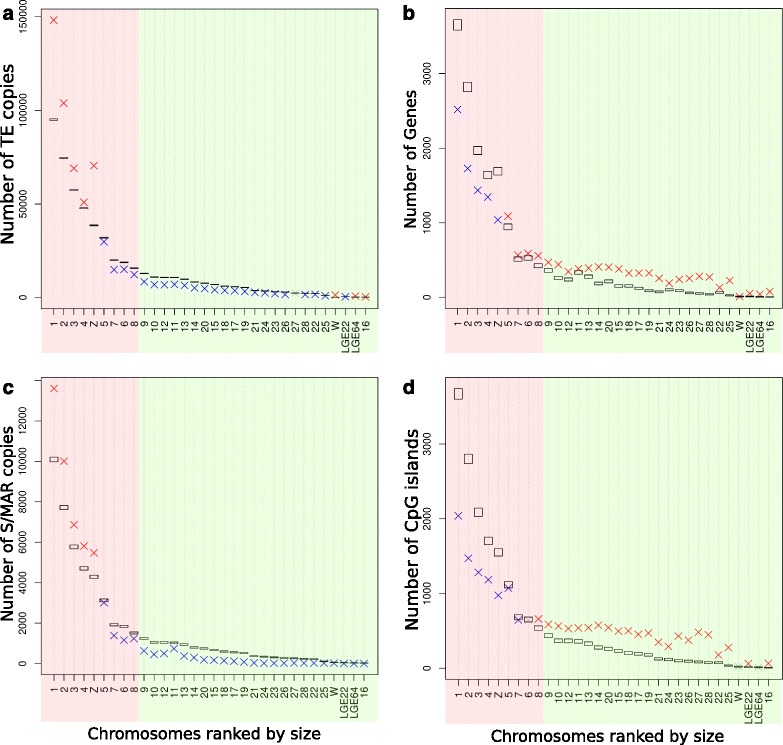
Graph showing the expected and observed numbers of copies of TEs (**a**), genes (**b**), S/MAR (**c**) and CpG islands (**d**) in galGal 4 chromosomes. Each box was calculated from 100000 permutations and represents the 98 % distributions obtained per chance. *Red* crosses above the boxes indicate over-representation of the element in the chromosome and *blue* crosses under-representation (*p* > 99 % in both cases). *Pink* backgound areas indicate macrochromosomes and *green* areas indicate microchromosomes. All galGal4 chromosomes were analysed except chromosome 32, which was too small (1028 bases)

The current view of the chromosome organization with respect to TEs [28, 32] is that macrochromosomes display protein-coding genes and TE densities that are respectively lower and higher than those of the microchromosomes. In an attempt to verify these features we investigate the depletions or the over-representations of TEs, genes, scaffold/matrix attachment region (S/MAR) elements and CpG islands in macrochromosomes and microchromosomes using permutation tests (see Methods). The analyses were conducted in terms of numbers of copies (Fig. 7) or coverage (Additional file 15), both of which produced similar results. We then used these 4 DNA elements together with chromosome size to show that there were not two, but at least three types of chromosomes that had at least four features. The first group was composed of chromosomes 1, 2, 3, 4, and Z, the largest chromosomes, with more TEs and S/MARs (Fig. 7a and c) and fewer protein-coding genes and CpG islands than expected by chance (Fig. 7b and d). The second group included chromosomes 5, 6, 7, 8 and 9, with fewer TEs and CpG islands and more protein-coding genes than expected (Fig. 7a, b and c), but with a number of S/MAR elements that varied significantly from one chromosomes to another (Fig. 7d). The third group contained all the smallest chromosomes, these were poorer in TEs and S/MARs (Fig. 7a and c), but richer in protein-coding genes and CpG islands than expected (Fig. 7b and d). Chromosomes W and LGE64 were two notable exceptions that did not fit into these 3 chromosome types. They had features of both macrochromosomes, rich in TEs and CpG islands, and microchromosomes, chromosome size, number of protein-coding genes, and SMARs (Fig. 7, Additional file 15).

**Fig. 8:**
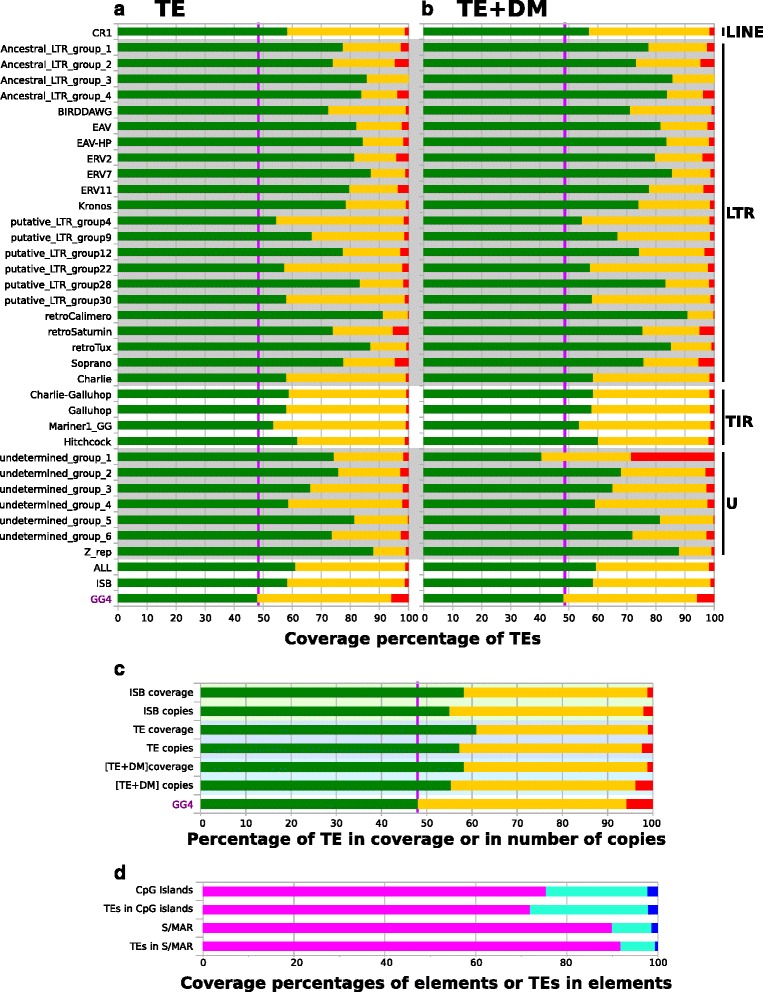
Coverages of TEs in galGal4 chromosomes with respect to the numbers of genes (**a**, **b** and **c**), CpG islands and S/MAR (**d**). Histograms in **a** and **b** show the coverages of TE copies annotated by REPET and those of the [TE + DM] annotation in each chromosome. The names of each of the 34 models are indicated in the left margin. The 3 bars near the abscissa describe data for the 34 TE models (all), those of the ISB annotations (ISB), and the proportions of the exonic, genic and intergenic regions in galGal4 (GG4). A *grey* background indicates one of the four TE types in galGal4: LINE, LTR, TIR and U (undetermined). The name is shown in the right margin. Histogram in **b** shows the proportions of TEs (percent coverage or number of copies). Background areas in *green* indicate TE data from the ISB annotation, *light purple* indicates the REPET (TE) annotations, and *blue* indicates the [TE + DM] annotations. The bar (GG4) near the abscissa shows the proportions of exons, introns and intergenes in galGal4. In **a**, **b** and **c**, the exons (non-coding and coding) are shown in red, genes are in yellow and the intergene regions are in green. A *purple* vertical bar indicates the size of the intergene regions in galGal4. The histogram in **d** represents the coverage/percentages of CpG islands, S/MAR elements and TEs inserted in CpG islands and S/MAR elements (*blue*), the 3-kbp distal and 3-kbp proximal ends of CpG islands and S/MAR elements (*green*) and in the rest of the chromosomes (*purple*)

These features of the RJF chromosome organization were then used to investigate the distribution of TEs with reference to protein-coding genes (Fig. 8a, b, c), CpG islands and S/MARs (Fig. 8d). We checked the TE distribution between exons, other protein-coding genes and intergenic regions in the galGal4 chromosomes using TE annotations resulting from STEP3 (the best-conserved TE copies; Fig. 8b) and the final [TE + DM] annotations (Fig. 8b). Whichever way it was examined (per TE model or all model together (bars labelled "ALL" in Fig. 10a and b)), the general trend was that TEs were more abundant in intergenic regions, The exception were the repeats of the undetermined_group_1, which were abundant in exonic regions once the most divergent copies (DM) were included in the calculation. Both our annotations and those of the ISB found that the abundance of TE copies in exons were similar. The [TE + DM] annotations (3.6 % and 1.7 % in coverage) showed that there were more TE copies (Fig. 8c) in exons than in the ISB annotation (2.1 %) or the TE annotation alone (2.3 %) and 1.1 % for coverage in both. This suggested that the rate at which ancient and more recent TE copies became recently exonized is similar to those reported for mammalian genomes [88–90].

There were 21,663 CpG islands (average size: 645 bp) and 53,115 S/MAR (444 bp) in galGal4. The abundance of TEs in two kinds of elements and their 3 kbp proximal and distal regions (Fig. 8d) were similar to those in the rest of the genome. This is very different from the human and mouse genomes, where regions containing S/MAR are enriched in TEs [91] and CpG islands are enriched in SINEs [92, 93].

We concluded that TEs are more abundant in the intergenic regions of the RJF genome and are no more concentrated in CpG islands and S/MAR than in the rest of the genome. We determined the densities of all TEs. Every TE species chromosomal distributionwas investigated because the data in Fig. 4 indicated that the distribution patterns of some TE species in chromosomes 16, 32 and W were quite specific.

#### TE distributions between and within galGal4 chromosomes

**Fig. 9:**
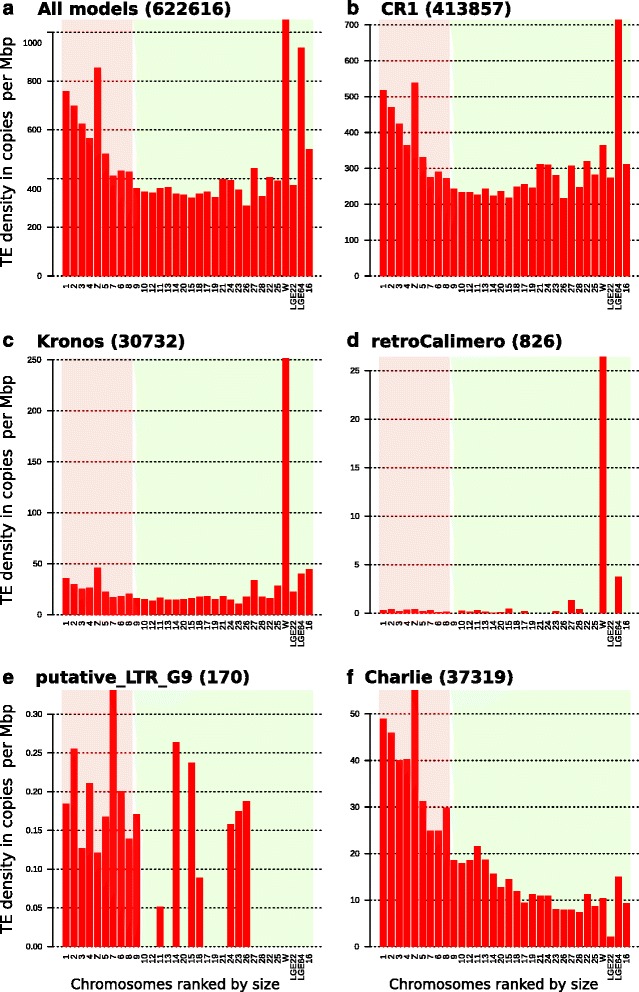
TE density in galGal4 chromosomes. Histograms of TE model densities calculated for all galGal4 chromosomes, except chromosome 32 (too small; 1028 bp). **a**, All TE models, **b**, CR1, **c** Kronos, **d** retroCalimero, **e**, putative_LTR_group 9 and **f**, Charlie. The number of copies for each dataset are indicated in parentheses. Results for all other TE models are shown in Additional files 14 and 15

A survey of global TE density (Fig. 9a) indicated that chromosomes 1, 2, 3, 4, 16, LGE64, Z and W contained more TE copies than the other chromosomes. The profiles of TE species seem to be strikingly different from one species to another. We first found that the global density in CR1 (Fig. 9b) was similar to the global TE density, except in chromosome W. The picture was very similar for each of the 8 CR1 sub-families (Additional file 16). The densities of CR1-C, CR1_F, CR1-G were greater in chromosomes 16 and W than were those of CR1-D, CR1_GG, CR1-Y and CR1_like, which were close to those of chromosomes 5 to 25. The density of CR1-H was elevated only in chromosome W.

We identified six other TE density profiles (Fig. 9 and Additional file 17). The first profile contains CR1s and one other element, Hitchcock (Additional file 17). The TE species in the second and third profiles are found in most chromosomes; they may be super-abundant (Fig. 9f, Additional file 17X to A1; Charlie, Charlie-Galluhop, Galluhop and Mariner, all of them are TIR elements), or less abundant (undetermined_group_1) relative to chromosome size. The fourth density profile included twenty LTRs and five undetermined_group_2 to 6 species; the putative_LTR_group9 is the exception. These were present in many chromosomes at low density, but were abundant in chromosome W and one or more other chromosomes 16, LGE22 and LGE94 (Fig. 9c and d, Additional file 17C to N, P to W, and D1 to H1). The fifth and sixth density profiles each contained just one element, the putative_LTR_G9 (Fig. 9e) is only present in half the chromosomes and Z rep elements are mostly concentrated on the Z and W chromosomes (Additional file 17I1).

**Fig. 10:**
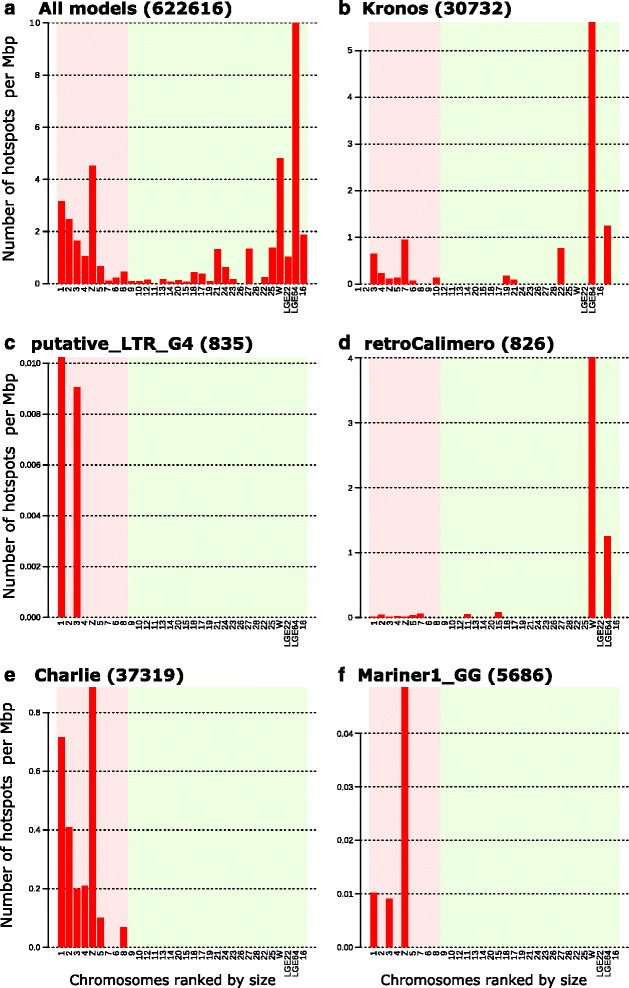
Density of TE hot spots in galGal4 chromosomes. Histograms of TE hot spot density calculated for all galGal4 chromosomes, except chromosome 32 (too small; 1028 bp). Hot spot are defined (*p* > 99 %) using permutation assays with **a** All TE models, **b** Kronos, **c** putative_LTR_group4, **d** retroCalimero, **e** Charli and **f** mariner1_GG. The number of copies for each dataset are indicated in parenthesis. Results for all other TE models are shown in Additional files 16 and 17

We looked for TE hot spots using permutations tests (Fig. 10 and Additional files 16 and 17). Global analysis of all TE models showed that the chromosomes richest in TEs (1, 2, 3, 4, 16, LGE64, Z and W; Fig. 10a) are those that also contain many TE hot spots. The global profile of hot spots for CR1 elements, like the density profiles, is very similar to that of all models (Fig. 10b), except for chromosome W. However, the hot spot profiles for the eight CR1 sub-families were different (Additional file 16). We found that five LTR species had no hot spots in galGal4 (Additional file 17; ancestral_LTR_group4, EAV, putative_LTR_group9, putative_LTR_group28 and undetermined_group_5). This suggests that their distribution is driven only by certain chromosomal features, not by specific regions. The hot spots of other LTR species are generally on chromosome W (Fig. 10b,d, Additional file 17C to W), except for the putative_LTR_group4 (Fig. 10c), whose hot spots were only on chromosomes 1 and 3. The hot spot profiles of the remaining TE species (TIR and undetermined) were all concentrated on the largest chromosomes (Fig. 10e, f), but could be very different from one to another (Additional file 17B1 to I1).

We found that distribution of our 34 TE species along the chromosomes varied between species. Most LTR elements were found on chromosome W, but other than that the distributions of the remaining TE species did not seem to reflect any preferences for insertion in the galGal4 model. Our analysis suggests that most RJF TEs are likely ancient elements that contain significant numbers of point mutations and are thus probably inactive. This in turn suggests that the TE species distributions result both of the insertion preference of each TE species and the ability of the RJF genome to eliminate or conserve them during evolution, depending on the region where each TE is inserted. We cannot examine this topic any further using the chicken recombination maps because these data are not available for the RJF. Calculations from domestic breeds cannot be directly used for the RJF genome since they differ from one breed to another [94], and the extent to which the sizes of the genomes and non-gene regions in between RJF and domesticated lines differ has not yet been evaluated. There is a strong correlation between GC richness and chromosome recombination rates [95], but we find no such correlation between the GC content and local TE densities in chromosomes. The forces driving the density and hot spot profiles of each of the 34 TE models in galGal4 are therefore due to something other than ectopic recombination.

## Conclusions

Our study has succeeded in its two main objectives. First, we have developed a general strategy for annotating (including quality assessment) repeated sequences in a model of an avian genome. Second, we have used this strategy to annotate the repeated sequences in galGal4 using repeat models that can directly be used to annotate the RJF genome.

### Ins and outs of our approach to annotate repeats in eukaryotic genomes

Our study suggests that before investing manpower and resources into genome annotation, researchers would to well to calibrate their annotation strategy using existing information on the size of the real and model genomes, as well as on estimates of repeat amounts. Here, the size of the real genome was estimated from data on several species in various databases [38, 96, 97]. We used a k-mer method to calculate the genome size where data were not available, as was done recently with Cephalopoda species [98]. The reliability of this new approach needs to be tested on both avian and mammal models once the program is available. Reassociation kinetics data are particularly valuable because tools such a P-clouds and Red are unreliable for estimating the total proportion of repeats in galGal4. While implementation of our annotation strategy required a significant investment in time and computer resources, it enabled us to annotate the repeats in galGal4 more reliably than using RM. Our annotation strategy has shown that there are more repeats (~18.8 %, rather than ~11.5 % in the ISB annotation) and less TE diversity (34 rather than over 200 in the ISB annotation) in galGal4 than previously reported [28, 60, 61].

Our results confirm that de novo approaches for annotating repeats are more efficient than library-based method and are less likely to produce artefactual annotations. We found that at least some of the ISB annotations (0.76 % of the 8.87 % of TE annotation in coverage) are probably artefacts. This is a fault shared by all library-based methods, which tends to force the search for sequence matches that vary greatly in size to consensuses present in the reference sequence library. This fault can be amplified when many heterospecific TE sequences are available and the reference library contains no specific repeated sequences. Nevertheless, previously published data and Repbase were useful. Indeed, in our hands REPET was able to produce many consensuses (308/499) that would have been difficult to manage without any idea of their putative organisation in sub-families, thanks to the many described CR1 non-LTR retrotransposons in galGal4, their 5' truncation profiles, and ages. Therefore we suggest that anyone wanting to use a similar annotation strategy on other models shoulf perform preliminary analyses of non-LTR retrotransposons (LINEs and SINEs) before implementing the current version of the REPET pipeline.

The galGal5 genome model was released in January 2016 [99], just as we were preparing the final version of our manuscript. This new version contains 1.232 Gbp, close to the C-value (1.223 ± 0.058 Gbp) and has fewer ambiguities (only 0.95 % “N” in its sequence, compared to 2.39 % in galGal4). Its greater size is due to the discovery of about 6400 new genes (18644 in galGal4, 25062 in galGal5). Repeat annotation with RM revealed that galGal5 has 6.98 % satellite SSRs and 9.06 % TEs, for a total of 16.04 % repeated sequences. These repeated sequences were annotated using the approach and models described above. Results and gff files are available at http://chicken-repeats.inra.fr/. They indicate that are 10.50 % SSRs and 10.86 % TEs; our annotation gives the total amount of these repeated sequences as 21.36 %. We verified the distributions of TEs in the inter-gene and intra-gene regions and found results similar to those presented here in Fig. 8a and c (results are available at http://chicken-repeats.inra.fr/).

### New insights provided by a deeper repeat annotation

In addition to the number of repeats and TE diversity, our annotation update modifies the landscape of repeats in the RJF genome. First, even though further investigations will be required to evaluate their exact sizes [44, 53, 100, 101], the sum of the 4–8 % of centromere and telomere sequences to the 26.7 % of repeats found in galGal4 (SSRs + TEs + CNVs) is not far off from the real RJF genome (31-35 % repeated sequences, half of them TE sequences). Although the RJF genome contains fewer repeats than most mammal genomes, this repeat content is nearly a 3-times greater than previous estimates, similar to the repeats in Chiroptera (bat) genomes [102]. The distributions of repeats in avian chromosomes differ from those in other vertebrate genomes in at least two ways. First, there are many different, small families of satellite DNAs interspersed along chromosome arms in addition to repeats in megacentromeres and megatelomeres, and these satellite DNAs are more abundant in small chromosomes. This distribution of satellite DNAs might in fact label each small chromosome with something like a satellite DNA code. These labels might even be involved in chromosome recognition and influence the physical separation of small and large chromosomes that occurs during cell division in birds [103]. Second, none of the 34 TE species found in galGal4 are randomly distributed along chromosomes. Most of them are arranged in specific patterns that suggest that they were not randomly inserted into chromosomes during evolution, and conversely were not randomly eliminated from chromosomes.

This brings us to the idea that TEs are inactive in present-day chicken. Recent data indicate that few TE species are active in mammals and insects and that some are involved in development and differentiation pathways [104–106]. It would therefore appear that inactive TEs are an avian characteristic, as these pathways are also present in Sauropsida species. Our annotation indicates that there are at least three active TEs in the chicken genome. The first is EAV-HP, an LTR element that was shown recently to have been active in the chicken [107, 108]. The other two are in elements that were until recently considered to be neogenes coding for transposases, THAP9 and PGBD5 (Additional file 11). These two genes are present and active in every vertebrate species and were recently shown to transpose, in *trans*, non-autonomous related TIRs in the human genome [109, 110].

Thus the importance of TEs in avian genomes is far from completely elucidated; the most abundant TE species may well not be the most interesting candidates for studying genome rearrangements during development.

## Methods

### Genome model

galGal4 (Assembly: GCA_000002315.2; http://www.ensembl.org/Gallus_gallus/Info/Annotation) was downloaded from the UCSC website (http://hgdownload.cse.ucsc.edu/downloads.html). galGal5 was downloaded from the NCBI website [99]. The file describing the annotation of CpG islands in galGal4 was also downloaded from the UCSC website. The annotation file describing the S/MAR sequences is available from Genomatix (https://www.genomatix.de/). All studies were done on both the assembled and unassembled genomes. Because our materials were only in silico data supplied by the UCSC, the NCBI and Genomatix, no ethical statement was required to achieve our works.

### P-Clouds

Version 0.9 was download from the web site http://www.evolutionarygenomics.com/ProgramsData/Pclouds/Pclouds.html. P-clouds does not manage 'N' residues correctly in the sequence of genome models; it considers them to be stretches of 'A' nucleotides. The 14 Mbp of 'N' in galGal4 meant that this creates a huge number of k-mer derivatives from A-stretches that are false annotations. We overcame this problem by developing a wrapper for P-clouds that retains the main program but replaces the original pre-processors and post-processors. The wrapper is a Perl script called 4pclouds.pl (P-clouds pre-post- processor) that creates an index to manage the removal of the 'Ns', then restores the scaling of the chromosomes of the model after P-clouds treatment.

P-clouds requires a set of five cut-off parameters to be launched in addition to the genome sequence to be analyzed. Parameter 1, the lower cut-off, is the minimum number of repeats of the oligo in a genome to be integrated in a P-cloud. Parameter 2, the core cut-off, is the minimum number of repeats of the oligo in a genome to be used as a seed for P-clouds. Parameters 3, 4 and 5 are the primary, secondary and tertiary cut-offs that define the smallest number of repeats required for a core oligo to integrate to the outer layer of oligos presenting one, two or three nucleotide mismatches with it. The optimal parameters are defined by six sets of parameters c4(2, 4, 8, 80, 800), c5(2, 5, 10, 100, 1000), c8(2, 8, 16, 160, 1600), c10(2, 10, 20, 200,2000), c100(10, 100, 200, 2000, 20000), c200(20, 200, 400, 4000, 40000). Each parameter set uses a 16-nucleotide oligonucleotide (k-mer) that was calculated using the formula l = log_4_N + 1, where l is the oligo size and N the genome size [63]. The final output of a P-clouds calculation is a bed file.

### Red

The code of Red (in C++) and complementary information were downloaded from html.http://toolsmith.ens.utulsa.edu. Launching the compiled Red provides the genome sequence to be analyzed and an oligo (k-mer) size that is calculated using the same formula as for P-clouds (16 nucleotides). The final output of a red calculation is a bed file.

### Analyses of SRRs

TRF version 4.07b was downloaded from the tandem repeat finder website (http://tandem.bu.edu/trf/trf.download.html). The Match, Mismatch, Delta, PM, PI, Minscore, MaxPeriod parameters were set at 2, 5, 7, 80, 10, 25, and 2000. The -m option was used to obtain a masked genome and the -d option to obtain the data file output. Data file outputs were analysed using a custom written Perl script to determine the type of repeat of each annotation (simple repeat, microsatellite, minisatellite, large tandem repeats (including satellite DNA). Each annotation was then loaded into a MySQL database from which was produced a GFF file describing the features of all SSRs, each with the attribute (ninth column) containing an ID, the type of SSR, the size of the repeat unit, the repeat unit sequence, the tandem array size and the number of copies of the repeat unit. A second custom written Perl script was used to select simple sequences, microsatellites and minisatellites based on an arbitrary minimum size of 50 tandem arrays. Arrays with units over 60-bp composed of at least 2 repeats were selected and ranked in large tandem repeats when the repeated unit was tandemly repeated fewer than 50 times and in satellite DNAs when they were repeated over this threshold.

### Annotations of dispersed repeats with REPET

Dispersed repeats were annotated in three steps using the REPET package version 2.2 (available at https://urgi.versailles.inra.fr/Tools/REPET). For the first run (REPET 1, Fig. 2), SSRs and a macro-satellite present only in the Z chromosome were removed in galGal4 and TEdenovo was used with its default parameters. TEdenovo is a pipeline that combines several programs to optimize the production of an exhaustive list of consensuses. It was run with galGal4 after discarding three programs (Additional file 4). First, the program PILER, because it could not manage the amount of data produced during the analysis of models such as galGal4. Second, LTR_HARVEST because it produced too many false-positive consensuses. LTR_HARVEST identifies a sequence as an LTR retrotransposon as soon as it can locate two large direct repeats close enough to gather them into a pair of LTR flanking a retro-transposed DNA segment. Thus, LTR_HARVEST identified many purely artifactual LTR retrotransposons in galGal4, where copies of non-LTR retrotransposons like CR1 or the DNA transposons like Galluhop are abundant, whatever the parameter set used. Finally, we removed BLASTclust, which intervenes at the end of the TEannot procedure because it produced aberrant clusters of consensuses under our conditions.

The output of TEdenovo, Library 1 (Fig. 2), was used to produce a first annotation of galGal4 using TEannot with its default parameters. Consensuses in Library 1 were then filtered to produce Library 1f using two programs of the REPET package. PostAnalyzeTELib.py produced statistical descriptions of each consensus used to extract the full length fragment consensuses (consensus with at least one full length copy in the genome) using GetSpecificTELibAccordingToAnnotation.py. Library 1f was then used to annotate galGal4 using TEannot with its default parameters. The resulting annotated genome copies were then used to calculate a reduced version of galGal4. The second run (REPET 2; Fig. 2) was designed to detect other repeats fragmented by nested insertion of repeats identified by REPET1. The REPET 2 run was managed by filtration similar to that used in REPET 1 to produce Library 2 f. The third run (REPET 3; Fig. 2) merged libraries 1f and 2f, which was filtered with TEannot to produce Library 3 f. The name and classification supplied by PASTEC (Additional file 4) for each consensus in TEannot were verified and changed manually because we found 15–20 % errors, depending on the TE model. Library 3f was used to edit the final annotation of galGal4.

### DM annotation

TEs (>500 bp) with at least 80 % sequence similarity to their consensuses identified during the REPET procedure were extracted with GFFtools and used to detect and annotate DM. We then used TEannot with its default parameters and these TEs to mine galGal4 to locate more divergent TE segments corresponding to the DM. The resulting DM was subtracted from the annotation file with bedtools (http://bedtools.readthedocs.io/en/latest/content/bedtools-suite.html) so as to remove all repeats identified in steps 2 and 3 of the complete annotation procedure (Fig. 2).

### Analysis of annotation features

Unique or intersecting annotations were computed using bedtools. The shared annotations were obtained with intersectBed and the intervals were removed using subtractBed. Coverage was computed by summing the lengths of intervals and dividing by the genome size. The results were transferred directly to R (https://www.r-project.org/).

We developed GFFtools (available at http://chicken-repeats.inra.fr/index.php?pages/Tools) to analyse the TE distribution and their coverage in galGal4 chromosomes. Existing libraries like Bio::Tools::GFF in Bio::Perl can parse and analyse GFF files but none of them can readily manage the attributes column (ninth column) of a GFF file and perform operations on features such as reducing intervals. GFFtools has two Perl objects that can store the whole GFF file in a data structure, parse features, add annotations, filter features, reduce overlapping features, and deal with overlaps. GFF files were finalized with GFFtools in order to reduce the number of overlapping features, selecting those most similar and identical between annotations, then those with the highest percentage of coverage with their annotating consensuses.

TE densities were analysed by counting the number of TE copies in each chromosome, except for long-join annotations. This was done for all models and then for each model. The REPET long-join analysis involved merging two annotations related to the same TE model and then splitting them into two or more annotations, depending on the presence of one or more inserted TEs.

### Permutation tests for analysing the distribution of a repeat DNA element

We used a custom written Perl script to determine the size of chromosomes minus the coverage of a single kind of DNA element (TE, gene, S/MAR, CpG island) and thus obtain the size of the reduced genome. We next calculated the random distribution of the number of elements in the reduced genome and the number of copies of the element in each chromosome. We then calculated 100000 permutations per chromosome and fed these data into R to draw a histogram of the number of elements. This gave us the two thresholds at which there was a 1 % chance of getting a TE-rich or TE-poor distribution in each chromosome (Additional file 18).

### Permutation tests for analysing the presence of TE hot spots

We used a permutation test for each kind of TE guild analysed (a TE model or a group of TE models) to determine a threshold above which a chromosome region was considered to be a TE hot spot. We first calculated, using a 50 kbp window, 1000 permutations of randomized TE distributions, and then used these distributions to determine the 1 % threshold above which a 50 kbp region in each chromosome could be a hot spot. The window size was used to take into account the coverages of TEs and Ns and so avoid overlap due to TE content and N stretches (Additional file 18).

## Additional files

Additional file 1: Conditions of use for programs P-clouds and Red. An analysis of parameters to use with P-clouds and Red is presented in order to optimize the calculation of the amount of repeats in an eukaryotic genome. (ODT 36 kb) Additional file 2: Evaluating the efficiency of P-clouds and Red. This file describes data on the relaibility of P-clouds, Red and REPET for evaluating the numbers of repeats in a eukaryotic genome. A synthesis of results supports that the sequence diversity and distribution of minisatellites as well as the lack of repeated satellite DNAs in numerous microchromosomes might be a signature specific to avian genomes. (ODT 23 kb) Additional file 3: Features of SSRs in galGal4. An inventory of simples repeats, microsatellites, large tandem arrays in galGal4 is supplied. (ODT 251 kb) Additional file 4: Diagram showing programs in both REPET TEdenovo and TEannot components. The programs below were run either successively or in parallel from top to bottom as indicated by the red triangles. The three programs in TEannot with blue or pink backgrounds were removed from our analyses and the outputs of the program in yellow were verified by hand. Note: RepeatMasker is part of the TEannot component. (ODT 73 kb) Additional file 5: TE coverage in each galGal4 chromosomes in the ISB, REPET TE, TEannot DM and TE + DM annotations. Results are summarized within a table. (ODS 23 kb) Additional file 6: Intersections between annotation files calculated with P-clouds, TRF, and REPET. Venn diagram describing the overlaps between the annotation files calculated with P-clouds, TRF (SSR), REPET (TE + DM), and CNVs [11]. Values are percentage of coverage in galGal4. (ODT 56 kb) Additional file 7: Correspondence between the names of consensus describing TEs in Repbase and ISB, and the TE models calculated with REPET. Results are summarized within a table. (ODS 27 kb) Additional file 8: Diversity of CR1 within galGal4. The clustering of CR1 copies into subfamilies was re-investigated using SiLiX. Eight subfamilies were found, 7 of them matching with the Repbase sub-families CR1-C, CR1-D, CR1_F, CR1-G, CR1_GG, CR1-H, and CR1-Y. Their respective abundance in galGal4 was summarized in Table S2. (ODT 30 kb) Additional file 9: Nucleic acid sequences of retroCalimero, retroSaturnin and retroTux. The consensus sequences of these three LTR elements were supplied. (ODT 37 kb) Additional file 10: Features of 8 repeat models that cannot be assigned to a known eukaryotic TE. The sequence features of 27 consensuses gathered into 8 TE models were analysed. Results supported that none of them can be definitively considered as originating from LTR elements. (ODT 91 kb) Additional file 11: Proteins encoded by the remnant polinton in the RFJ and turkey genomes. (ODT 23 kb) Additional file 12: Characteristics of the 54 neogenes derived from DNA transposons in the human and RJF genomes. A table summarize the features of 54 genes derived from DNA transposons in the human and RJF genomes. (ODT 32 kb) Additional file 13: The number of RM annotations that had no equivalent in the [TE + DM] annotation. Results are summarized within a table between both approaches. (ODS 36 kb) Additional file 14: Origins of differences between ISB and [TE + DM] annotations. ISB and [TE + DM] annotations were samples in order to define the origins of differences. (ODT 101 kb) Additional file 15: Graph showing the expected and observed coverages of TEs (A), genes (B), S/MAR (C) and CpG islands (D) in galGal4 chromosomes. Permutation tests were used in order to define whether TEs, genes, S/MAR and CpG islands were randomly distributed along chromosomes. (ODT 1829 kb) Additional file 16: Histograms showing the densities of TEs and TE hot spots in galGal4 chromosomes for the 8 sub-families of CR1 elements. Histograms of TE model density and TE hot spot density were calculated for all galGal4 chromosomes, except chromosome 32 (too small; 1028 bp). (PDF 532 kb) Additional file 17: Histograms showing the densities of TEs (left column) and TE hot spots in galGal4 chromosomes for all TEs plus each of the 34 TE models. Histograms of TE model density and TE hot spot density were calculated for all galGal4 chromosomes, except chromosome 32 (1028 bp). (PDF 2152 kb) Additional file 18: Graph showing thresholds calculated in permutation assays and windows calculated along chromosomes for permutation tests designed to inventory hot spots. Graphic representations about how were calculated thresholds in permutation assays and windows along chromosomes. (ODT 526 kb)

## Abbreviations

CNV: Copy number variation
DM: Dark matter
Env: Retroviral envelope protein
Gag: Group specific antigen
ISB: Institute for systems biology
LINE: Long interspersed element
LTR: Long terminal repeats
RJF: Red jungle fowl
RM: RepeatMasker
rRNA: ribosomal RNA
RT: Reverse transcriptase
S/MAR: Scaffold/matrix attachment region
SINE: Short interspersed element
SSR: Simple sequence repeats
TE: Transposable element
TIR: Terminal inverted repeats
TRF: Tandem repeat finder
