# Supplementary material for: Deep landscape update of dispersed and tandem repeats in the genome model of the red jungle fowl, Gallus gallus, using a series of de novo investigating tools
Source: BMC Genomics. 2016 Aug 19;17:659. doi: 10.1186/s12864-016-3015-5 (PMC4992247; doi:10.1186/s12864-016-3015-5)
Supplement: Additional file 16: — Histograms showing the densities of TEs and TE hot spots in galGal4 chromosomes for the 8 sub-families of CR1 elements. Histograms of TE model density and TE hot spot density were calculated for all galGal4 chromosomes, except chromosome 32 (too small; 1028 bp). (PDF 532 kb) [file 12864_2016_3015_MOESM16_ESM.pdf]

Additional File 16: Histograms showing the densities of TEs and TE hot spots in galGal4 chromosomes for the 8 sub-families of CR1 elements. Histograms of TE model density (left column) and TE hot spot density (right column) were calculated for all galGal4 chromosomes, except chromosome 32 (too small; 1028 bp). The number of copies for each dataset are indicated in parentheses.

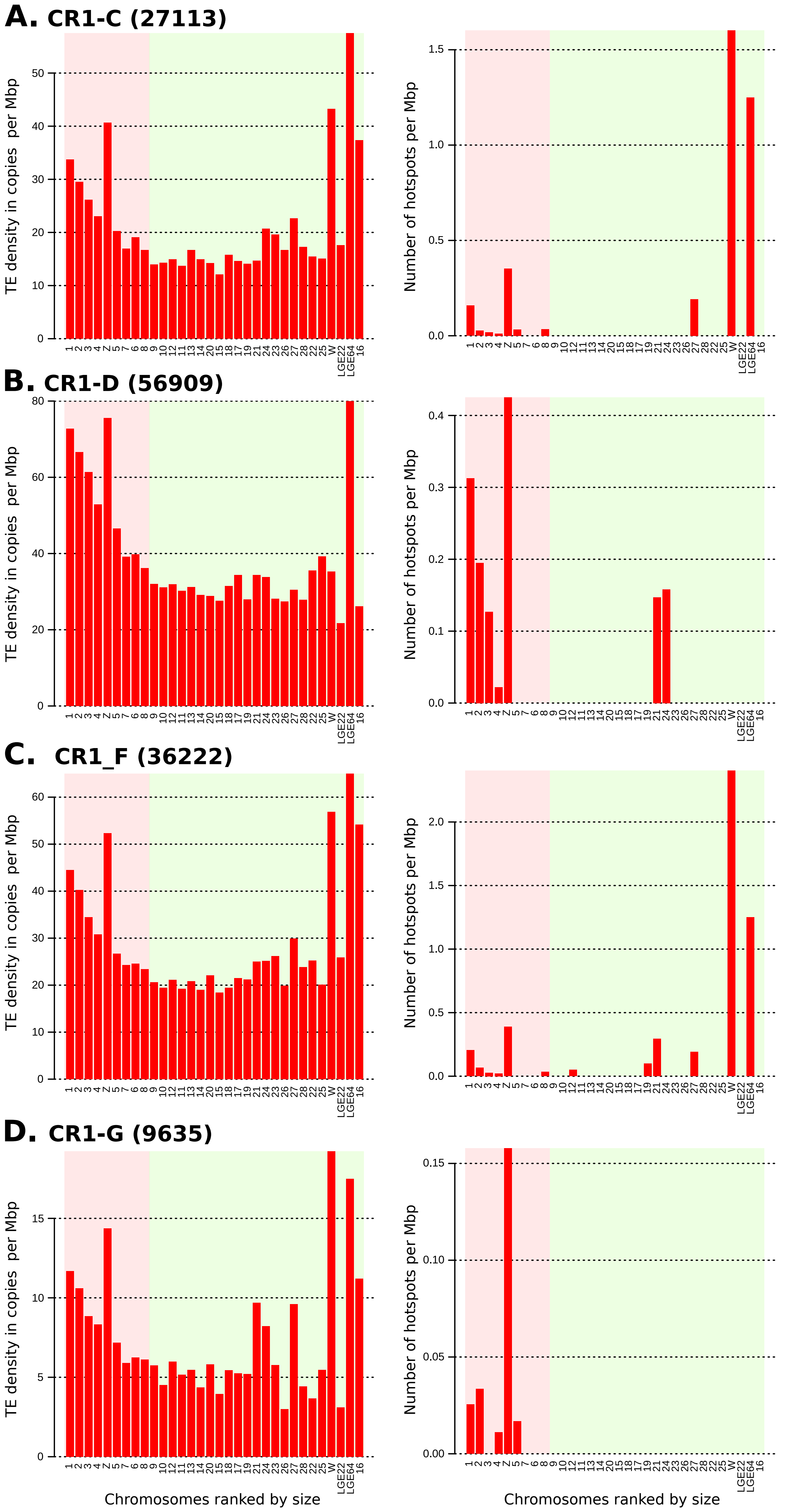

A. CR1-C (27113)
B. CR1-D (56909)
C. CR1_F (36222)
D. CR1-G (9635)
TE density in copies per Mbp
Number of hotspots per Mbp
Chromosomes ranked by size
1 2 3 4 Z 5 7 6 8 9 10 12 11 13 14 20 15 18 17 19 21 24 23 26 27 28 22 25 W LGE22 LGE64 16

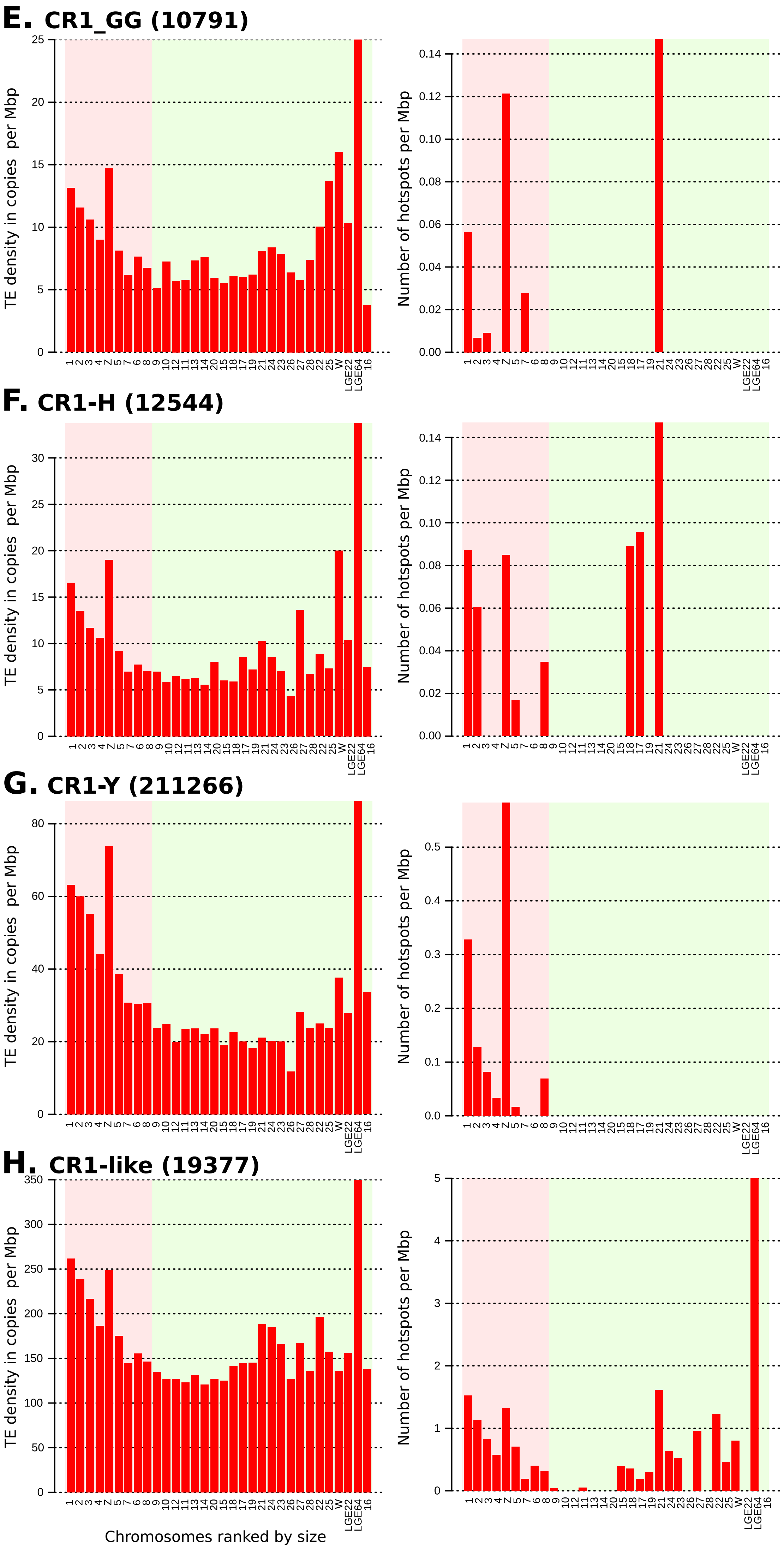
E. CR1_GG (10791)
F. CR1-H (12544)
G. CR1-Y (211266)
H. CR1-like (19377)
TE density in copies per Mbp
Number of hotspots per Mbp
Chromosomes ranked by size
