# Supplementary material for: Deep landscape update of dispersed and tandem repeats in the genome model of the red jungle fowl, Gallus gallus, using a series of de novo investigating tools
Source: BMC Genomics. 2016 Aug 19;17:659. doi: 10.1186/s12864-016-3015-5 (PMC4992247; doi:10.1186/s12864-016-3015-5)

**Additional File 17: Histograms showing the densities of TEs (left column) and TE hot spots in galGal4 chromosomes for all TEs plus each of the 34 TE models.** Histograms of TE model density (left column) and TE hot spot density (right column) were calculated for all galGal4 chromosomes, except chromosome 32 (1028 bp). The number of copies for each dataset are indicated in brackets.

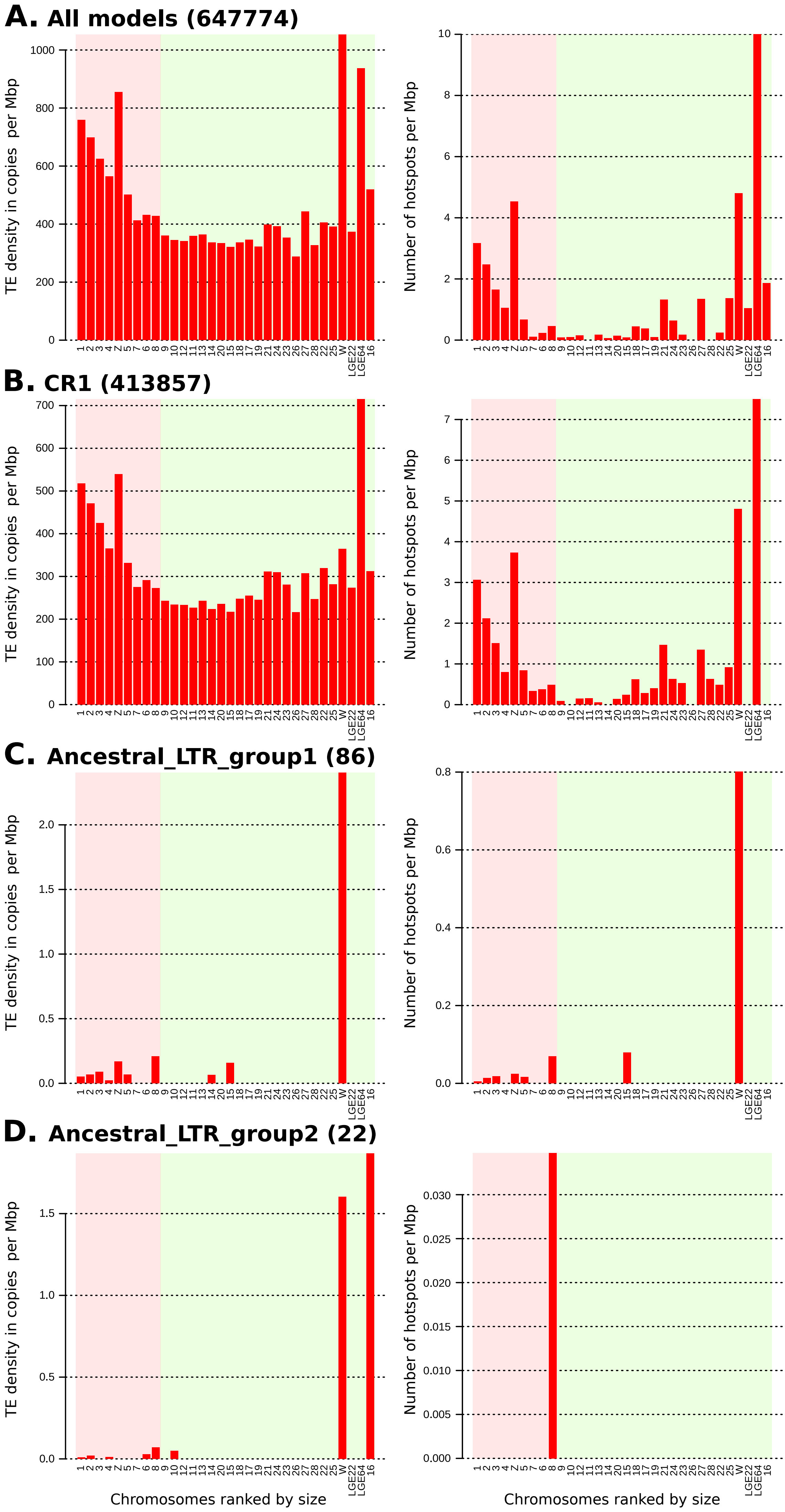
A. All models (647774)
B. CR1 (413857)
C. Ancestral_LTR_group1 (86)
D. Ancestral_LTR_group2 (22)
TE density in copies per Mbp
Number of hotspots per Mbp
Chromosomes ranked by size
1 2 3 4 Z 5 7 6 8 9 10 12 11 13 14 20 15 18 17 19 21 24 23 26 27 28 22 25 W LGE22 LGE64 16

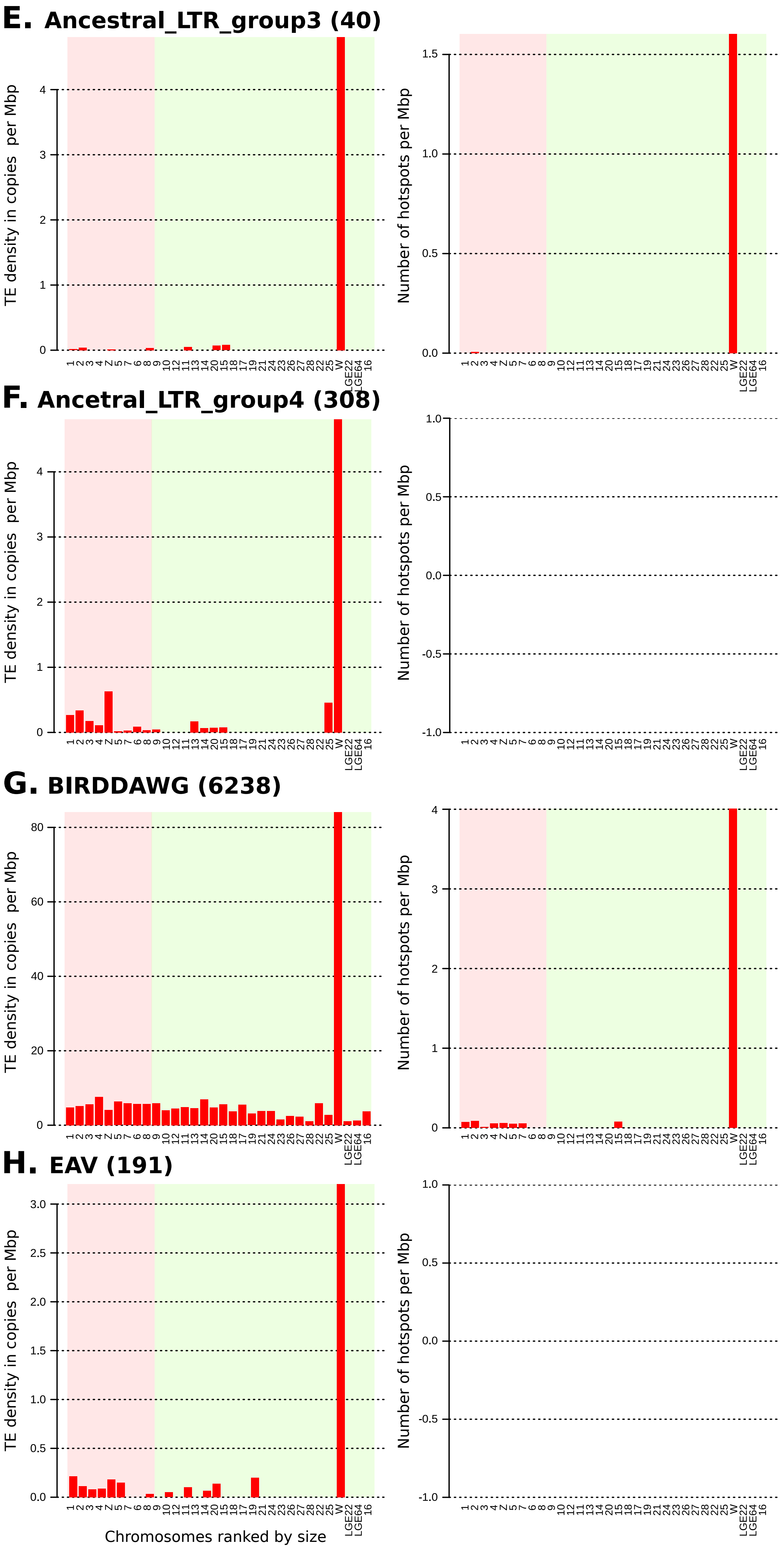
E. Ancestral_LTR_group3 (40)
F. Ancetral_LTR_group4 (308)
G. BIRDDAWG (6238)
H. EAV (191)
TE density in copies per Mbp
Number of hotspots per Mbp
Chromosomes ranked by size
1 2 3 4 Z 5 7 6 8 9 10 12 11 13 14 20 15 18 17 19 21 24 23 26 27 28 22 25 W LGE22 LGE64 16

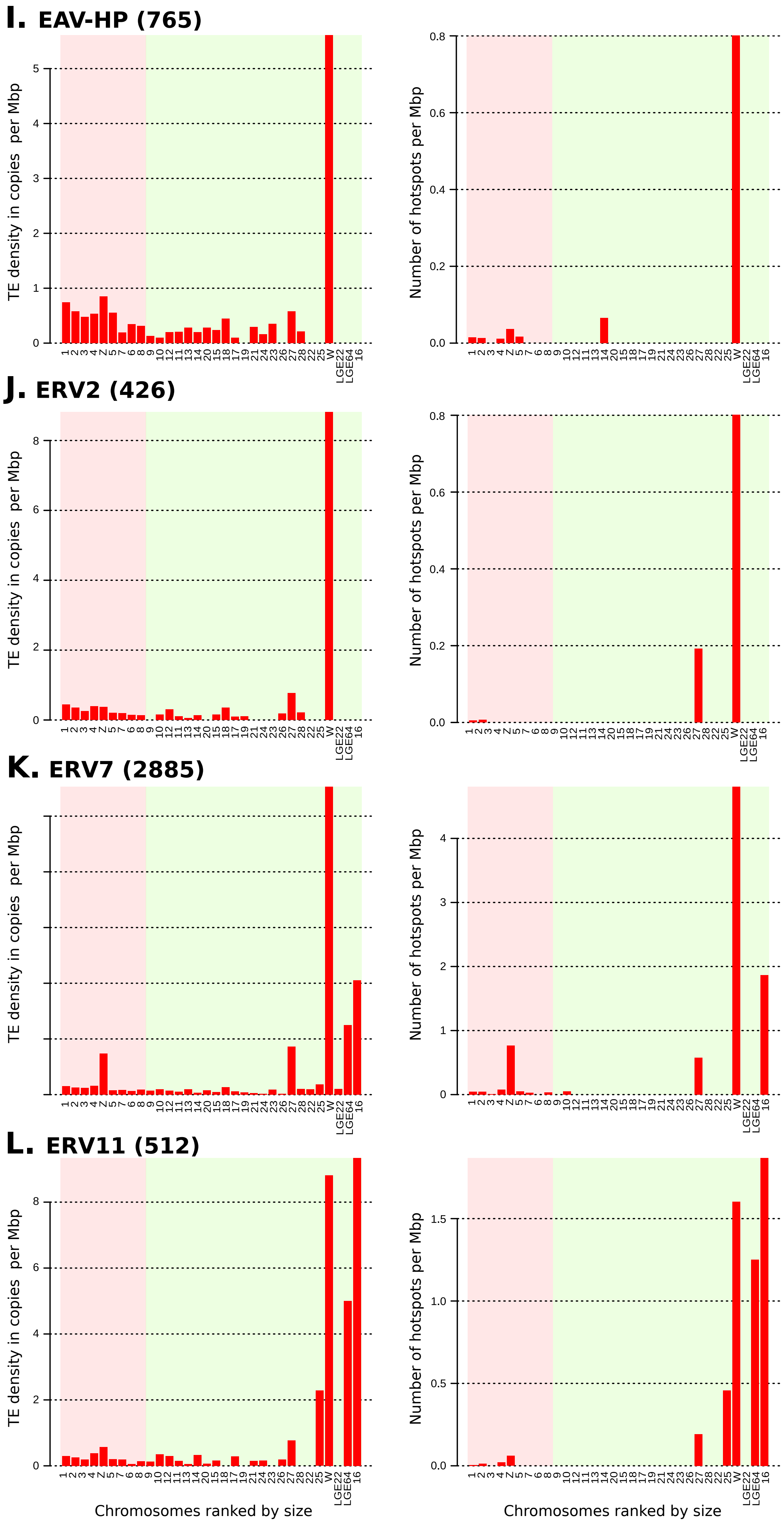
I. EAV-HP (765)
J. ERV2 (426)
K. ERV7 (2885)
L. ERV11 (512)
TE density in copies per Mbp
Number of hotspots per Mbp
Chromosomes ranked by size
1 2 3 4 Z 5 7 6 8 9 10 12 11 13 14 20 15 18 17 19 21 24 23 26 27 28 22 25 W LGE22 LGE64 16

**M. Kronos (30732)**

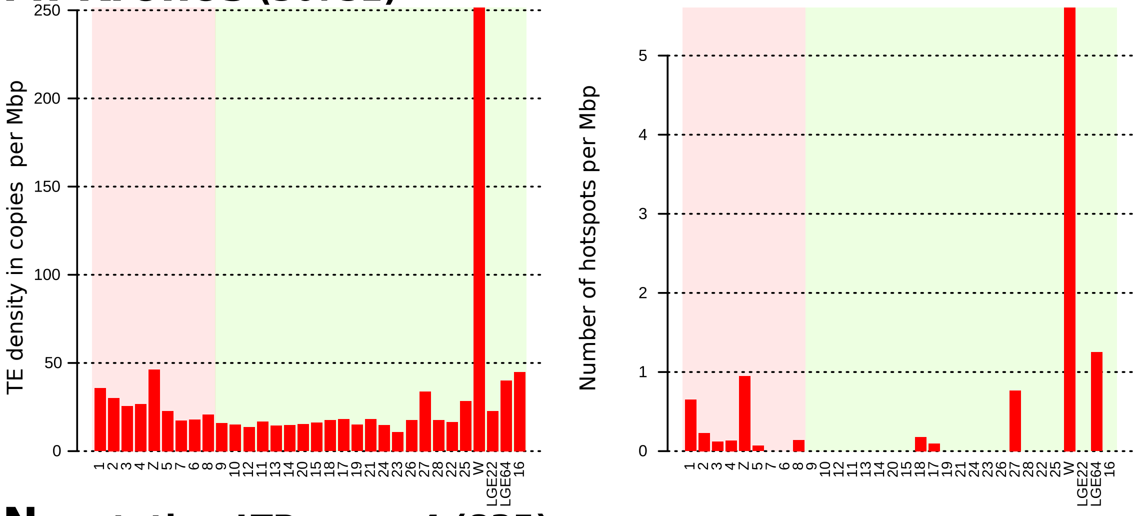


**N. putative_LTR-group4 (835)**

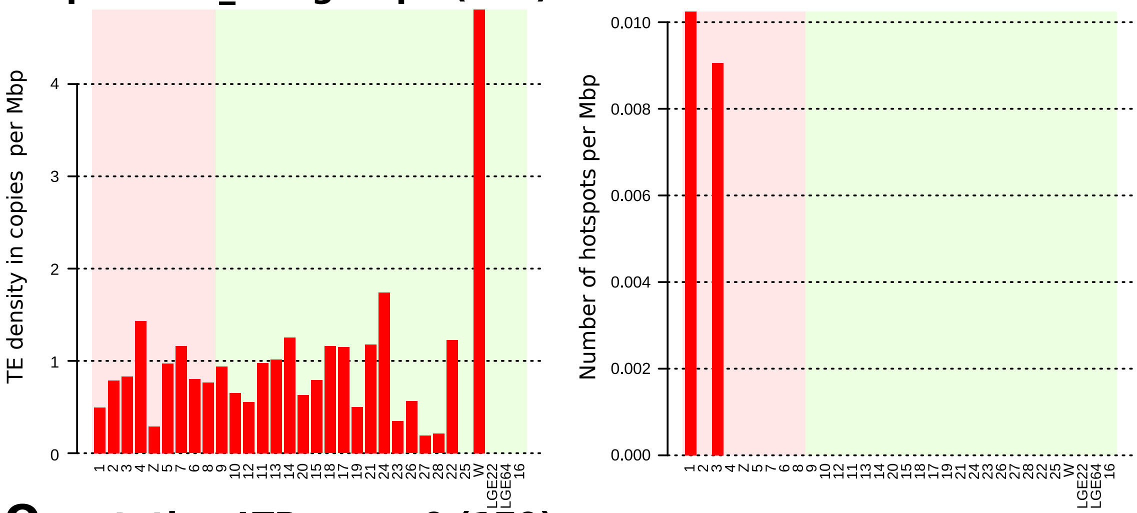


**O. putative_LTR_group9 (170)**

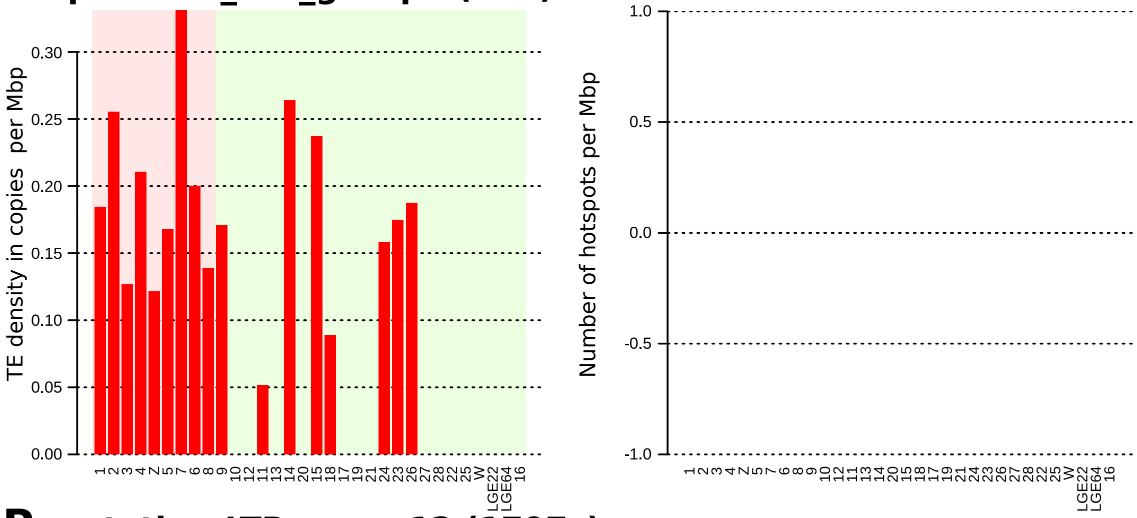


**P. putative_LTR_group12 (1797s)**

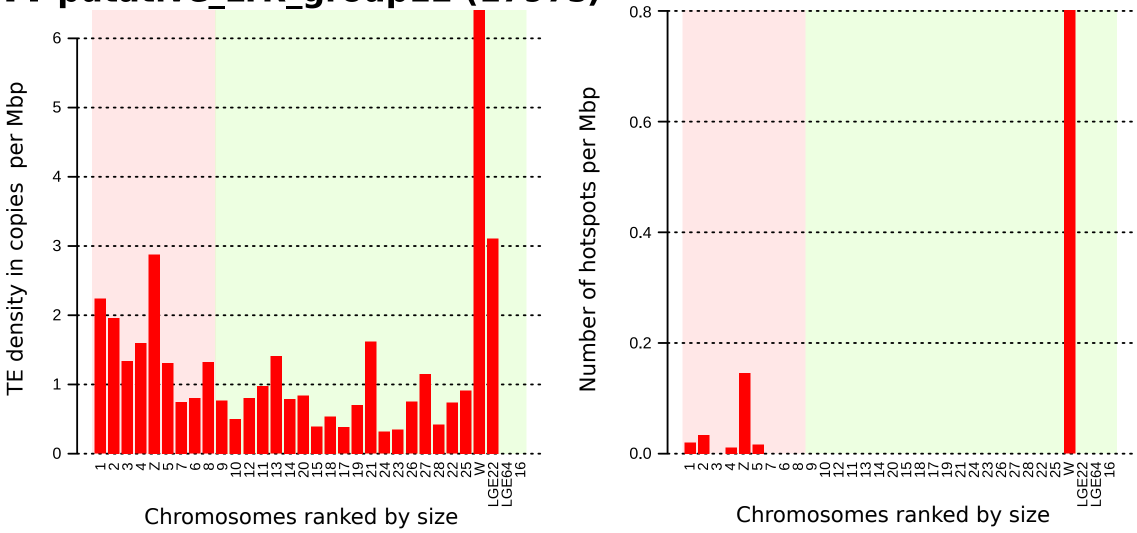

## Q. putative_LTR_group22 (1219)

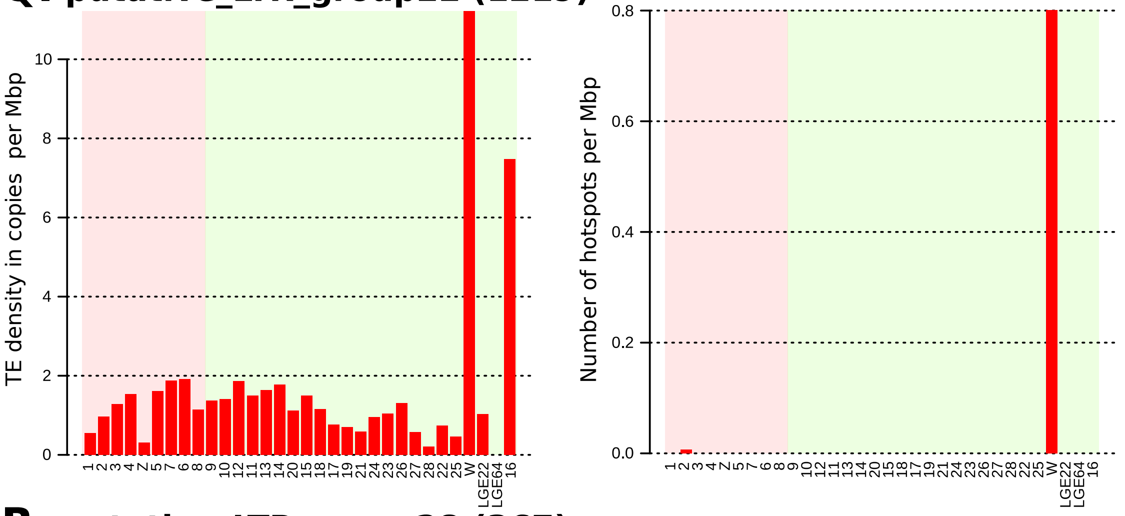


## R. putative_LTR-group28 (367)

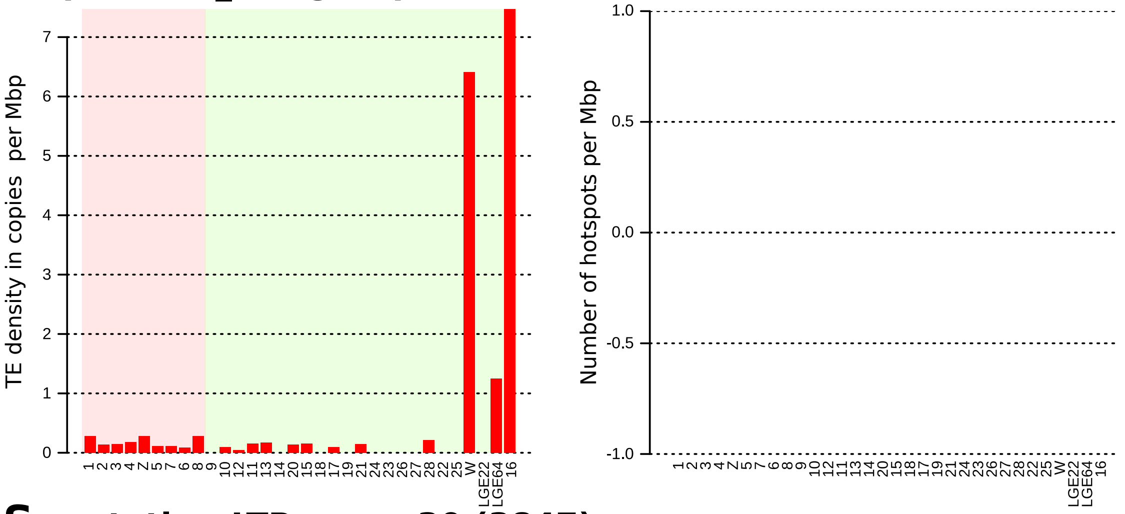


## S. putative_LTR_group30 (3847)

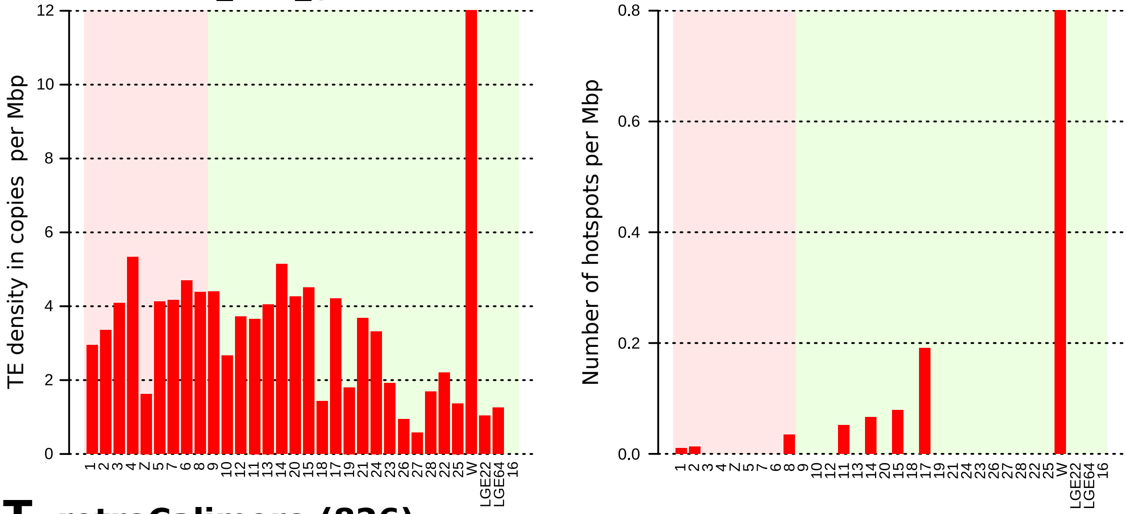


## T. retroCalimero (826)

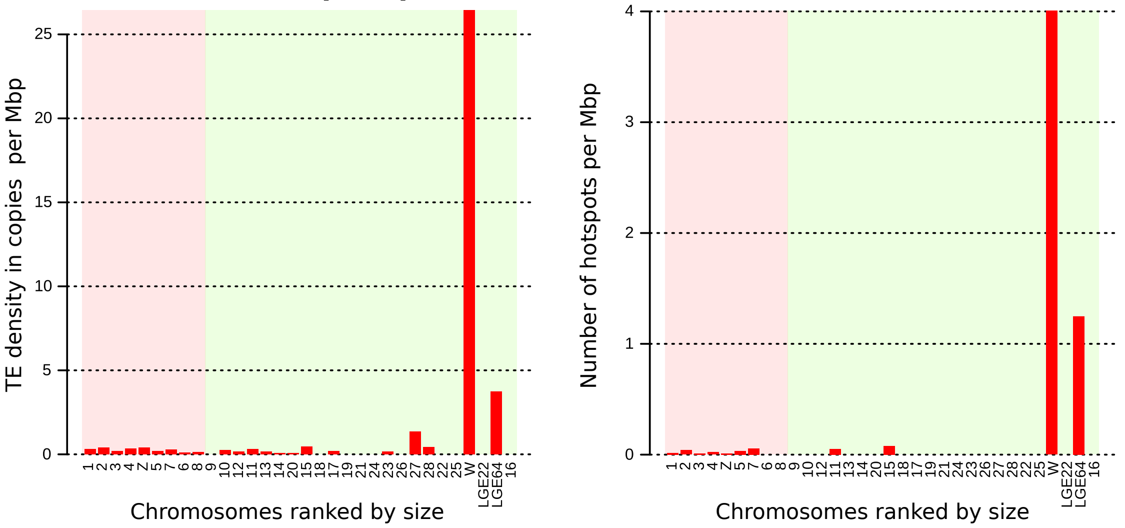

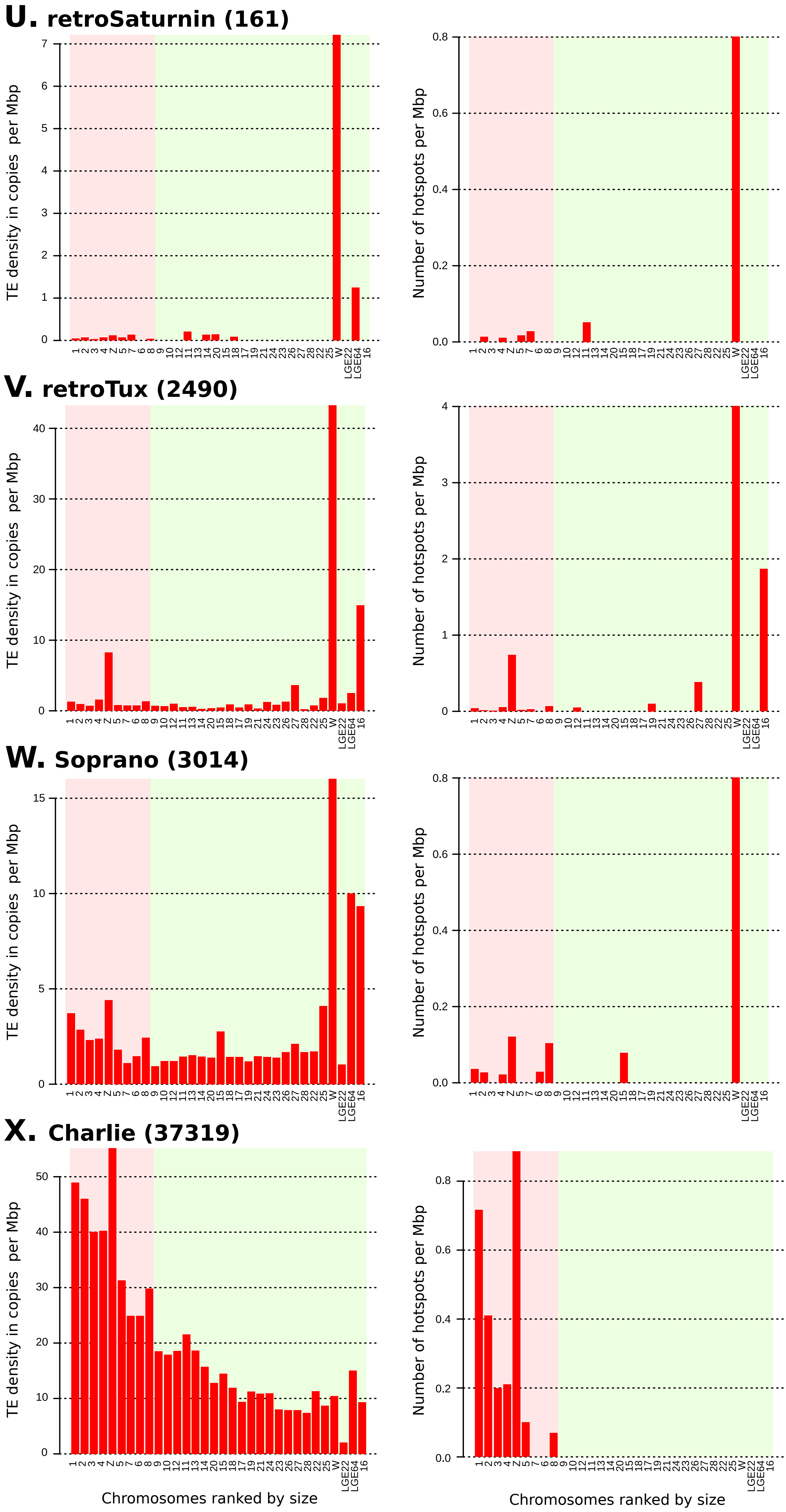

U. retroSaturnin (161)
V. retroTux (2490)
W. Soprano (3014)
X. Charlie (37319)
TE density in copies per Mbp
Number of hotspots per Mbp
Chromosomes ranked by size
Chromosomes ranked by size
1 2 3 4 Z 5 7 6 8 9 10 12 11 13 14 20 15 18 17 19 21 24 23 26 27 28 22 25 W LGE22 LGE64 16

**Y.** **Charlie-Galluhop (67691)**

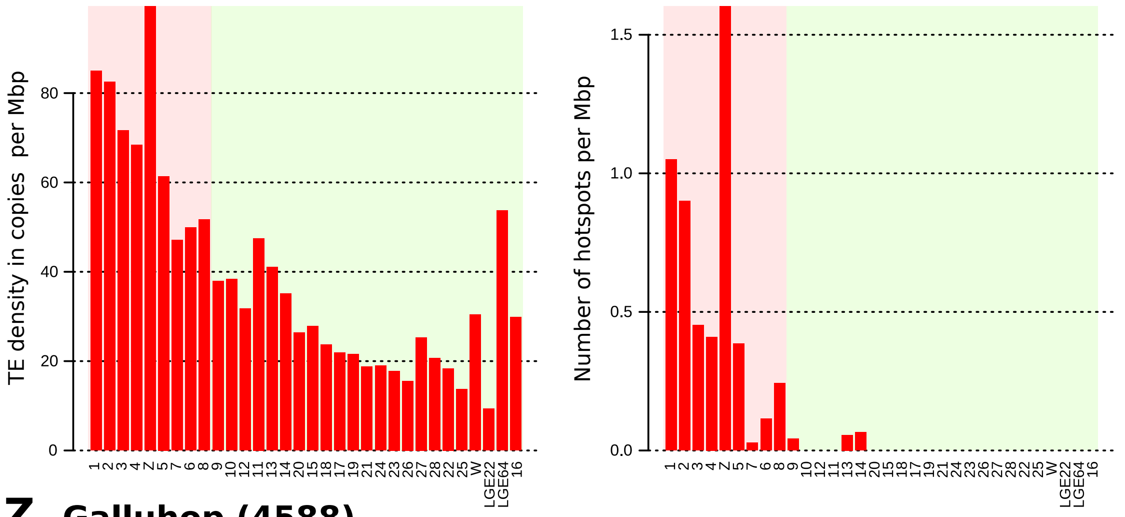


**Z.** **Galluhop (4588)**

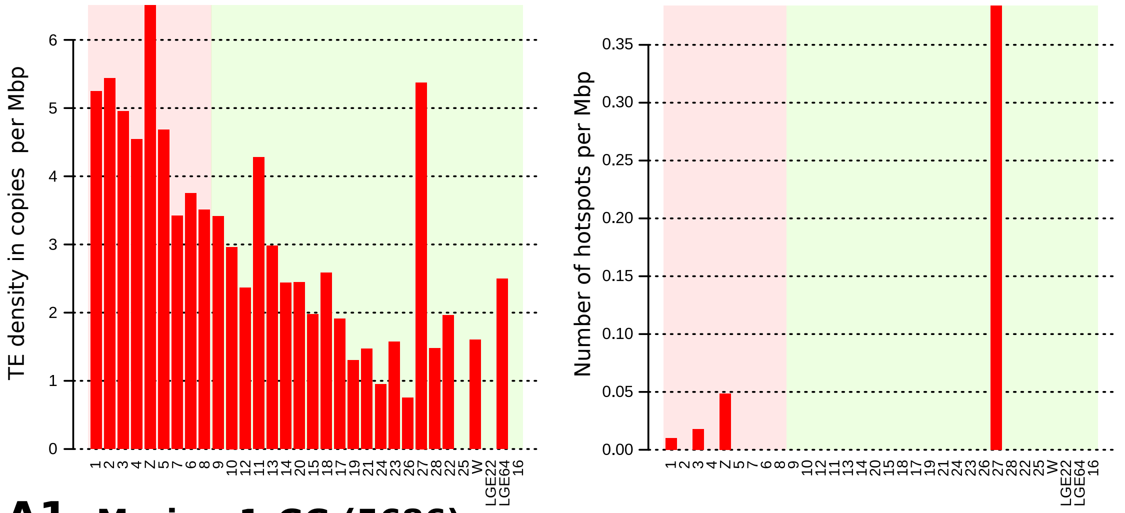


**A1.** **Mariner1_GG (5686)**

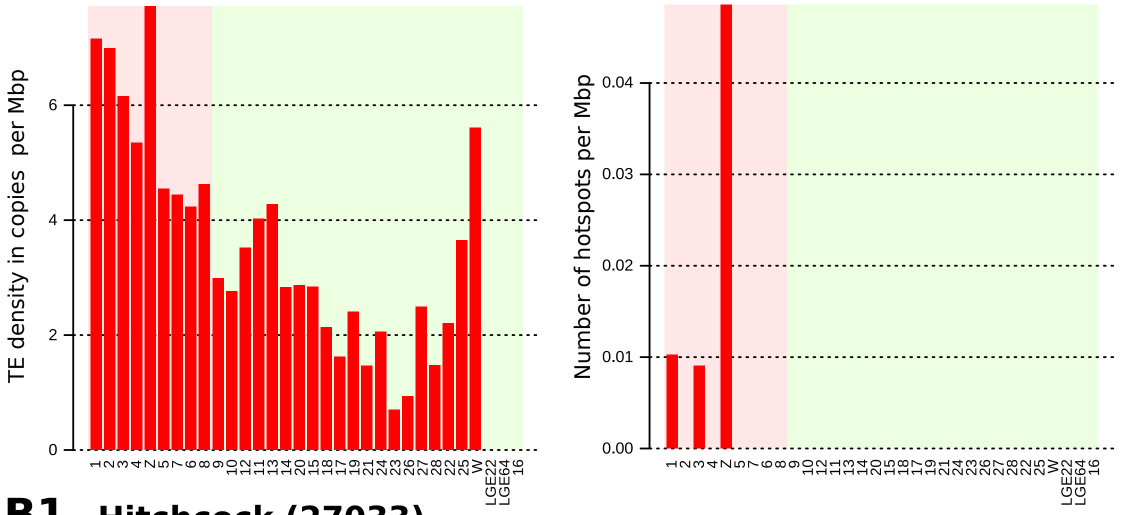


**B1.** **Hitchcock (27033)**

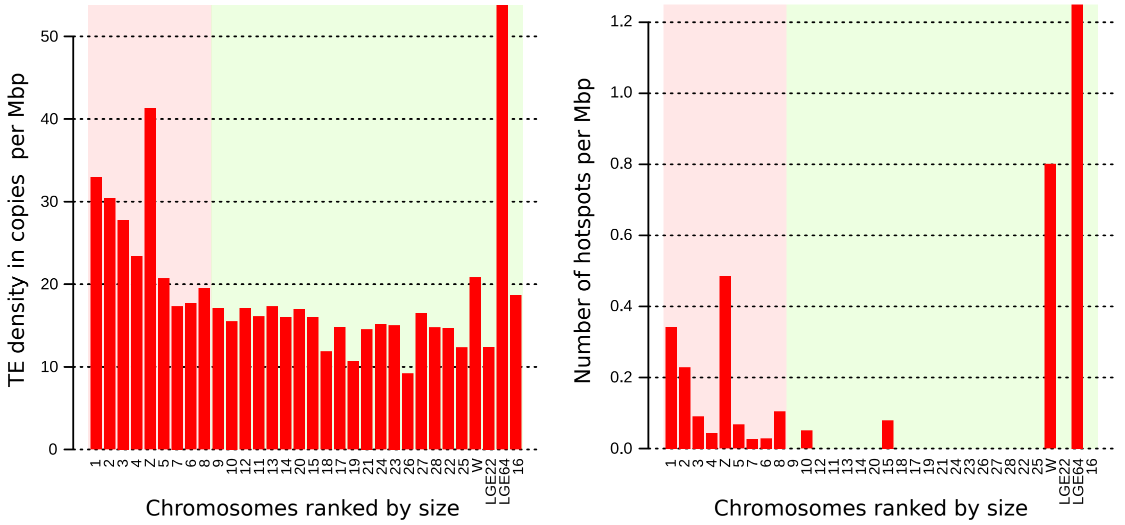

**C1. undetermined_group_1 (2219)**

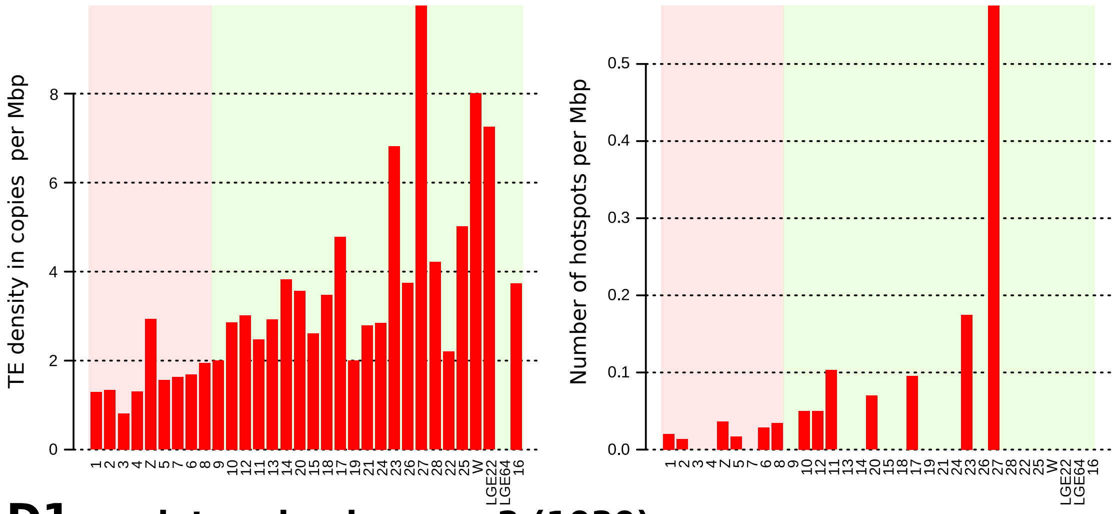


**D1. undetermined_group_2 (1030)**

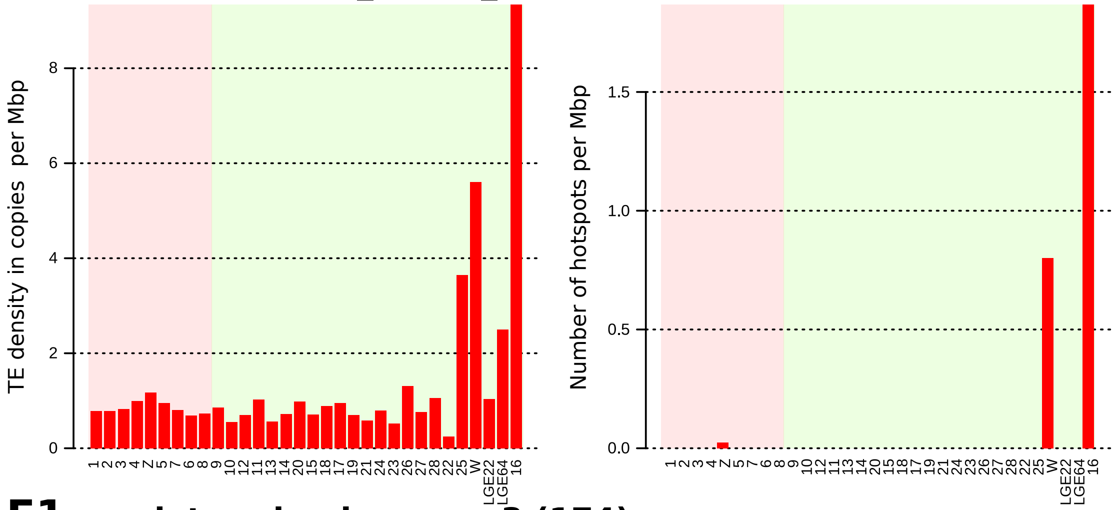


**E1. undetermined _group_3 (174)**

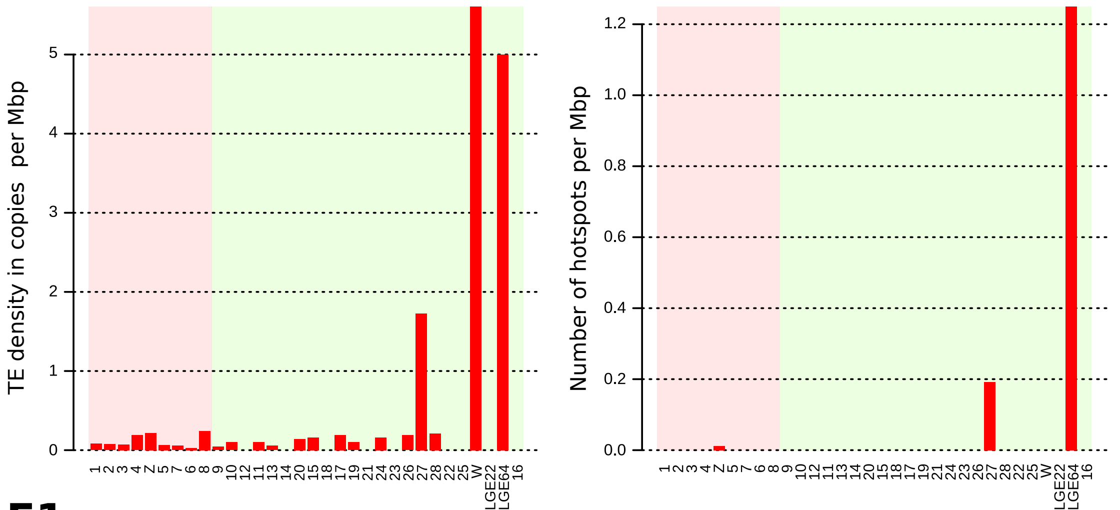


**F1. undetermined_group_4 (2550)**

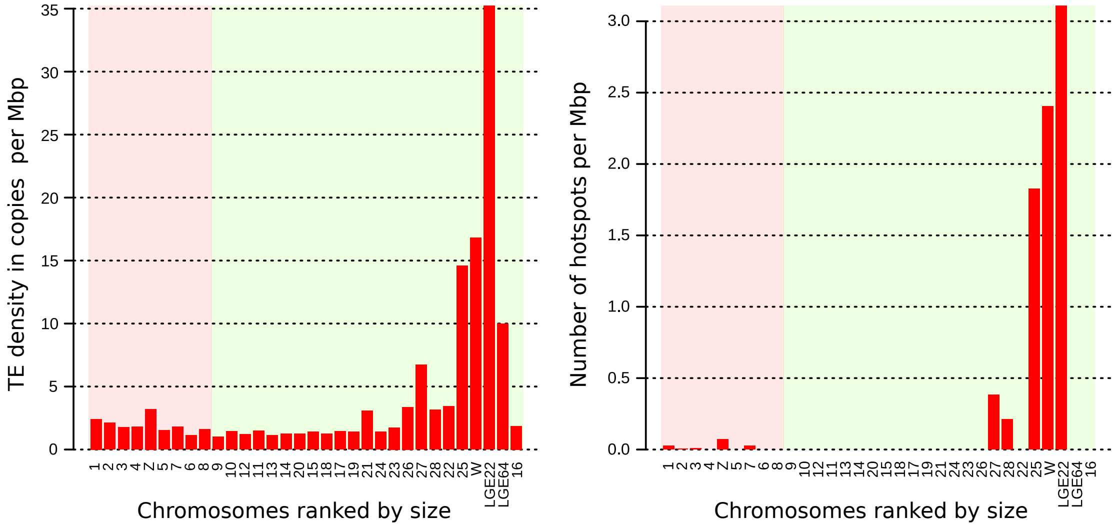

**G1. undetermined_group_5 (134)**

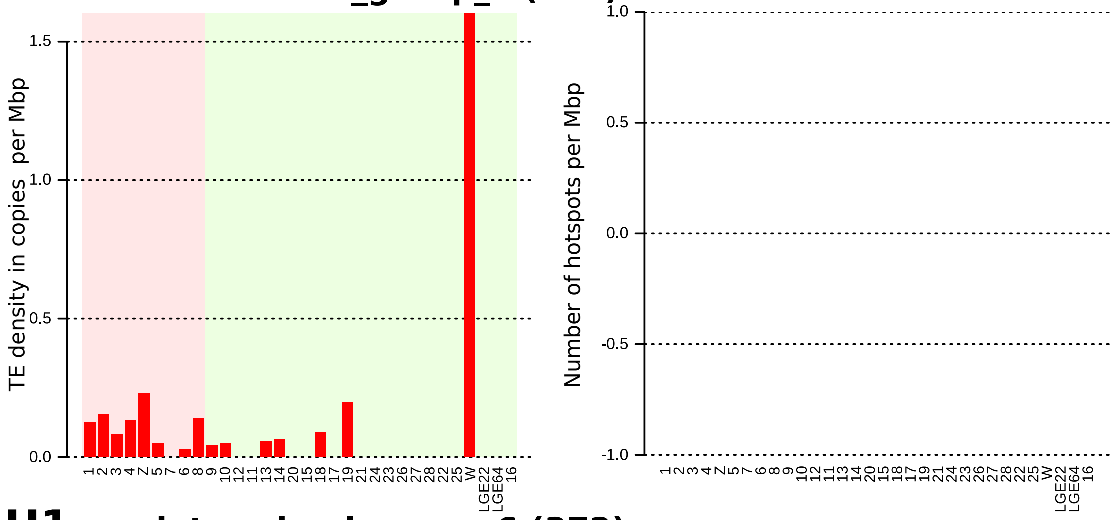


**H1. undetermined_group_6 (372)**

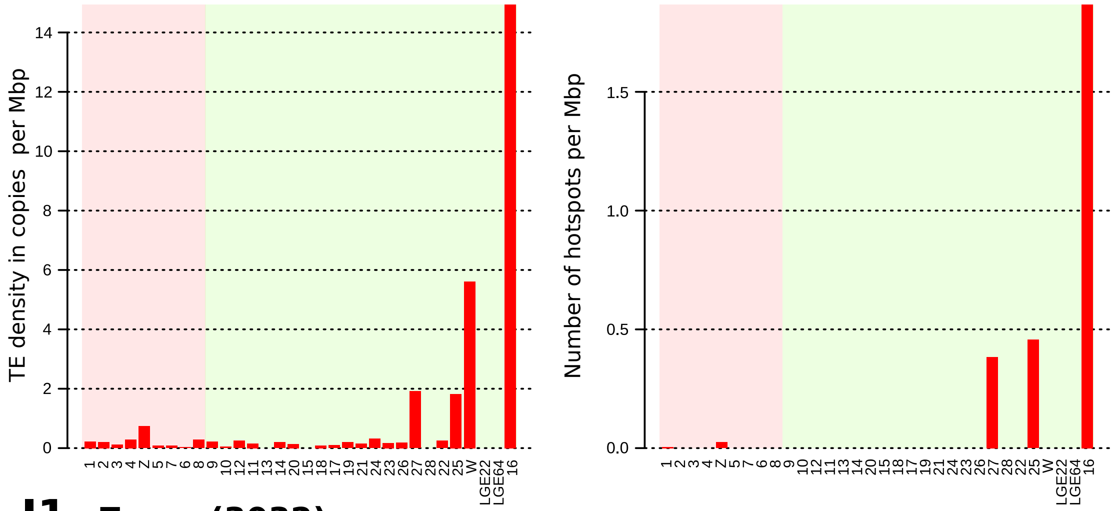


**I1. Z_rep (3032)**

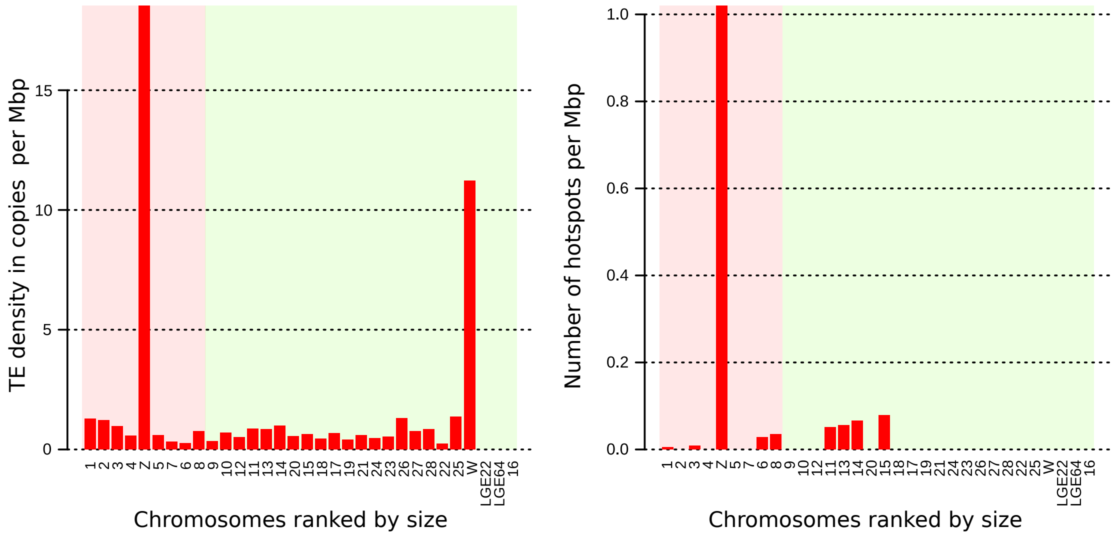

Supplement: Additional file 17: — Histograms showing the densities of TEs (left column) and TE hot spots in galGal4 chromosomes for all TEs plus each of the 34 TE models. Histograms of TE model density and TE hot spot density were calculated for all galGal4 chromosomes, except chromosome 32 (1028 bp). (PDF 2152 kb) [file 12864_2016_3015_MOESM17_ESM.pdf]
