# Supplementary material for: Deep landscape update of dispersed and tandem repeats in the genome model of the red jungle fowl, Gallus gallus, using a series of de novo investigating tools
Source: BMC Genomics. 2016 Aug 19;17:659. doi: 10.1186/s12864-016-3015-5 (PMC4992247; doi:10.1186/s12864-016-3015-5)
Supplement: Supplementary file 18 [file 12864_2016_3015_MOESM10_ESM.odt]

Additional file 10: Features of 8 repeat models that cannot be assigned to a known eukaryotic TE

Twenty seven consensuses gathered into 8 TE models (Table 5) had features that  could not be classified. Seven of them (18 consensuses), Hitchcock and undetermined_group_1 to 6 (Additional file 7), have previously been described as being solo LTRs [60]. In general, LTR elements had short inverted repeats at their ends, typically 5'-TG-3' and 5'-CA-3'[^1]. Solo LTRs were flanked by a short 3-6 bp motif at both ends that varied in sequence from one locus to another. Copies of undetermined_group_1 to 6 had no 5'-TG-3' and 5'-CA-3' at ends. However, some of them had 3-6 bp repeats or sequences at their ends. Together, this suggests that undetermined_group_1 to 6 were mobile sequences or were derived from them. However, the above evidence was not sufficient to assert that they were solo LTRs. With regard to undetermined_group_1, we observed that copies of this element displayed two hairpin loops on their ends. Such terminal structures have previously been described in some bacterial insertion sequences (IS), such as the IS with a HUH transposase[^2]. Few loci with with potential HUH motifs were found in Galliforme species genomes with e-values less than 1 e-20. We considered that these data were not reliable because we failed to extend such similarities to other conserved motifs presents in HUH transposases.

The last nine consensuses were gathers into one model named Z-rep that describes repeats contained in a macro-satellite DNA mainly present at one end of the Z chromosomes and dispersed in the W chromosome. These nine consensuses described the ~22 kbps. units found in tandem in this satellite DNA. These were composed of a mix of old TE fragments originating from CR1 and LTR retroviruses. Interestingly, Z-rep sequences were only located in a single terminal block of 9 Mbps. juxtaposed near a telomere involved in the lampbrush structure during the prophase I stage of meiosis[^3].  They were also found sporadically over the entire length of chromosome Z (Figure S3).

Fig. S3 Dot plot representation of a pair sequence comparison calculated with the complete sequence of chromosome Z using Mummer[^4]. All repeated segments along the chromosome Z were visualized by blue spots, except for those corresponding to the Z_rep sequences that were highlighted by yellow spots. Horizontal and vertical axes were scaled in Mbp.

[^1]: Yin H1, Liu J, Xu Y, Liu X, Zhang S, et al. TARE1, a mutated Copia-like LTR retrotransposon followed by recent massive amplification in tomato. PLoS One. 2013;8:e68587.
[^2]: Chandler M, de la Cruz F, Dyda F, Hickman AB, Moncalian G, et al. 2013. Breaking and joining single-stranded DNA: the HUH endonuclease superfamily. Nat Rev Microbiol. 11:525-538.
[^3]: Andraszek K, Smalec E: Structure and functions of lampbrush chromosomes. BioTechnologia 2011, 4:337–344.
[^4]: Kurtz S, Phillippy A, Delcher AL, Smoot M, Shumway M, et al. Versatile and open software for comparing large genomes. Genome Biol. 2004;5:R12.
