# Supplementary material for: Deep landscape update of dispersed and tandem repeats in the genome model of the red jungle fowl, Gallus gallus, using a series of de novo investigating tools
Source: BMC Genomics. 2016 Aug 19;17:659. doi: 10.1186/s12864-016-3015-5 (PMC4992247; doi:10.1186/s12864-016-3015-5)
Supplement: Supplementary file 19 [file 12864_2016_3015_MOESM11_ESM.odt]

# Additional File 11: Proteins encoded by the remnant polinton located in the RFJ and Turkey genomes.

>GG-Polinton-Integrase [chr2:79632317-79633476]

MYCNVRYLVEKDYKGRIGEISLVLESEKPSHQQLLFSPGQYNFSVHHLVDVSQLLLEDFHHSPGRMESYHLFEVATKQIPSLNRSQVAIWLANQDAXSLGKATRKQCKRKELIVAEVKGRRQPQLVDVQQFSKQNGGGKXSFVVVGVLQKHAWAAGLKQKMGSEIVGGLXVIFIRGTXPQEMTAQGKDFLKQPLKKLLQLYNNATTNKEHFSRTLKTKMXRYFIAHNAFCYSDTFSPFIKSYNYSFHRITTATEPGKVKPLKSMGEXIKIYSXSFKFNLNXRICTFPQKERPQSARKISEKLXAFIDXIFIVAKTEKGDTCLLNRPQSQKNQRYLKKXIXIRTDYKIERIITTKGTGRKKELLVLLCGSLQIFYSCTETSLYQDIQ

>GG-Polinton-MCP [chr2:79634258-79635332]

MNYAVQVSNNLLGFQAIPLTYRTAHTFCARKRNITYTLTAYRSYHCQNRSWKLLSQVLKTSLKTLPKKKKKVFGYVSISVLVTPENDGEIKKRVRLTCTQKWPVPVFQKQKRKNQTAIWLRKKEGKQCRQCSGLTARSGRTECLGPLHCLLFQDKLFLNSTDVTIKLMHSKDSICLMGSLATVACKPVTACILLHVKKLWVALWLHLGQNEPLHVTVLNTWLTRRSWKHSTXLASSHVSNQENLFLGQLPKLHVIVFVDNDAFRGNYAXKPFNFKHKINILRIYTWLMMEPXQNHCSLIFKIGCCVKGYLQLVQRISSRAKDHICXQPKGRIHPWLYPLCLWPISKSAMHQSLLIQLK

>Turkey-Polinton-DNApolymerase [chrZ30210529-30207921]|[galGal4-chr2:79638800-79637500]

MDFIKLFMNMWMMAFQFLVHSAEGNDGYFVDSSKXGDNVQITQGGKLNCIEVLSMSTYFTDCLFVSHDIKQLFQNLNIXLGRLFPLFFQHCXKIKVYVTTLSPLEYYEVDNVMLKEKTPLYDRKTNQEKIFDLPKERCYYCQQDVQLLRGSCXXFMKXLMSIACYTFYEKKENENMTIYYCRDPFQHTTLFYTCLSRYRFMLLXNILSWKELTRIMKSNTWSIQNLNYFYELSPTASXTTAALGHDHFPGSLFTLAEPFPNCHLTLLXDNSMPFPXVLSPSPESRVQHCPSTPLVRSFRPPXGFFSVSSALTXTQQGSSATPHILPSRPLPSTVLSNAFVFFILWHPKIPSAXSEATQLRAERDNHTPHPVAVLGLAYPRGXLTLLAARTHSGLMFILSSTKTPRFLSAGLLSSILWLMYVAKKGKSXICLVLXGTEHQIESXFLDSYMVIDMVLTAFKLNGCFFHGCIKCYDKNTWQGPHLIGAPKVLAVKTMREHVWTTFXWGDXRVFSXSLKRCLSAAGQVLCLYYKADIEEKYSYXKQPGLWHXQQRRSACVYLVFLRTEQGSREELQTLXDLXESCFEXRVRNLFSDHIKLYLYQKHEASGXPASHRDDEENKXINKXMYXRLXAAGRCHFAAWARRKNSAIYKIAKLLLSCLWEKIQTEIQLSNTIVREQDELYCYFFSPAFKVSSRDFTDKNIAILSXTLKEMXTRDFYDVLHLGIFVPXALCMPCCVYHISVIIMSHLGARKDPSGNDSGLLMGELLXNEHITELASSSPKLYGYVRSGGRCCLKVKGISLTXXKARVVTKIFKSLPYIIILGMHWKHISFVALEGSSNGR
