# Supplementary material for: Deep landscape update of dispersed and tandem repeats in the genome model of the red jungle fowl, Gallus gallus, using a series of de novo investigating tools
Source: BMC Genomics. 2016 Aug 19;17:659. doi: 10.1186/s12864-016-3015-5 (PMC4992247; doi:10.1186/s12864-016-3015-5)
Supplement: Supplementary file 20 [file 12864_2016_3015_MOESM12_ESM.odt]

Additional File 12. Characteristics of 54 neogenes derived from DNA transposons in the human genome and RJF (typed in blue) genomes.

| Transposon family on origin | Related DNA transposon | Neogene names & synonyms in human | Protein size (aa)* | Acc. Number in the NBRF database | Chromosomal location in human | Chicken orthologs (NCBI or ENSEMBL) | Protein size (aa)* | Chromosomal location in chicken or taxonomic presence |
| --- | --- | --- | --- | --- | --- | --- | --- | --- |
| Ginger | Ginger1 | GIN1 (C5orf30) | 522 | NP_060146.2 | 5q21.1 | XP_004949361 | 526 | Z |
|  | Ginger2 | KRBA2 | 492 | NP_998762.1 | 17p13.1 |  |  | P only in Eutheria |
|  | Ginger2-hobo | SCAND3 (ZBED9; Buster4 ; Charlie 10; ZNF452) | 1325 | NP_443155 | 6p21.33 |  |  | P only in Theria |
|  | hobo | ZBED1 (hDREF) | 694 | NP_004720 | Xp22.33 & Yp11 | XP_004938502 | 695 | 1 |
|  | hobo | ZBED2 | 218 | NP_078784 | 3q13.2 |  |  | P only in Eutheria |
|  | hobo | ZBED3 | 234 | NP_115743 | 5q13.3 |  |  | P only in Eutheria |
|  | hobo | ZBED4 | 1171 | NP_055653 | 22q13.33 | NM_001199541.1 | 1172 | 1 |
|  | hobo | ZBED5 (Charlie 1 ; Buster 1) | 693 | NP_001137139.1 | 11p15.3 |  |  | P in Amniota, L in Sauropsides |
|  | hobo | ZBED6 | 979 | NP_001167579.1 | 1q32 |  |  | P in Amniota, L in Birds |
|  | hobo | GTF2IRD2 (Charlie 8) | 949 | NP_001003795.1 | 7q11.23 | XP_004946491 | 1062 | 19 |
|  | hobo | ZBED8 (Buster3 ) | 594 | NP_071373.2 | 5q33.3 |  |  | P in Chordata; L in Birds |
| IS630-Tc1-mariner | Pogo | Centromer protein B (CENP-B) | 599 | [NP_001801.1](http://www.ncbi.nlm.nih.gov.gate1.inist.fr/protein/21735415) | 20p13 |  |  | P only in Theria |
|  | Pogo | Jerky (JRK) | 568 | NP_003715.2 | 8q24.3 |  |  | P in Sauropsides, L in Birds |
|  | Pogo | Jerky-like (JRKL | 524 | NP_001248762.1 | 11q21 |  |  | P in Tetraopoda, L in Sauropsides |
|  | Pogo | Tigger TE-derived 1 (TIGD1) | 591 | NP_663748 | 2q37.1 |  |  | P in Boreoeutheria, L in Birds |
|  | Pogo | Tigger TE-derived 2 (TIGD2) | 525 | NP_663761 | 4q22.1 |  |  | P in Tetrapoda, L in Birds |
|  | Pogo | Tigger TE-derived 3 (TIGD3) | 471 | [NP_663771.1](http://www.ncbi.nlm.nih.gov.gate1.inist.fr/protein/23312394) | 11q13.1 | ENSGALT00000043192 | 431 | 2 |
|  | Pogo | Tigger TE-derived 4 (TIGD4) | 512 | NP_663772 | 4q31.3 | ENSTGUG00000005046 |  | 4 |
|  | Pogo | Tigger TE-derived 5 (TIGD5) | 593 | [NP_116251.3](http://www.ncbi.nlm.nih.gov.gate1.inist.fr/protein/144922631) | 8q24.3 | XP_004940149 | 544 | 2 |
|  | Pogo | Tigger TE-derived 6 (TIGD6) | 521 | [NP_112215.1](http://www.ncbi.nlm.nih.gov.gate1.inist.fr/protein/13569924) | 5q32 |  |  | P in Tetrapoda, L in Sauropsides |
|  | Pogo | Tigger TE-derived 7 (TIGD7) | 549 | NP_149985 | 16p13.3 |  |  | P only in Theria |
|  | Tc2 | POGK | 609 | [NP_060012.3](http://www.ncbi.nlm.nih.gov.gate1.inist.fr/protein/22027480) | 1q24.1 |  |  | Gene fossil in Chromosome 3 [GG4: 125200322 - 125201393] |
|  | Tc2 | POGZ | 1410 | NP_055915.2 | 1q21.3 | XP_004948432.1 | 1386 | 25 |
|  | Hsmar1 | SETMAR (Metnase) | 684 | NP_006506.3 | 3p26.1 |  |  | Own to Primate |
| P | P | THAP0 ([PRKRIR](http://www.ncbi.nlm.nih.gov.gate1.inist.fr/sites/entrez?Db=gene&Cmd=retrieve&dopt=full_report&list_uids=5612&log$=databasead&logdbfrom=protein); DAP4) | 761 | NP_004696.2 | 11q13.5 | XP_425679 | 891 | 1 |
|  | P | THAP1 | 213 | NP_060575.1 | 8p11.21 | XP_004949398 | 244 | Z |
|  | P | THAP2 | 228 | NP_113623.1 | 12q21.1 |  |  | P in  Euteleostomi , L in Birds |
|  | P | THAP3 | 175 | NP_612359 | 1p36.31 |  |  | P in  Euteleostomi , L in Birds |
|  | P | THAP4 | 577 | NP_057047.3 | 2q37.3 | XP_004937062 | 202 | 9 |
|  | P | THAP5 | 395 | NP_001123947 | 7q31.1 | XP_416027 | 413 | 1 |
|  | P | THAP6 | 222 | NP_653322 | 4q21.1 |  |  | P in  Euteleostomi , L in Birds |
|  | P | THAP7 | 309 | NP_001008695.1 | 22q11.2 | XP_001235650 | 267 | Scaffold AADN03026676.1 [GG4: 79-2997] |
|  | P | THAP8 | 274 | NP_689871 | 19q13.12 |  |  | P in Amniota, L in Birds |
|  | P | THAP9 (Phsa) | 903 | NP_078948.3 | 4q21.22 | XP_420555.4 | 857 | 4 |
|  | P | THAP10 | 257 | NP_064532 | 15q23 |  |  | P in Eutheria |
|  | P | THAP11 | 314 | NP_065190 | 16q22.1 | ENSACAT00000001443 | 277 | 11 |
| PIF-Harbinger | Harbinger | HARBI1 | 349 | NP_776172 | 11p11.2 | XP_421117 | 348 | 5 |
|  | Harbinger | nuclear apoptosis-inducing factor 1 (NAIF1) | 327 | NP_931045 | 9q34.11 | XP_415512 | 330 | 17 |
| piggyBac | piggyBac | PiggyBac-derived 1 (PGBD1) | 809 | NP_115896.1 | 6p22.1 |  |  | P only in Theria |
|  | piggyBac | PiggyBac-derived 2 (PGBD2) | 341 | NP_001017434.1 | 1q44 |  |  | P only in Theria |
|  | piggyBac | PiggyBac-derived 3 (PGBD3) | 593 | NP_736609 | 10q11 |  |  | P only in Strepsirrhini |
|  | piggyBac | PiggyBac-derived 4 (PGBD4) | 585 | NP_689808.2 | 15q14 |  |  | P in Chordata; L in Sauria |
|  | piggyBac | PiggyBac-derived 5 (PGBD5) | 554 | NP_078830.2 | 1q42.13 | XP_004935545 | 409 | 3 |
| Polinton-Marverik | Polinton | KRBA (KRAB-A1) | 1030 | NP_115923.2 | 7q36 |  |  | P only in Theria |
| Transib | Transib | RAG1 | 1043 | NP_000439.1 | 11p13 | XP_421090 | 1041 | 5 |
| Transib | Transib | RAG2 | 527 | NP_001230715.1 | 11p13 | AAA49052.1 | 528 | 5 |
| Transposon family on origin | Related DNA transposon | Neogene names & synonyms in human | Protein size (aa)* | Acc. Number in the NBRF database | Chromosomal location in human | Chicken orthologs (NCBI or ENSEMBL) | Protein size (aa)* | Chromosomal location in chicken or taxonomic presence |
| Crypton | Crypton | KCTD1 | 865 | NP_001136202 | 18q11.2 | XP_004939868 | 267 | 2 |
|  | Crypton | KIAA1958a | 744 | NP_597722 | 9q32 | XP_004949571 | 733 | Z |
|  | Crypton | QRICH1 | 776 | NP_060200 | 3p21.31 | XP_003642053 | 773 | 12 |
|  | Crypton | ZMYM2 | 1377 | NP_003444 | 13q11-q12 | XP_004938872 | 732 | 1 |
|  | Crypton | ZMYM3 | 495 | NP_001164634 | Xq13.1 | XP_420201 | 1433 | 4 |
|  | Crypton | ZMYM4 | 1548 | NP_005086 | 1p32-p34 | XP_004947829 | 1514 | 23 |
|  | Crypton | ATF7IP | 1270 | NP_060649 | 12p13.1 | XP_416205 | 1085 | 1 |
| Zirupton | Zisupton | HMGXB3 | 1106 | NP_002600 | 5q33.1 | XP_414598 | 1453 | 13 |

*, sizes in amino acids (a.a.) were calculated from the largest ORF found in mRNA; **, DBD = DNA binding domain and CatD = catalytic domain. ?, located domains with no definitely characterized function; ***, the presence of neogenic orthologues in databases was checked using BLASP and genome browsers at http://blast.ncbi.nlm.nih.gov.gate1.inist.fr/Blast.cgi?PROGRAM=blastp&BLAST_PROGRAMS=blastp&PAGE_TYPE=BlastSearch&SHOW_DEFAULTS=on&LINK_LOC=blasthome, [http://genome.ucsc.edu/](http://genome.ucsc.edu/), http://www.dyogen.ens.fr/genomicus-67.01/cgi-bin/search.pl.  Related neogenes are listed in families and alternately shaded in grey or unshaded. In the last Column, P =Presence and L : Lost.
