# Supplementary material for: Deep landscape update of dispersed and tandem repeats in the genome model of the red jungle fowl, Gallus gallus, using a series of de novo investigating tools
Source: BMC Genomics. 2016 Aug 19;17:659. doi: 10.1186/s12864-016-3015-5 (PMC4992247; doi:10.1186/s12864-016-3015-5)
Supplement: Supplementary file 21 [file 12864_2016_3015_MOESM14_ESM.odt]

Additional file 14: Origins of differences between ISB and [TE+DM] annotations.

171 Repbase and ISB TE consensuses had no equivalent in our TE models. The origins of these differences were investigated in order to: i) verify how loci were annotated in both annotation methodologies and ii) analyze the quality of the ISB annotations that were devoid of an annotation by our procedure.

The identities and differences between the [TE+DM] and the ISB annotations were first investigated locus by locus using bedtools[^1]. A large majority (85.4%, Figure 7a) of the most abundant TEs (including CR1, Galluhop, Kronos, Charlie, and BIRDDAWG [60]) had identical [TE+DM]  and ISB annotations. However, 7.4% of ISB annotations did not match with those of the corresponding model in the [TE+DM] annotations (Figure S4a). 7.3% of the ISB annotations had no corresponding matches in the de novo [TE+DM] annotations and were annotated with one of the 171 Repbase and ISB TE consensuses (Figure S4a).

We identified 72,090 loci with annotations that differed between [TE+DM] and ISB and selected 100 of these for manual annotation. 

Figures S4b1, b2 and b3 illustrate 3 cases where the RM annotations did not match those of the corresponding model in the de novo [TE+DM] annotation. These cases are representatives of the majority (75 %) of those that were sampled. ISB annotations appeared less precise for describing TE copies than those calculated in the de novo [TE+DM] annotations. Indeed, they did not take into account small TEs or pieces of TE inserted into larger TEs. Although rarer, annotations that were erroneous and/or ambiguous were also found in the [TE+DM] annotations. Figure 8C illustrates a case of erroneously annotated loci in the de novo [TE+DM] annotation. Because the ENS3-Int consensus described a non repeated sequence it was  not identified by REPET. Figure 8D describes a locus that appeared similar to the case described in Figure S4c but for which a detailed analysis of sequence similarities with the involved consensuses did not allow us to make a decision. Therefore, our investigations indicated that the library dependent steps in both approaches (RM versus TEannot) remained a delicate step for which the quality of the annotation fully depended on the supplied sequence library.

Fig. S4 Quality of [TE+DM] and ISB annotations. A, pie chart representing the correspondences of the ISB annotations within the [TE+DM] annotations. Slices corresponded to coverage percentages of ISB annotations that were identical to those of the [TE+DM] annotation (blue), did not matched with those of the corresponding model in the [TE+DM]  annotations (orange), or had no corresponding annotation in the the [TE+DM]  annotations (yellow) and were annotated with one of the 171 Repbase and ISB TE consensuses own to the ISB annotation (yellow). B to D, Examples of five loci differentially annotated in the de novo TE+DM and ISB annotations. For each locus, the positions were supplied for the REPET and RM annotations: B1, chr2:54915707..54918245 / chr2:54915760..54918231, B2, chr2:87534124..87535508 / chr2:87534124..87535507, B3, chr1:157232263..157234613 / chr1:157232241..157235461, C, chr8:8963651..8968051 / chr8:8963650..8966367, D, chrZ: chrZ:29482798..29486975 / 29483193..29486961. Annotations resulting  from REPET annotations and the DM annotations were respectively indicated by (TE) and (DM). This allowed visualizing to which extend the DM annotations enlarged those made by REPET in these five examples. Back vertical lines in CR1 REPET annotation in B1, B2 and B3 indicated that the different regions were annotated by different consensuses belonging to the CR1 model.

The [TE+DM] and ISB annotations shared 8.9% coverage and 0.8% were specific to the ISB and resulted from annotations performed with one of the 171 Repbase and ISB TE consensuses. This 0.8% was divided into 0.04% for ISB annotations for genes coding structural RNA and 0.76% corresponded to annotations of TEs such as LINEs, LTR, Eulor, UCON, MER, SINEs, IS and DNA transposon that were not detected by REPET as being repeats (because of their sequence divergence (sequence divergence with their Repbase and ISB consensuses ranged from 20 to 40%; Additional file 13) or their size (311 RM annotation are smaller than 20bp)). Forty percent of TE RM annotations had sequence divergence from their consensus of 20% or more (124,555 loci ranging from 10 to 5625bp). To create a consensus REPET needs at least 3 similar copies (>95 %) and it tends to be less efficient with repeats  inactivated for mobility for a long time.

The consistency of the RM annotations made from 171 Repbase and ISB TE consensuses was investigated and we focussed on a sub-sample that gathered consensuses annotating SINE element (AmnSINE1 (574 bp), AMnSINE2 (358 bp), LMESINE1c (404 bp), MER129 (469 bp), MER130 (475 bp) and MER131 (173 bp)), Harbinger transposons (Harbinger-N1_Croc (88 pb), Harbinger-N276_Ami (110 bp), Harbinger-N96_Ami (79 bp)), and Kolobok transposons (UCON29 (437 bp) and UCON2 (280 bp)). Annotated copies with each of these consensuses were  extracted into a single file into which their Repbase consensus were added to help drive their alignment using Muscle. We found that it was not possible to consistently align the extracted copies because numerous copies were too small(Additional file 13) and were at least 30 to 50% divergent from each other (although they were all 20 to 30 % similar to their consensus). Together, the analysis of these cases (4,298 RM annotations) led us conclude that a significant part of these annotations are likely artefacts inherent to library-based annotation methods. Regarding SINEs, the conclusion of our investigations agreed with recent results that support avian CR1 mediated transposition involves specific nucleic structure in CR1s that hamper the retrotransposition of pseudogenes[^2]. Another issue was that part of the consensuses (in our sampling LFSINE_vert (SINE) and Penelope1_Vert (non-LTR retrotransposons) corresponding to those defined by the ISB were not publicly accessible. Therefore, the quality of their annotations could not be verified.

[^1]: Quinlan AR1, Hall IM. BEDTools: a flexible suite of utilities for comparing genomic features. Bioinformatics. 2010 26:841-8422.
[^2]: Suh A. The Specific Requirements for CR1 Retrotransposition Explain the Scarcity of Retrogenes in Birds. J Mol Evol. 2015;81:18-20.
