# Supplementary material for: Deep landscape update of dispersed and tandem repeats in the genome model of the red jungle fowl, Gallus gallus, using a series of de novo investigating tools
Source: BMC Genomics. 2016 Aug 19;17:659. doi: 10.1186/s12864-016-3015-5 (PMC4992247; doi:10.1186/s12864-016-3015-5)
Supplement: Supplementary file 22 [file 12864_2016_3015_MOESM15_ESM.odt]

# Additional File 15: Graph showing the expected and observed coverages of TEs (A), genes (B), S/MAR (C) and CpG islands (D) in galGal4 chromosomes. Each box was calculated from 1000 permutations and represents 98% of randomized TE distributions. Red crosses above the boxes indicate over-representation of the element in the chromosome and blue crosses an under-representation (p>99% in both cases). Pink backgrounds indicate macrochromosomes and green backgrounds indicate microchromosomes. All galGal4 chromosomes, except chromosome 32 which is only 1,028 bases long, were included in the analysis.

#
