# Supplementary material for: Deep landscape update of dispersed and tandem repeats in the genome model of the red jungle fowl, Gallus gallus, using a series of de novo investigating tools
Source: BMC Genomics. 2016 Aug 19;17:659. doi: 10.1186/s12864-016-3015-5 (PMC4992247; doi:10.1186/s12864-016-3015-5)
Supplement: Supplementary file 23 [file 12864_2016_3015_MOESM18_ESM.odt]

# Additional File 18: Graph showing thresholds calculated in permutation assays and windows calculated along chromosomes for permutation tests designed to inventory hot spots.

# A. Thresholds

# 

# B. Features of windows calculated for permutation assays designed to inventory TE hotspots

#
