# Supplementary material for: Deep landscape update of dispersed and tandem repeats in the genome model of the red jungle fowl, Gallus gallus, using a series of de novo investigating tools
Source: BMC Genomics. 2016 Aug 19;17:659. doi: 10.1186/s12864-016-3015-5 (PMC4992247; doi:10.1186/s12864-016-3015-5)
Supplement: Supplementary file 24 [file 12864_2016_3015_MOESM1_ESM.odt]

# Additional file 1: Conditions of use for programs P-clouds and Red.

P-clouds has previously been used to calculate the rate of repeats in the human genome (63-69% of reported repeats; [64]) and in the Arabidopsis thaliana genome (11%; [25]). This tool uses a k-mer method to evaluate the amount of repeats.  A number of parameters must be optimized for each genome analysis. We benchmarked 6 sets of 5 parameters (each group was labelled as “c4”, “c5”, “c8”, “c10”, “c100” or “c200”; see Figure S1 and the P-clouds subsection in the Materials and Methods for the details for each parameter) using a 16 nucleotide oligomer size [63]. The most relaxed parameter set was c4 and the most stringent c200. To estimate how well each set of parameters fit the galGal4 genome, we examined the number of exons predicted by P-clouds for each set, but found very little variation between parameter sets (between 5.42 % and 5.91 % from c200 to c4). The highest repeat coverage was obtained with the least stringent parameter: c4. We concluded that the c4 set was suitable.

Fig. S1 P-clouds coverages using 6 parameters sets. Bars in blue represent the coverage in the P-clouds annotation corresponding to the repeats in the genome and those in red, that obtained for the exons.

Red is a recently published repeat annotation tool [15] and therefore does not have the same track record as P-coulds. It also evaluates the amount of repeats using a k-mer method but locates repeats using a machine learning approach. This program only requires the sequence of the genome model and an oligo size that is calculated using the same rule as P-clouds (i.e. 16 nucleotides, see above). The global amount of detectable repeats in galGal4 obtained with Red was 29.9 %, a value close to that obtained with P-clouds (33% see text).
