# Supplementary material for: Deep landscape update of dispersed and tandem repeats in the genome model of the red jungle fowl, Gallus gallus, using a series of de novo investigating tools
Source: BMC Genomics. 2016 Aug 19;17:659. doi: 10.1186/s12864-016-3015-5 (PMC4992247; doi:10.1186/s12864-016-3015-5)
Supplement: Supplementary file 25 [file 12864_2016_3015_MOESM2_ESM.odt]

# Additional file 2: Evaluating the efficiency of P-clouds, REPET, and Red.

The amount of repeats in the model genome  Anopheles gambiae (AgamP3)[^1] was determined using three programs: P-Clouds, REPET, and Red. Obtained repeat coverages were compared to those from RepeatMasker (RM) based on previous publications[^2],[^3]. The results are summarized in Table 1 and 2 below.

# Table 1 Amounts of repeats calculated by RM, REPET, P-clouds and Red in AgamP3.

|  | Coverage of AgamP3 in bp | Percentage of AgamP3 coverage |
| --- | --- | --- |
| AgamP3 | 277,645,527 | 100% |
| Repeat amount identified by RM | 41,794,934 | 15.1% |
| Repeat amount identified by REPET | 73,501,425 | 26.5% |
| Repeat amount identified by P-clouds | 74,964,293 | 27.4% |
| Repeat amount identified by Red | 78,322,870 | 28.2% |

# Table 2 Overlaps between repeat annotation obtained with RM, REPET, P-clouds and Red in AgamP3.

| Pair-comparison between files | Percentage of overlap in coverage of the first pair member by the second pair member |
| --- | --- |
| RM / P-clouds | 61% |
| RM / REPET | 100% |
| RM /Red | 84% |
| REPET / P-clouds | 61% |
| REPET / Red | 99% |
| P-clouds / Red | 62% |

# These results indicated that REPET and Red repeat coverage overlapped entirely (100%) with those of RM. In contrast, even though P-Clouds reported percentages of repeats similar to those calculated by REPET and Red these repeats did not entirely overlap  with the RM annotations and included a large number of repeat annotations that were specific to P-Clouds. Similar results were obtained with the dmel6_07 model[^4] of the Drosophila melanogaster genome (data not shown). Finally, and in agreement with the above observation,  P-clouds has been reported to only annotate 57.3 % of repeats contained in the official TE annotation of Arabidopsis thaliana[^5]. These results led us to abandon the use of the P-clouds method and to use Red instead. Furthermore, Red has the advantage of having 100 to 1000 folds faster calculation time than REPET, RepeatScout and RepeatModeler packages[^6]. 

[^1]: VectorBase : Bioinformatics Ressource for Invertebrate Vectors of Human Pathogens. https://www.vectorbase.org/ (2007) Accessed 2015 Sep 14.
[^2]: Arensburger P, Megy K, Waterhouse RM, Abrudan J, Amedeo P, et al. Sequencing of Culex quinquefasciatus establishes a platform for mosquito comparative genomics. Science. 2010;330: 86-88.
[^3]: Holt RAG, Subramanian M, Halpern A, Sutton GG, Charlab R, et al.The Genome Sequence of the Malaria Mosquito Anopheles Gambiae. Science. 2002;298:129.
[^4]: Flybase. [ftp://ftp.flybase.net/genomes/Drosophila_melanogaster/dmel_r5.55_FB2014_01/](ftp://ftp.flybase.net/genomes/Drosophila_melanogaster/dmel_r5.55_FB2014_01/) (1994) Accessed 2015 Sep 14.
[^5]: Arabidopsis thaliana TAIR V10 Tes. https://urgi.versailles.inra.fr/gb2/gbrowse/tairv10_pub_TEs/ (2014) Accessed 2015 Sep 14.
[^6]: Girgis HZ. Red: an intelligent, rapid, accurate tool for detecting repeats de-novo on the genomic scale. BMC Bioinformatics. 2015;16:227.
