# Supplementary material for: Deep landscape update of dispersed and tandem repeats in the genome model of the red jungle fowl, Gallus gallus, using a series of de novo investigating tools
Source: BMC Genomics. 2016 Aug 19;17:659. doi: 10.1186/s12864-016-3015-5 (PMC4992247; doi:10.1186/s12864-016-3015-5)
Supplement: Supplementary file 26 [file 12864_2016_3015_MOESM3_ESM.odt]

# Additional file 3: Features of SSRs in galGal4

# Simple sequences

# There were 189,543 and 14,891 loci respectively containing simple sequences, a polyA stretch (4372699 bp; 0.42% of galGal4) or a polyC stretch (283691 bp; 0.03% of galGal4) of at least 50 nucleotides (Table 3). PolyC stretches in galGal4 were approximately 13-folds higher than those of polyA stretches. This observation cannot be explained by the activity of the CR1 non-LTR retrotransposons because, in contrast to human L1 non-LTR retrotransposons[^1],[^2],[^3], avian CR1s do not contain a polyA at their 3' ends[60][^4],[^5]. Another source of AT-rich elements in eukaryotic genomes are the scaffold/matrix attachment regions (S/MARs, that cover 2.3% of the galGal4 genome). These functionally repeated DNA elements may be attached by a nuclear matrix where the RNA polymerase II complex would be located when they are involved in transcription[^6],[^7],[^8]. The intersection between the chromosomal positions of both kinds of elements revealed that polyA stretches only covered 1.4 % of S/MARs. Therefore, the numerous stretches of polyA in galGal4 were neither products of aborted events of CR1 retrotransposition nor parts of S/MAR elements.

# Microsatellites

# There were 770,202 loci containing one of the 2,101 microsatellites found in galGal4 (Table 3). Previously, Brandström et al [9] indicated that there were 1,615,000 microsatellites that were 6 bp or longer in galGal4.  Using a similar size threshold, we found 1,997,228 microsatellites using the Tandem Repeat Finder (TRF) program. This suggests that TRF is more efficient  than the sputnik program. Among microsatellites, we expected to find (TTAGGG)n repeats that are located at telomere ends. Our investigations revealed that only 8 kbps. of such repeats were detected in galGal4 while approximately 2-4% (i.e. ~21-42 Mbp) are present in the real RJF genome [42].  This confirms that telomere sequences are not present in galGal4 genome. To our knowledge, there has not been an analysis of minisatellites at the genome scale in the RJF genome since 1994[^9],[^10]. We identified 12,310 loci containing one of the 123 different minisatellites found in galGal4 (Table 3). With respect to their numbers and the length of their repeated units, the sequence diversities of microsatellites and minisatellites were rather low (2101 and 123 different repeated units, respectively). We verified whether this reduced variability was due to their involvement in S/MAR elements and found that they only covered 5.8 % of S/MARs.

# Large tandem arrays

# Analysis of large tandem arrays (Table 3) revealed that only 6 arrays contained less than 50 tandemly repeated units. Above this threshold, 10,136 loci containing repeats ranging in size from 60 bp to 2 kbps. were identified and were considered as satellite DNA arrays. Overall, these represented 0.24% of the galGal4 genome in spite of the absence of most large satellite DNA families from galGal4. Some of these were clustered  into 4797 to 8678 families using SiLiX[^11], depending on the similarity and overlapping rates used to align sequences (Table S1 and S2). This large number of families indicated that there were numerous satellite DNA families in the galGal4 genome that could be split in two types. Those that are abundant in telomeric and centromeric regions of some chromosomes (review in [5,44]) and that are widely absent in galGal4, and those described here that are numerous, but containing few repeats in galGal4. The distribution of these  satellite DNA arrays was  investigated in order to determine whether they were  concentrated near telomeres and centromeres, or whether they were interspersed along chromosome arms. Our results (Figure S2) confirmed that the density of these satellite DNA arrays were more elevated in microchromosomes and that they were distributed in the inner regions of the chromosome arms. Because not all the positions of centromeres in galGal4 chromosomes are currently available, this last observation could not be tested. Nevertheless, these observations suggested that the density profile, sequence diversity and distribution of minisatellites as well as the lack of repeated satellite DNAs in numerous microchromosomes might be a signature specific to avian genomes [44,101].

# Objet OLE

Table S2. Diversity of satellite DNA in Galgal4.

| Parameters used for the clustering* | 70 / 70 | 80 / 80 | 90 / 90 |
| --- | --- | --- | --- |
| Numbers of clusters | 1043 | 853 | 568 |
| Number of loci in clusters | 6233 | 4000 | 1877 |
| Range of loci per cluster | 2 to 600 | 2 to 301 | 2 to 70 |
| Numbers of single loci | 3754 | 5987 | 8110 |
| Numbers of satellite DNA families | 4797 | 6840 | 8678 |

# * Similarity rate / overlapping rate used to compare two sequences

# Fig. S2 Distribution of satellite DNAs in galGal4 chromosomes. The size scale of chromosomes 1 through 16 is shown on top, the scale for others chromosomes is on the bottom of the graph. Red bars represent regions containing satellite DNA arrays. Background areas in pink contain the 9 macrochromosomes plus the W chromosome.  The green background indicates all galGal4 microchromosomes plus the Z chromosome. Due to its size,  chromosome 32 data are not shown.  Chromosomes maps were drawn with a custom designed software: DensityMap.pl (available at [http://chicken-repeats.inra.fr/](http://chicken-repeats.inra.fr/)).  This script uses windows to calculate densities of satellite DNA arrays in chromosomes 1 to 16 and other chromosomes that were 100 kbp and 10 kbp. respectively. 

[^1]: Ichiyanagi K, Okada N. Mobility pathways for vertebrate L1, L2, CR1, and RTE clade retrotransposons. Mol Biol Evol. 2008;25:1148-1157.
[^2]: Kaessmann H, Vinckenbosch N, Long M. RNA-based gene duplication: mechanistic and evolutionary insights. Nat Rev Genet. 2009;10:19-31.
[^3]: Monot C, Kuciak M, Viollet S, Mir AA, Gabus C, et al. The specificity and flexibility of l1 reverse transcription priming at imperfect T-tracts. PLoS Genet. 2013;9:e1003499.
[^4]: Coullin P, Bed'Hom B, Candelier JJ, Vettese D, Maucolin S, et al. Cytogenetic repartition of chicken CR1 sequences evidenced by PRINS in Galliformes and some other birds. Chromosome Res. 2005;13:665-673.
[^5]: Liu GE, Jiang L, Tian F, Zhu B, Song J. Calibration of mutation rates reveals diverse subfamily structure of galliform CR1 repeats. Genome Biol Evol. 2009;1:119-130.
[^6]: Girod PA, Nguyen DQ, Calabrese D, Puttini S, Grandjean M, et al. Genome-wide prediction of matrix attachment regions that increase gene expression in mammalian cells. Nat Methods. 2007;4:747-753.
[^7]: Arope S, Harraghy N, Pjanic M, Mermod N. Molecular characterization of a human matrix attachment region epigenetic regulator. PLoS One. 2013;):e79262.
[^8]: Harraghy N, Calabrese D, Fisch I, Girod PA, LeFourn V, et al. Epigenetic regulatory elements: Recent advances in understanding their mode of action and use for recombinant protein production in mammalian cells. Biotechnol J. 2015;10:967-978.
[^9]: Bruford MW, Burke T. Minisatellite DNA markers in the chicken genome. I. Distribution and abundance of minisatellites in multilocus DNA fingerprints. Anim Genet. 1994;25:381-389.
[^10]: Bruford MW, Hanotte O, Burke T. Minisatellite DNA markers in the chicken genome. II. Isolation and characterization of minisatellite loci. Anim Genet. 1994;25:391-399.
[^11]: Miele V, Penel S, Duret L. Ultra-fast sequence clustering from similarity networks with SiLiX. BMC Bioinformatics. 2011;12:116.
