# Supplementary material for: Deep landscape update of dispersed and tandem repeats in the genome model of the red jungle fowl, Gallus gallus, using a series of de novo investigating tools
Source: BMC Genomics. 2016 Aug 19;17:659. doi: 10.1186/s12864-016-3015-5 (PMC4992247; doi:10.1186/s12864-016-3015-5)
Supplement: Supplementary file 27 [file 12864_2016_3015_MOESM4_ESM.odt]

Additional File 4: Diagram showing programs that are componanents of REPET: TEdenovo and TEannot. The programs below were run either successively or in parallel from top to bottom as indicated by the red triangles. The three programs in TEannot with blue or pink backgrounds were removed from our analyses and the outputs of the program in yellow were verified by hand. Note: RepeatMasker is part of the TEannot component.
