# Supplementary material for: Deep landscape update of dispersed and tandem repeats in the genome model of the red jungle fowl, Gallus gallus, using a series of de novo investigating tools
Source: BMC Genomics. 2016 Aug 19;17:659. doi: 10.1186/s12864-016-3015-5 (PMC4992247; doi:10.1186/s12864-016-3015-5)
Supplement: Supplementary file 28 [file 12864_2016_3015_MOESM6_ESM.odt]

# Additional File 6: Intersections between annotation files calculated with P-clouds, TRF, and REPET.

# Overlaps between the annotation files calculated with P-clouds, TRF (SSR), REPET (TE+DM), and CNVs [11]. Values are percentage of coverage in galGal4.
