# Supplementary material for: Deep landscape update of dispersed and tandem repeats in the genome model of the red jungle fowl, Gallus gallus, using a series of de novo investigating tools
Source: BMC Genomics. 2016 Aug 19;17:659. doi: 10.1186/s12864-016-3015-5 (PMC4992247; doi:10.1186/s12864-016-3015-5)
Supplement: Supplementary file 29 [file 12864_2016_3015_MOESM8_ESM.odt]

# Additional File 8: Diversity of CR1 elements within galGal4

# As of db20140131 Repbase[^1] reports that CR1 are divided into 22 subfamilies in the RJF genome. Other studies, based on the 3' ends of CR1 copies, classified them in 57 sub-families2. We reinvestigated this by performing two successive single linkage clusterings using SiLiX to gather copies in a cluster fixed at a minimum of 80% overlap and 80% sequence identity, as recommended in Wicker et al [60]. The first clustering was performed using 308 CR1 consensuses, 82 of them having a small size that excluded them from the process. This gathered 226 consensuses within 8 clusters, 7 of them matching with the Repbase sub-families CR1-C, CR1-D, CR1_F, CR1-G, CR1_GG, CR1-H, and CR1-Y (Table S2). The eighth cluster was named CR1_like. I This division into 8 CR1 subfamilies was conserved for the step involving resolving and merging stacked and juxtaposed annotations. Indeed, it avoids creating false CR1 copies in the final [TE+DM] annotation of galGal4 by fusing juxtaposed annotations that were defined from consensuses related to different CR1 subfamilies,. For the second clustering we used 226 genomic copy consensuses containing the last 1000 bps located at the 3' end of the CR1 sequences. Our results confirmed that copies belonging to one of the 6 clusters matching CR1-C, CR1-D, CR1_GG, CR1-H, CR1-Y and CR1_like sub-families were suitably clustered. Our results also showed that copies in clusters belonging to the CR1_F and CR1-G sub-families were gathered into 2 clusters that were different from those of their annotation consensus. Taken together these results suggested that there are  numerous CR1_F/CR1-G chimeric elements in galGal4. The emergence of chimeric elements between non-LTR retrotransposons [^2],[^3],[^4],[^5],[^6],[^7] followed by their amplification via retrotransposition is not a rare phenomenon in eukaryotic genomes. At the genome evolution scale  we would suggest that 22 to 57 previously described sub-families may have originated from frequent emergences of chimeric CR1 elements originating from a limited number of CR1 sub-families.

Table S2 Features and diversity of CR1 models found in the Galgal4 model based on the REPET and DM annotations (STEP3 + STEP4, Figure 2) after stack resolving and merging stacked and juxtaposed annotations.

| Names of TE models | a | b | c* | d | e |
| --- | --- | --- | --- | --- | --- |
| CR1-C | 1 | LINE | 27113 | 4.3547 | 0.6970 |
| CR1-D | 1 | LINE | 56909 | 9.1403 | 0.9380 |
| CR1_F_NV2 | 2 | LINE | 36222 | 5.8177 | 0.8303 |
| CR1-G | 2 | LINE | 9635 | 1.5475 | 0.2885 |
| CR1_GG | 2 | LINE | 10791 | 1.7332 | 0.3133 |
| CR1-H | 3 | LINE | 12544 | 2.0147 | 0.4253 |
| CR1-Y | 1 | LINE | 211266 | 33.9320 | 7.4710 |
| CR1_like | 296 | LINE | 49377 | 7.9306 | 0.8823 |
| Total | 308 |  | 413857 | 100 | 11.8457 |

a, Consensus Number,; b, TE types; c, Total number of TE copies; d, Percentage of the total number of TE copies; e, Percentage of chromosome coverage; 

* Post stack resolving and annotation merging include all complete elements, internally deleted elements; 5' or 3' truncated elements and elements truncated at both ends (i.e. internal regions of a TE devoid of ends).

[^1]: Repbase at giri. http://www.girinst.org/repbase/ (2001). Accessed Sep 10 2015.
[^2]: Losada A, Abad JP, Agudo M, Villasante A. The analysis of Circe, an LTR retrotransposon of Drosophila melanogaster, suggests that an insertion of non-LTR retrotransposons into LTR elements can create chimeric retroelements. Mol Biol Evol. 1999;16:1341-1346.
[^3]: Buzdin A, Gogvadze E, Kovalskaya E, Volchkov P, Ustyugova S, et al. The human genome contains many types of chimeric retrogenes generated through in vivo RNA recombination. Nucleic Acids Res. 2003;31:4385-4390.
[^4]: Gogvadze E, Barbisan C, Lebrun MH, Buzdin A. Tripartite chimeric pseudogene from the genome of rice blast fungus Magnaporthe grisea suggests double template jumps during long interspersed nuclear element (LINE) reverse transcription. BMC Genomics. 2007;8:360.
[^5]: Buzdin A, Gogvadze E, Lebrun MH. Chimeric retrogenes suggest a role for the nucleolus in LINE amplification. FEBS Lett. 2007;581:2877-2882
[^6]: Bao W, Jurka J. Origin and evolution of LINE-1 derived "half-L1" retrotransposons (HAL1). Gene. 2010;465:9-16.
[^7]: Luchetti A, Mingazzini V, Mantovani B. 28S junctions and chimeric elements of the rDNA targeting non-LTR retrotransposon R2 in crustacean living fossils (Branchiopoda, Notostraca). Genomics. 2012;100:51-56.
