# Supplementary material for: Deep landscape update of dispersed and tandem repeats in the genome model of the red jungle fowl, Gallus gallus, using a series of de novo investigating tools
Source: BMC Genomics. 2016 Aug 19;17:659. doi: 10.1186/s12864-016-3015-5 (PMC4992247; doi:10.1186/s12864-016-3015-5)
Supplement: Supplementary file 30 [file 12864_2016_3015_MOESM9_ESM.odt]

# Additional File 9: Nucleic acid sequences of retroCalimero, retroSaturnin and retroTux.

# A, retroCalimero

# TGTGGAGAGATTTATTGATGACTGGCTGACTGAGAAAAGCCATGCAGACACTGTGCAGCTGGTTAAGCAACCTTGTTACGCGCAAGGTCAGGTATGAGAACTAGGAACAAAACAGGATGCCGATGGAGAAAGCCGGCAACAGTGTTTTGAGAAGTCTGTAAGAGAGTGTTTGCTGAGAGATAACCACAGAGCACAAAGATAGGCGTAGTTGACACTGACTAGCGAGCCAATCCTGAGCTCCACTTTAGCAATATGTATGAGCCTATTATCCACACTATAAATGTACACGCAGCTGAAACAATAAACGAGATTTGCTGATCATCTACTATGGTCGTCGCATTTCTCCCGCTGATTTCCTACAAATGGTGACCCCGACTGACCCAGTAACGGCGCACCACGGTGGGACGGCGTAGGAGTTCGGGTCCTAAAGCAGTGAGGACGGCTAAAGCACTCAGGAACCAGAGATAGGAGTGCCGAGCCCTAGGTGACCTCATCGGAGAGCCTGGCCGATACGGGCCGCCGAGACCTTGTAAGGCTGCAATAGCGCGCGCTGACAGATAAGGGATGGAAAGGCAAGCGGCTTATGATTTATTTACTTGTTTTCTGAGAAAACGACAAATTAAGGGAGTAGACTTACAGAAGGAACTCCCCGGGTTATTAGCTTATGGCAATGCTAAAGGGTTTTTTCAAAACCCACATTTAGTACATGACCTTACAGAATGGCGTAAATTTGGGGATGCAATTTGGCAAGCAGTTTTAGATGATGATAAGACAGCAAAAAAATGCGGGAAACTGTGGCGAGCAGTTCACAATGAACTGTTGCAGCACCAAGCAGAAAAGAAGGCAGCGGAACAGGCCAGTGCTGCACAGGGGAGAAATAAGGATTACAATGGGTGGCCGGATTGTCCGTTGCCCCCCGCAGTGACTACCTTAGTGTTGCCGCCGGCCGCGGAGCACGCCACATCAGCATGTTTGGAGCCAAGCGCCCCACCCCCGCCCCTAGTTACGGCCGCGCCGGGTAGTTCACCTGCGCCTATTACAATGGGCCCAATAAACCCTCCGAGCAGTGAGCCGATCCCTGGGGCAGAGAGTGACCTAGCGGGGGCCATTGCACGGGAGAGGCGGGAAGCTTGGGCGGCGCTGGCACGGGCCTGCATGGAGGCGGGGGATAACGACGCAGTGGAGGCCGTGCGGCACATGGCGTTTCCGGTGATATATGCCTCTAATCCTGCGGGAAGGATGCAGGCCACCATCACAGCTTTAGATTGGAAATTATTATCACAGCTACGATCTACAGTTAGTCAGTTTGGGGTAAAAAGCGAGCCAGCTAAGCAGATGTTGGATTATATTTGGAGTACGCAGATATTGCTGCCATCCGATTGTTGGGCAATAGCAAAATTGATCTTTTCACAACATCAACAGCTGTTGTTTAATGCATATTGGCAAGAACTGTGCCATCAGAGTGTCTCAAAGGCTAGGCAGCCGGGAGACCCACTACATGGTGTAACTATCAAAGAACTCTTAGGGCTAGGGCCTTTTTTCAGAACACAAGCCCAAGCATTATTAGGGCCAGATAAATGTCGAGAAACTATGTATTTAGCTAGACAGGCCATGGACAAGATTAAGGTGCCTGATGGATTGCCATTTTATATGGGGATCCGACAAGGTAGAGATGAGGACTTTGGGGCATTCATAGACAAGGTAGCCGGGGCTATTGAAAAGGCAGGGGTGCCAGAGTATATGAGAGGAGTGATGTTAAAACAATGCGCGCTCCAAAATTGTAATTCAACAGCACGTAGTATTCTGAATACTTTGAGGAGTAATTGGACTATTGAGGAGGCACTGGAAAAGCTATCAAGCGTGCCGGTCGGGCCCCAAGCATTTCTGGTTGAGGCTATTAAGGAGTTAGGGGCAGGTTTAAAGGCGCAGGCTGAGGCCTCTCACAATCAAGTGCTAGCAGCTCTTGCACCCTTACAAGCGTCTGCGACAACAAATTTAGGCCCGAGGTCGCCTGTTGCTGGTCGCATCAAATGCTATCGCTGTGGTGGCATGGGACATGTGCGTCGTCAGTGTCAAGCCACCGGTTCATGGTGTCAGACATGTCGTATGGACAACCACAATACCAATGCCTGTCGACGCCGGTCGGGAAACCCCCAGCCCAGCGCGAGAAAGAAACGATGGCCGCGCGCAGACACAAGTAGCCGTTTCCAGCCAGCAGCAGCCCTGCAACCAGCCACACCAGGAAGCCTTGGCTTGGACTTGGCAGCCGCAGTGACCACGACCTTAATGACCACAAAACCTGAGCGGGTGTCTACGGGGATCAGGGGACCAGTAATGATAAATGGAACCGCCGTTGGGGCCCTTCTATTGGGGCGTTCTTCAGCATCGATGCTCGGACTTTTTGTCCTCCCTGGGGTAATAGATGCGGACTTTCAGGGCGAGATCCAGATTATGGTATACACCCCGTTTCCTCCAATAAAAATTGAGAAAGGGCAATGGATAGCGCAGTTAGTACCCCTAGAGCAATTGACCAAAGCCTTAACCCTGTGTCAATTGTCTCCCTGAGGGGAGCAAGGGTTTGGCTCCTCAGGGGGCTTAGCATTGCTATCACTAAATTTGCATGATCGACCTAAAAAACCAGTGACACTTAAATACAGAGAGGAAGAAATCAAACTTCAAGGTCTATTAGATACAGGGGCCGATAGCAGCATAGTGAGCCCAGAAATTTGGCCGCCTCATTGGCCACTGCAAGCGGCCATAGCCACAGTGACGGGTATAGGCGGACTATCCTTAGCGAAAAAGTCGCCTCCCCTGCAAATCCACCTTGATGAACAAGTAGTCCATACCTCTGTCTCGGTGGCACCCCTACCCCCCACCGTTCAATGCTTAATTGGCCGCAATGTGCTCTCTCAACTGGGGGCACTTCAACCCGGCCTGCCCAATCCTGCCATGCTACCTTACAATTGGCCATTACTAATTATTGACTTAAAGGACTGTTTCTTTACTATTACCCTGCACCCTCAGGACACTAAACGATTTGCCTTCACATTGCCTGCATTGAATCGAGAACACCCTGATCAACGATTTGAATGGACAGTATTACCTCAGGGGATGAAAAATAGCCCCACGCTATGTCAACTATATGTCGATCATGCTCTACAACCACTCCGGCGAGAATGGAAACAAATGGTCATTTATCATTACATGGATGACATCCTTTTTGCCCAGCCAGAGGCCTTCACACAGGAACAAATTTGGCAAATAGAAAAGACCCTAAATAGGGAAGGACTTATGATTGCCCCTGAAAAGGTACAACTCTCTGCGCCCTGGAAGTACTTAGGATGGACACTGACTAACACGATAGTAACCCCACAGAAACTGCAACTAAATACTAAAATAGAGACTCTACACGATGCCCAAAGGTTACTGGGGGACTTACAGTGGCTGTGCCCTGTGGTGGGCATCCCAAACGAACTCTTAGAGTCGTTGCGACCTTTGTTACGGGGCACTGACCCGGCCCAGCCTGTAACGGTGACAACGCAGCACAAACGTCTACTACAACAGATTATGGACTGCATTATACACGGCAGTGTTCGGAGACGTGACCCTGACCTCCCCATACAGGTTATGGTATGGTATGGACCAAAGTATCTTTTAGGAGCGTTGGCACAATCTAAAAAGAAAACGGGGGAGGTATGGGTACTAGAGTGGATCTGTCCCTCACTGCAGCAATCAAAAACACTTCTTCAAAAAATTGAGCTCCTGGCAGAAGTGATTAAGAAAGGGCGAGAACGTACCCTGCAAATCACAGGTATGGAGCCTGTGTGTGTACAGCTGCCAATGCAGAAGGACACTCTGACATGGTATGTGCAGCATAGTCCAGAGTTACAGGATGCTCTCTTACGAGCTGGAAGTACGGTTTCAATGGAAAAGATTCCGAACGCGCCGCTACATTGGATTGGTCAATGGAGTTGGCTCCGGATACCAAAGCGGCCCGAGACGCCCTTGCAGAACGCAATCACGGCTTACACGGACGCGGGATGGAAGTCTAGAACAGCAGCAGTGACCTGGCAGCAGGGCGGCTCCTGGAGACATCACCTCATTGCAGCCGATGATAAGGACTCATTGCAAACATTTGAGCTGGTGGCCGTTGTATGGGCCATGATGAACTTAATCGACCCTCTTAATGTGGTCACCGACTCCCTTTATGTAGCTGGAGTATGCCACCGAATAGAGGAGGCCTACATAAAGGAAGTGCAGAATCGGCGGCTGTACGAGCTGTTCGTGCAGTTGCAGAGAGCAATCAGAATTAGGGAGCACTCATATGCAGTAATACATGTTCGAGGTCATAAATGGGAGATAGACTTGGGGGAGGGAAATGCGAGAGCCGATCGCTTGGTGTCGCTGGCACAGAGACCTTTAGTCTCCCAGCATGTCCTGGCCCGAGAGGCGCACTGTATGTTTCACCAGAATGCCAAGGGGCTAAGAAGGGAATATCAGATAACATATGAGGACGCTAAGGCAATTGTTAGATCGTGCCCAGTGTGCAGCCACCATAATGGCGGTATGGGTCTCGGGCTAGGAGTTAACCCCAGGGGACTTAAAGCTAACGAAAATAGGCAGATGGATGTGACGCATGTGGGTGAGTTCGGGCAGCTGAAATATGTACACGTGTCTATAGATACATATAGTCATTTTATGTGGGCCACCGCTCAACCTGGAGAAAAGGCTATGTATGTAGAAAGGCATCTCAGTTGTTGCTTTGCAGTTATGGGTATATCACTACAAATAAAAACAGACAGTGGCCCAGCCTACACTAGTCGTAGGCTTGGGGAGTTTCTGCAAACATGGGGAGTAAAACACAGTACGGGTATCCCGAACTCGCCCACAGGCCAGGCAATAGTAGAACAGGGAAATTGTGGATGGACGGCTGACCCAGCGAGGTCATGTTTTTCTCCCCCTGGGTCAGTGCAAGTAAAACAGCTGCTATGGATCACTGTCTCACACAGAACAATGATGGGAGTAGGCGAAAGGAATGCAGAGCTGCGATAGAGACCGCTGTGCCAGGTGCTGTGAGACAAGCTGCAAGCAACTGCAAATTATGAGAACTGCGCCAGCGCTGCCCTCTGTTCTTCTGTCTCATTATACTGCATGCTAATGGACTTTCAGGAACGTAATGAAAATGTATCTGAATTCCTGTCAACTATTGATTATGTATTAGTTTGACCTATATCTATAGCACAATTTCTCCTGACGGTGTGCAAGTTAGGTGGAGTGATCCCCCTTGCACTATCAATAGACATCAATAGACACATGCCTGCTCTATATCCTTACCGGATATAGGGTCTGATTTCCGCAACTCAATTTGCAACGAGAATAGATCCGCTCTGTTCGGCTGCGGGATCGGTTGAGAGAGGGGACACCTTTGGCGCGCCCCAAGATTTCTTGGAAGGTCTCCCTTCCTCACCCGATCACTGCAGGGACAGACAAGGACCATCCATTGGTGTATCAGGTATGTGTATAGAAAAGGGGGTGCCCGTAAGGCGTGAGGAGACGTCCTACGCAGGGTATGAAGGAGCGTGCCTGCTCCCCCCCCAGAGGTACGTGAGCTCACAGGTTAGGTTGCCGGCTGGGGTAGGACTTGCAACTAATTGACTAATGTACTGGCGTTATTAAGTTTCAAGCTAAAAGACGTTGTCAGCATTGTGGATTATGTATAAACCATGTTTTTTGTTCTAAATATCAGAGTGAGTGTGGTGTGAGCGTGTAAGGGAACGTGGACAAGCTTCTGTGAGATCTGTTCCCAAGTGTTTATAACTGAACAATATCACTATACGGTGTTAAGGGTTTGTGGAAACTTTGAGGGAATTAGGAATCAATAAGAATTTTGTAAACATCATGGGAGGCTCCCAGGAGAAAGTTGTGTTTCCACAGGATCTGGCCCATTATGGGTTCCCAGTAAATGGACTAAGCCAGCCCCAAACATTAGCAACCCATCTCAACCTGATGACAACAACAGCGACTCCGGCGAGCAATAGCCGCATGGTGTGCAAGACATCATATACAGTGCCATGACTCCCGAGCTGCAACTGGTATTCCCTTAGCAGATTGCAGAAAACATCTCATCAGAAAGGTCTCTTGGACACTGGGAACTGCCAGGAGCAATTGGCGGTTACGAGACTCAGAAGCGCACTAATTGTGGGGGTCGGGTACAGTGATTGATCCAGAGTGGGGACTGTGGTATTTTATTCATCGGGATATTGAAACTGTAGTTAATACTGGTGTGCTGTGTAGTCTTTACTGTTAGTTTACTGACAAGGAATGCTTTGTAGAAGTGTGTAACATAAATCTAGTGTATTCTTATAGATAGTGATTGGTATGCTGATAATCATGCTTTGTTTACTGTAATGCTAGCAAAAGATGGTTCAAGATGCAATTCATACAACCTGGCTCGTACAAAAAGAAAACGGGGGAAATGTGGAGAGATTTATTGATGACTGGCTGACTGAGAAAAGCCATGCAGACACTGTGCAGCTGGTTAAGCAACCTTGTTACGCGCAAGGTCAGGTATGAGAACAAGGAACAAAACAGGATGCCGATGGAGAAAGCCGGCAACAGTGTTTTGAGAAGTCTGTAAGAGAGTGTTTGCTGTGAGATAACCACAGAGCACAAAGATAGGCGTAGTTGACACTGACTAGCGAGCCAATCCTGAGCTCCACTTTAGCAATATGTATGAGCCTATTATCCACACTATAAATGTACACGCAGCTGAAAGAATAAACGAGATTTGCTGATCATCTACTATGGTCGTTGCATTTCTCCCGCTGATTTCCTACA

# B, retroSaturnin

# TGTGGAGAGCCTGTACACAGAGTGGCTACGTGACAAGGGCCACAATGGAATGCCACTCGTGAAACAATAAAAGAAAGTATGTAACTAGAGGCAAGGACGGCTACTCGGGTAGATGGAAAACAGGATGCATATCTACAGCAATGAAGAAACAGTAACAGCAAGGCTAACCACGAAGGTCAAGATGAAGTAATGCATAATCCGCCAATCCTGAGCTATGCTTTTGCAATATGTATAAGCTCATTACCTACTGTATAAATACAATACCAATGTGCCTAATAAATGAGACCTGTTGATCATGATAGCTTGAGACTCGTCTCCCGCGGTCTTTTCCGACAAATGGTGACCCCGACGTGATACAGACCGGGGAACTTTGGCTGCCACGAACGGAGACTGCGTTGGGTTCGGGTCCCGCAGCTAGACGGACGGCGAAAGAGTGAAATATTGGACTCCGCGCCTTGGGCGACCACAGCGGTGGGCTCAACCGATACGTGCTGCCGAAGCTTGGGGGGCTGCAACAGCGCTGACGTAGGTAAGGATGGACAGGCAAGCAGCATATGATTTATTCACTGCCTTTCTGAGAAAGAGGCAATTCCGGGGGGTAGACTTAGAGAAGGAGCTCCCTGCCCTGCTGAGTCATGCAGTATCACACGGGTTTTTCGCTGACCCTCACTTAGTACACACGCTAGATGAGTGGCGACGGTATGGGGATAAGCTTTGGGAGTTGGTGTTGGAAGATGACAAGGTAGCAAAAAGGATGAGTAAGTTGTGGAAGGCAGTGCATAACGAGTTGTTGATGGGTCAGGCGGAGAAGAGGGCTGCGCAGGGAGCGCAGGCAGCTCAAGAAAAGAACAAAGATTATGGGTTGATCTGGGATAACACTCCAAACCCCCCCTCCTTTTGCATGGTTATGCTACCCGCTGCTCCCCCCGCATCCGCAAATGGAGTGCAGGCTCAGTCAACCGCAGAGCCTGTGGAGGCACCCCCGCGTCGGGTTTTAGGGCCATCAGCATTAGATGGTTCCGGGTCGGTTCCCGGTGCCGAGTCTGACCTTGCGGAGGCTATAGCCAGGGAGAGACGGGAGTTGTGGACCACGTTAGCAAAACATGGGATGGAGGACGGAGATAATGAGCTTGTGCAGGCAGTGACTGAAAATCTTGCGTTCCCAGTTGTCTATACTCCCACGCAGCAGGGCGGGTTACAAGCAGAAATTCGAACTTTAGATTGGAAAATGCTGTCCCAACTACGGGCTACAGTGGGGACATATGGGGTAACCAGTGAGCCGGCTAGACAGATGATGGAATATTTATTCAGTGCGCATATACTACTCCCGGCCGATATTAGAGGCATAGCTAGATTAATTTACACCCCCCATCAACTAATATTGTTTAACGCACACTGGCAGCAGGAAGCTGCAATATCGGCAGCAGTGCAGAGGGGTCCGGGAGACCCTCTGGCAGGCATAACAATAGAACAGTTGATGGGTCTCGGGGCATGGACTCGGATAGAGGCACAGGCAATTTCGGGACCTGATGTTTCCCGAGAAATAATGGCTGTAGCCAGACGGGCTATGGACAAGATAAAGGCTCCTGGAGGTACACCTATTTACATGGGGATACGACAGGGGCGGGAAGAGCCGTTGGGTGATTTTGTGGATAAGGTTATGGATGCGATTAAAAAGGCTAGCGTACCAGAGCACATGCAGGGGGCATTGCTTAAGCAGTGTGTGCTTCAGAATGGGAACTCGAATACTCGGTCCCTTGTCAATACCCTGCCTGGGGATTGGTCGATCCCGGAGCTACTGGAAAAGGCTGCTACGGTCCCCTCGGGGACCCAGGCTTTCCTAGTGAATGCCCTGCAGAAGATTGGGGATGGGTTACGGGAGCAGGCCAGAGCATTGCAGGAACAATCAAGGTCAGCTCAGACCCAGGTGTTAGCAGCCCTTGCGCCCCTCCAAGCTACCGTCTCCACTGGCCCCCACCCGCGCGGCAACCCCCGCATGAAGTGCTTTCGCTGTGGGAATGTCGGACACATCCGCCGTGAATGCCAAGCAGATGGGGTCTGGTGCGGCAAGTGTCAATCGAACACCCACAACGCCAACGCCTGCCGCCGAAAGTCGGGAAACGCGAGGAGCAGCGCGAACAGCAGCCGCGCCCGGACACAAGTAGCAGCTGCAACATCGAACGCCCCCGGCAGCAACAACCCCAGCCTGCCACAAGCGGGAGCCTCGGCCTGGACGTGGCAGCCTCAATAGACATTACCATCTTATCTAATCAACCCCACCGAATTGCTACCGGACTGTTTGGCCCTGTTATGATAAATGGACAAGCGGTGGGAGGGCTGCTGCTCGGAAGATCATCAGCCAGCATGTTAGGACTATTTGTCTTGCCTGGGGTCATAGATGCGGACTACCAAGGAGAAATTATGGTTATGGTGCATACCCCTTACCCGCCAATAAGGATTAACAAGGGGCAACGGATAGCTCAGCTAGTTCCGCTGCCGCAACTAACGAAAGGGATGATACCCCTAAAGCAGGAACCGAGAGAGCAAAAAGGATTCGGTTCGACTGGGGGACTAACTCTGCTCACTATTGACCTGTCAGATCGCCCAAAGAAACCATGCACATTGTACTACCGAAATCAGAGCATTATTCTGACAGGATTATTGGACACGGGCGCAGATACATCGATCATTACCCCAGGAGAATGGCCGTCGGAATGGCCACTTCAATCGTCCACTACCACAGTGACCGGGGTTGGAGGAGTGACGTTAGCAAGCCGGACTCCCATGCTCGCGGTAGAAATAGACGGCCGAAGAGCCACAGCTGTGTTTTCACTAGCCCTGCTTCCGCCCACAGTATCCTGCCTTATCGGTAGAGACGTGTTGGCTCAATTAGGCCTTGTCCTTACCAATGAACACCCTTTAGGATAACGGCCATTGCTTGGACTTTCCCGCTGCCACTTACATGGACTACGAACAACCCGGTTGTGGTTAAGCAATGGCCACTAAAACGGGAAAGTCTTCTACAAGCACATCAGTTAGTGCAAGAACAATATGCTCAGGGTCACCTGAAGTTGTCCACCAGCCCATGGAACACCCCAATATTTGTGATTCAAAAGAAATCCGGGAAGTACCGTCTGCTGCACGACTTAAGAGCTGTTAATGATCAGATGGAGCCCATGGGGGCTCTTCAACCCGGCCTGCCTAATCCTGCCATGTTACCTGAAGACTGGCCTATCCTTATCATCGACCTCAAGGACTGTTTTTTCACAATTGCTTTACACCCCCAAGATACGAAACGTTTTGCCTTTACCTTACCGGCAATAAATAGGGGGGAGCCTGATAAGCGCTTCGAATGGACAGTCCTACCACAGGGGATGCGTAACTCCCCAACTATTTGCCAGTTGTATGTTGATGCTGCTTTACAACCGCTACGTAAAGAAATGCCCAACACTATCATCTACCATTACATGGATGACATTCTCTTCGCCCAGCAGGATCCGTTTACGGAGCAGCAGATTGAACGCGTCAAAACAGTTCTAGCGGAATTCTCTTTGGTGGTTGCTCCTGAAAAGGTGCAAAGGTCTGCACCTTGGAAATATCTCGGATGGCAAATTACAGGGAAGCAAGTAACACCACAAAAATTGACTCTAGCTACTGACATCGCCACACTGAATGATGCACAAAAGCTCCTCGGAGACCTACAATGGTTGAAACCGGTCGTAGGTCTCCCCAATACATTATTAGAAAAACTAATGCCCCTGCTAAAGGGTACAGATCCTTGCACCCCCATCTCCCTCACATTAGAGCAACAGGAAACCTTACAAGACATTACTGACTGCGTCTGCAAGGGATTTGTCTCCCGCCTGGATCCTAACCTACCATTGGACCTTATGATTTGGAATAACAGGACACACCTTCTTGGTGCAATCACTCAATTCCGAAAGAAAACGGGGGAGAGGGTGCTGGAATGGCTCTCGCCACCTCTTCAAAAACGAAAAACAATAACCACAAAAATTGAGAACCTTGCAGCTGTTATTAGGAAAGGCCGCGTGCGAATATTGGAAGTCAGTGGTCGGGAGCCAGAGACTATTTATCTCCCAATGGAAAAGGCGACCTTGGATTGGTATTTGCTAAACTCGCTGGACTTAGCCGAAGCACTCCTAGCCTCAGGGGCTAGGGTAAAGTCAGGTCCACTGGTCCCGCGAGCGCTGCAGTGGCTTGGGAATGATTGCCAGACTGATCTCCAGTAGCTTTGCCGGTTTTGTTGCCCAGAAAGAAAACGGGGGAAGTGTGGAGAGCCTGTACACAGAGTGGCTACGTGACAAGGGCCACAATGGAATGCCACTCGTGAAACAATAAAAGAAAGTATGTAACTAGAGGCAAGGACGGCTACTCGGGTAGATGGAAAACAGGATGCATATCTACAGCAATGAAGAAACAGTAACAGCAAGGCTAACCACGAAGGTCAAGATGAAGTAATGCATAATCCGCCAATCCTGAGCTATGCTTTTGCAATATGTATAAGCTCATTACCTACTGTATAAATACAATACCAATGTGCCTAATAAATGAGACCTGTTGATCATGATAGCTTGAGACTCGTCTCCCGCGGTCTTTTCCGACA

# C, retroTux

# TGCGGAATCAAACTCTATAACCCAGTTAAGTTTTAAAGCAGGTATGTTTACTCAGCCGGCCGGGTGCAAGGAGGATCACTCCACCAAACTTGCACACTAGCAAGCAAAAACACTACAGATTTGTAAGCCAAGTCCATACATATTCAGCAGAATTCTTGGAACCCATCTACATATTCATTATCCTTCTGGAAATTCTGGAAATTTACATACTGCCCCTCCCCCCGCATGTGCGTTGTCCATAATTGGGGTCTCCCTCAGGTGGTCATGGGGATGAAGTCAGAAGTCTTCCTCACAAAGGCTCAACATCTTCCTTGGTCTGACCTTTTCACGGTGCATTCAAACTGGTCAAAGCCATTCATTTTGTATTGTTCCTTTCTTTGTGTTTGCGTAGCTGACCATGAAATGTTGAGATGTCGTTCTCCTTCCCTAGCTTTCAAAGCTAGCTTATTTAACCCTTATATGTTCAAACCCTAGACCCACCTGAGCTCCTGATCTCAATCCCCCATTTTCTGAAGTCTAGCAAAACCTTGTGCTAGATGATCCCATTTTGAGAAAGAGAATTCCTAAAATAACTCCCATTTTCCATCTTCTTTCTTAGCCTGTGTTTCTTCCAGTCCTCGTAGCTATTCTCTGACCTTTACACAGCTATAACATGCATCGCAGAGAAATACATACTAAGATAAGCAAAGCAATTACTATAATGCAAGCCATAAAGAGTTGCTTTAACCATGCAAAATTAGGTATCCAATTAGTGAGCTTTTTCCGTAATTCTCTAAAGCCAAAAGAAGTATCATCCAATGAGATTTGGTGAAGTAACTTGGTACGTTCTCATGAGGCTTGCAGGTCAGTCTCTATTCTTGGAGTCTCGTCTATATATAAACAACACCTCTGATTGATAATCATTCAATACTTCATCCCCCACCTGCATAGAGATTTCCTTTTGTACAGTATAAGATTGCCCACAAAGAATCTCATGCGGGCTTAGTCCTTCTTTGGTTCGGGTTTTAGTTCAAACTCTCAGAAGTGCCAAAGGCAGTGCCTGTGGCCAGGGAATCCCAGCCTCTTGGCCAAGTTTTACTATCTGCAATCTGATCAGGTAAATCATTTTTCCACCTGTCCACTTAACTGCAGTCTATAAGGTGTATGCAATTGCCAAACAATTCCCAATAATGTGCTTATTTGTTGGACCACCTGGGCAAGAAAACGTGGGCCTCTATCTGATGGGACTGCTAAAGGAACCCTGAACCTTGTAATTACTTCATATAACAGCACTTTCTTGGGCCTTATCAGTCCTACAAGGAAATACCTCTGGCCATCCTGAAAAGGTACCAGTTAGTACCAACATAAAGCGATACCCTCCTTTTCTTGGGAGTTCCGAGAAATCAATTTGCCATTGTTGCCCTGGAAGGTTCCCTTTCCCAATAGGCCCACTTGTACCCTGTTCCTGGTACTAGGATCGTACTTTGTAACGTCTGTTTGCCCAATACATTTTCTTATCCCCCCTTAGAACCAATTTCCATAGAATACTAAATGGCGCAACAAAGTGTCCGTCTGGGTGCTTATCCATCTGTCAGCTTGCTCTCATCCCCTCTCTTTAGAATATTTTACAGATTCTGACTCAGGTTCAGAAATGTTAAGTTTACCGTCTGGAATTAAAGCCCTCTTCAACTACCTGTTCTGCCACCTGATCCGCCATCTTATTTCCAGTTTCCTGTACAGTGTTACCTTTCTAATGTCCTTTACAATGTATAATAGCCACACTCTTTGGTAGATTTACATAATGCTGTTTTTCCCTGTGTGTGAGCAATCCTTGCTCTTTTCAGATGGTACTATAAGTGTGTAACATCCCATGTACATAATTAGCATCTGTTCATATATTTCACACTTTTGTCAAGGCAGCTATTTCAGCCTTCTGAGGGAAAGTCCCCACAGGTAATGATCGTGCTTCGATTACCTTGTCAGTAGCAGCTAATGCATATCCTGCCTTACGGTTTCCTTGTCTCACAAAACTGCTTCTGTCGGTAACCCAGGTGTCTTCTGCATCTAAGGATTCATCTTTCAGGTCCGACTGGCAACAGTCCATAACTTCAGTTGTTTCAAAGCAGTCATGAAATTCTGGTTCTCCGGGAGTTCCGCTAAGGAAAGAGGCTGGGTTGACAATATTAGTTACAATTATTTCTGCATCATCCTGCTTTACCAGGATTGCCTGGTATTTCAGGAACCTTTGGGGGTGAGAGCCAATGCCCCCCTTTTACCTCCAGTACAGTAGACACTGTATGAGATGTTAAAACTGCTATTCATAAATTTACTGGCTTCTTGAATATTGAGCACTAGTGCAGCCACTGCCCTCAAACACTCTGGCCATCTCTTACCCACTTCATAAAGGGGCTTTACCAACAGTCCATAGCTGTGTATCCACAGTTGGCACCATCCTGTCATTCCTGAGAATGTATGGAGATCTTTCCTGGCAGTCCTTAAGATTCAAAGTCCAGCTGTAACCTCCTATCCCAGACACGTCACTTGCCGTTATTTGTGCCTTCTGTTGAGAAACTTGATAACAAAGAAATCCAGCAAACTCACAGTCCATTACACACAGTTCTCTTTTGTTTCGTGGCATCCATGCTCTGCAGTAAAGTTCCACCAGGGGATGGAGGGATCCAAATCTCAGGCTCAAGAGTTGACTAGTTCTCAAAAATCATTGGGGTGTTCTCACAACCCTGGGTAACACTGCCCAGGTTAACTGAGTCTTTTTTTCAATATATTGGGGTTTTCTATTCAAAAGCAAATAACCTTAAGCTTCCTTTGGCCAAACCAGGCAGAAAAGGCATCCTTCAAATCTGGAAGGTAAACTCCACCTACTCATTCCCAAACTTGGTTAATAAAGTGTATGGATTTACCACCATGGAGCGTACGCATTCAACGATTCTATTACTTGCTCTCAGATCTTGTACCAAACTATAGCACTTGCTATCCAACTTTTCATCAGCAAGACTGGAGTATTGTATTTGGATTCACATTCAATTAATAACCTGAAATCCAGAAGTTATCTATTATTTTTTCCATCCTTCTACAGTTGCATATCCTCAAAGGGCACTGCTTTACCCTGACTGGGCTAGCTTTAGCTCTTGTTTAGAGCTGCATTTTGGCTCTACCAGGTATCCCCGATACCCACACTCCTAAACCTACCTTATTTAAAATTTCTGTAACTTCTGGAGGTACAACATTGTTTTTCCCAGTTTGTGTTAAAGCCAGACTTAGAATTTTCAATCAGTTAATATTGTTTGACCCTTAGCTCCAGTTTTATTGCATTTAATCTCTGCATCTATTTGTTCTAATAAGTCTTACCAGAATAACAGTTTCAGAGAACCTGTCATGTACAAGAATTTATGCATTCCTACTTATTTTCTCAGCCTATATTTAATTGATCCCAAAAGGTAAGCTTTTTCTTGCTGGGCAGTAGCTCCTACTACTGTTGCAAAATCATCTAAGAGTGGTAAGAGGTACCCGTATCCACCAGAAAATAAACCTCTTTATCCTGCTCTCCTAGCTGCAATTTAACCAGTGGATCTGATTTCTGTCCTCTTTTGTTCTGACATTCCTGTTACACCTTGCCTGCCTCAGCCTGCATCCTTCCCAAACTTTTCACTTTCCAAACCCTCATAAACTACCAAAATTATATTTCAGTTTTACCTCCTCCTCTCCAAAGGCTTCCAGGGCTAACACTGGAATTTTGGGTTTCCTAGATCTCTGCAGATGAAGGCTATTCTCACTTCCAGTGCCCAGTCCTCTCCACCTCCCCCCCCTTCACAGGTAACGCTGTTTACAACAGCAGTCTCTAACTCTGGCTCCCTCTCACTCTTAGAGTTCTGCTCATGGGGGAACCACGGGGGAGGGTGGGGGGGTGTTGCTCCCATGGCAGCGCAGATAAACTCACTTTCTCTTTTTCTCTTTGTCCACTATTCATACCTTCAGTTACACTTCTGAATACCATTATACCACCAAAAACGTTTGTCCCAGTCCCTTCAGAACCCATAAATCAAACAATATAGTTCTTCAGTTTCCATAAACACCCAGTACTTAGTCTCGTGATTAGAGCACTCAATTATAAAAACTGTTCATGTTACTTCATTTACCTTTCCTCCTTAAGAATAACAGTAACTGCAAGAAGGTATTATAGTTTAGGGTCCCATTCTGGGGCCACTTCTCATCATCTTCTAATTTAAATATTAACCACCATTGATTGTAATACTTGATTAATGTTTTCTTACTCAGGGTGCCTCCAGGTTCCCCTGCAGTATCCCACTGATATAGCAATACGCATCCCAAAGGGGTTTCTTTGGGAATTTCCTTCCCCTGGGAGCCTCCCATGATGTTTACAAAATTCTTATTGGTTCTTTACTGCCTTAACATTTCCACAGGTAACTTTCAATACTCTAAAATTTCCCCACACTAATCCCAAAAATACTCCATCCACGAGTCACAATGGTACTCTAGACATAAATGACACCACCAGCCCTGGTACACCACCTCACACACTAAACAAAGTATAACATAAATGGGCCAACATTCATTTGTTGGGTGGAATGTACACCTTTCCTCTGGTTCCTCTTCCCAATTTACAGAGTACAAACTCCCATTCTTATTCCAGTCCATAAAATACATACAAACAGTGGACAGACATCACAAAGAGTCATCTTTCACTTTGTCTGTCTCTGGCCGTTTCCCTCACGGGAGAACAGAACTGCGGATTGCGACTCCGCACACGCTCCGTGGCTCTGCCACATCCCACTCACTCACACATACACACAAATGCTGACTTAAGTACAATCTGAGTTCAAACACTTGAAAGAATGTTTTGAATGCATCAGTAGTTCAAATGCATGGTACAGAACATTTACAACAGATGGTAATACCACCTATGACTCCTACTATTATTGCCACAAAAAACCACTTAGCCATTTAACAAAACGCACTGTTACCAACCACCTAGGAGAGATTTATTACTCCTTTTTTTCCACACGTACAACTTACAGGTCCCCCTAGGCGCAACCTAACCCATGAGCTCATTACCTCAGGGGCAGCTCTATGGTTATACCCAGCATAGAACATCTCCTCACAACTTATGGGTTCCCCTTATTCCCATTCACATACCTGGTCCGCCGATAGATGGCCTTTGTCTATCCCTGCAGTGATCAAGAGAGGAAGGGGAGTCCTTCCAAGAAACCTTGGGGTGCGCCTGAGGTATCCCCTCCTCAGCTGGTCCTGCAGCCAGCCAGACAGTGTCCCATCTGGGGTGCCAAAATTGACATGCGGAATCAAACTCTATAACCCAGTTAGGTTTTAAAGCAGGTATGTTTACTCAGCTGGCCGGGTGCAAGGAGGATCACTCCACCAAACTTGCACACCAGCAAGCAAAAACACTACAGATTTGTAAGCCAAGTCCATACATATTCAGCAGAATTCTTGGAACCCATCTACATATTCATTATCCTTCTGGAAATTTGTTTACATACTGCCCCTCCCATGGTGCATGCATTGTCCGTAGTTGGGGTCTCCCTCAGGTGGTCATGGGGATGAAGTCAGAAGTCTTCCTCACAAAGGCTCAACATCTTCCTTGGTCTGACCTTTTCACGGTGCATTCAAACTGGTCAAAGCCATTCATTTTGTATTGTTCCTTTCTTTGTGTTTGCGTAGCTGACCATGAAATGTTGAGATGTCGTTCTCCTTCCCTAGCTTTCAAAGCTAGCTTATTTAACCCTTATATGTTCAAACCCTAGACCCACCTGAGCTCCTGATCTCA
